# An eco-compatible strategy for the diversity-oriented synthesis of macrocycles exploiting carbohydrate-derived building blocks

**DOI:** 10.3762/bjoc.13.110

**Published:** 2017-06-09

**Authors:** Sushil K Maurya, Rohit Rana

**Affiliations:** 1 Natural Product Chemistry and Process Development Division, CSIR- Institute of Himalayan Bioresource Technology, Palampur, Himachal Pradesh, 176 061, India; 2 Academy of Scientific and Innovative Research, CSIR- Institute of Himalayan Bioresource Technology, Palampur, Himachal Pradesh, 176 061, India

**Keywords:** carbohydrate, click chemistry, diversity-oriented synthesis, macrocycles, ring-closing metathesis

## Abstract

An efficient, eco-compatible diversity-oriented synthesis (DOS) approach for the generation of library of sugar embedded macrocyclic compounds with various ring size containing 1,2,3-triazole has been developed. This concise strategy involves the iterative use of readily available sugar-derived alkyne/azide–alkene building blocks coupled through copper catalyzed azide–alkyne cycloaddition (CuAAC) reaction followed by pairing of the linear cyclo-adduct using greener reaction conditions. The eco-compatibility, mild reaction conditions, greener solvents, easy purification and avoidance of hazards and toxic solvents are advantages of this protocol to access this important structural class. The diversity of the macrocycles synthesized (in total we have synthesized 13 macrocycles) using a set of standard reaction protocols demonstrate the potential of the new eco-compatible approach for the macrocyclic library generation.

## Introduction

Macrocycles offer very complex molecular architectures with a diverse range of ring sizes decorated with many functional groups found application in pharmaceuticals, agrochemicals, cosmetics and materials science [1–4]. Carbohydrate-embedded macrocycles represent an important class of macrocyclic compounds in which at least two bonds from a monosaccharide residue form a part of the macrocyclic rings and have shown important biological properties [5–12]. For example, macrocyclic aminoglycoside analogues have shown binding with the trans-activating region (TAR) RNA of the human immunodeficiency virus (HIV); an attractive target for RNA-based drug discovery [13]. Further, macrocyclic glycolipids have shown phosphatase inhibition, cytotoxicity and antiviral activities [12,14]. Generally, the synthesis of these molecules involves a multi-step construction of linear precursors incorporating synthetically compatible functional groups followed by a cyclization in the late stage of the synthesis. The cyclization of the linear precursor is usually achieved by utilizing various ring-closing reactions such as Diels–Alder reactions, [15] aldol reactions, [16] copper-catalyzed azide–alkyne cycloaddition, [17–18] macrolactonization, macrolactamizations, Staudinger ligation or transition-metal-catalyzed coupling reactions [19]. Recently, ring-closing alkyne metathesis (RCAM) [20–21] and ring closing metathesis (RCM) [22–31] have emerged as very powerful tools for macrocyclization including for the preparation of peptidomimetic [17–18,32] glycosides and macrocyclic glycolipids [11]. Similarly, the copper-catalyzed azide–alkyne cycloaddition (CuAAC) reaction has found wide application in medicinal chemistry [33], biology [34–35], polymer chemistry [36], carbohydrates [37–40], peptides [41–44] and in materials science [45–48]. There are several reports wherein different strategies have been developed and used for the synthesis of glycoconjugates [9,49–51], however, the combination of a CuAAC and a RCM reaction has been used very little and rarely combinations of these reactions have been used for the synthesis of sugar-embedded glycoconjugates [52–53]. Further, the linear syntheses of macrocycles based on multistep protocols are not cost-effective and the development of efficient, sustainable, greener and economical methods is highly desired.

Synthetic methods to produce a diverse collection of macrocycles are rare and usually produce only compounds with a similar skeleton [20,33]. However, to achieve a higher hit rate against a broader range of targets libraries of diverse collections of macrocycles are desired [54]. The various diversity elements of a given library should include the molecular size, shape, heteroatoms, functional groups and stereo chemical complexity for selective binding [4]. The diversity-oriented synthesis (DOS), an algorithm in organic chemistry used to generate diverse molecules that include two-directional coupling, ring expansion methods, multidimensional coupling and domain shuffling has been used for the synthesis of small molecules and macrocyclic libraries. Further, several DOS strategies based around build/couple/pair (B/C/P) were developed for the synthesis of compound libraries including macrocycles [18,55]. Carbohydrates as building blocks are inexpensive and easily available commercial products and are well-endowed with functionalities which enable them to establish catalytic sites as well as secondary binding sites [56]. The abundance of various functional groups in the carbohydrate precursor allows for easy access to multiple building blocks by incorporating diversity-oriented synthesis (DOS). These moieties can be easily furnished with alkyne or azide functionality with routine synthetic transformation protocols that allow facile access to mono- as well as poly-functionalized derivatives via CuAAC reaction. The approach enables the rapid synthesis of carbohydrate conjugates in which the heterocyclic triazolyl ring serves as a shackle for joining the carbohydrate building blocks. Further, these carbohydrate conjugates decorated with appropriate coupling partner can be paired through ring closing metathesis (RCM) reaction. Carrying out the metathesis processes in green solvents is a major challenge. Unfortunately, halogenated solvents such as dichloromethane (DCM), 1,2-dichloroethane (DCE) or aromatics such as benzene and toluene are the most frequently used solvents for metathesis reactions whereas these solvents possess serious health and environmental hazards [57–58].

Here we report a novel application of the popular build-couple-pair (B/C/P) strategy [4,18,54–55,59–60] for the synthesis of sugar embedded macrocycles by iterative use of carbohydrate derived building blocks armed with azide/alkyne–alkene functionalities. The building blocks were coupled via 1,3-dipolar cycloaddition (click reaction) iteratively through the development of a greener base-free Cu(I)-catalyzed azide–alkyne cycloaddition reaction. The cycloadducts were then converted to macrocycles by Ru-catalyzed cyclization reaction using greener and non-hazards reaction conditions.

## Results and Discussion

**Figure 1:**
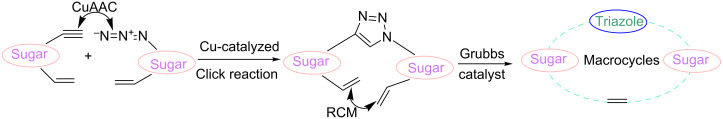
Build-couple-pair (B/C/P) strategy for macrocycles.

There are several DOS strategies to generate a collection of diverse molecules among them three-phase build-couple-pair (B/C/P) is one of the most frequently used. The B/C/P strategy involves build phase in which different building blocks were synthesized incorporating different diversity elements. These different building blocks were then combined together in the couple phase to give the substrates for the next phase. Finally, in the pair phase various functional-group-compatible reactions were used to generate distinct molecular scaffolds. The build-couple-pair strategy using iterative couple steps (B/C/C/P or B/C/C/C/P etc.) to increase the diversity of scaffolds accessed from the sets of building blocks has been exploited in recent times [59–63]. Also, simple and economical polyfunctional substrates available in abundance from the natural resources are ideal starting materials in DOS, which aims at providing quick access to libraries of diverse molecules. To exploit the strategy, it was envisioned that different sugars could serve as precursor for the necessary alkyne–alkene and azide–alkene functionalities and could be connected through a sequence of protection-deprotection-functionalization reactions at appropriate position (Figure 1). D-glucose, D-xylose and L-arabinose were used as the key starting materials for the DOS protocol. It was expected that each given sugar building block (generated in the building phase of the DOS) could be attached through Cu-catalyzed azide–alkyne cycloaddition (CuAAC) reaction (couple phase). Noteworthy, herein we utilized the CuAAC reaction as a medium for coupling different building blocks assembled iteratively to generate a 1,2,3-triazole moiety. This 1,2,3-triazole moiety linked as a spacer due to its inherent properties including stability towards acid–base hydrolysis, active participation in H-bonding, dipole–dipole and π-stacking interactions [37,64–66]. The reaction would then afford a range of acyclic precursors, which could then undergo the intramolecular cyclization reaction to furnish the macrocyclic compounds (pair phase). In the pair phase, CuAAC adducts were cyclized using a Ru-catalyzed metathesis reaction utilizing Grubbs second generation catalysts under greener reaction conditions (Figure 1).

### Build phase: preparation of building blocks

**Figure 2:**
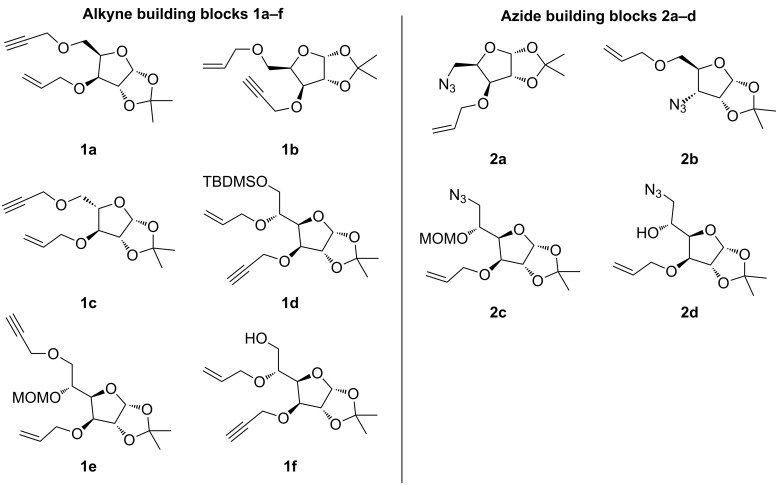
Different building blocks used for DOS.

The alkyne–alkene (**1a–f**) and azide–alkene (**2a–d**) building blocks were synthesized in multigram scale following known literature procedures (Figure 2). The experimental details of the various building blocks used for DOS can be found in Supporting Information File 1.

### Couple phase: Copper-catalyzed azide–alkyne cycloaddition (CuAAC)

**Scheme 1:**
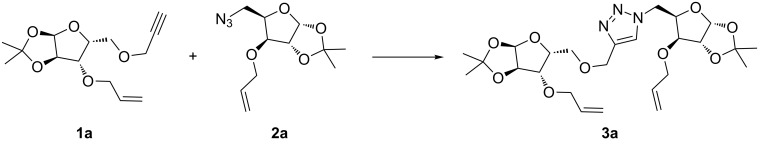
Cycloaddition reaction of alkyne-azide building block.

After having ready requisite building blocks our next goal was to assemble them iteratively to synthesize macrocyclic library (Scheme 1). All the reactions were monitored after an interval of 2 and 4 hours and if required than after 24 hours for the optimizations; the conversion in the reaction was calculated by comparing the ratio of integration of the terminal alkyne proton in the propargyl building block and the characteristic triazole–alkene proton in the cyclo-adducts. The click reaction proceeds under various conditions with a plenty of sources of Cu(I) [19]. We have selected copper iodide (CuI) as Cu(I) source for the CuAAC. Initially we tried the reaction using CuI as catalyst and DIPEA as a base for the cycloaddition of the alkyne (**1a**) azide and (**2a**) building blocks in acetonitrile at room temperature. Pleasingly, the reaction resulted in excellent conversion (by ^1^H NMR) in two hours with 70% isolated yield whereas addition of triethylamine in acetonitrile resulted in 65% yield. For developing greener conditions for the cycloaddition reaction, a control experiment with alkyne (**1a**) and azide (**2a**) in water at room temperature reacted up to 24 hours but in the absence of copper catalyst and base, only 6% conversion was observed (measured by ^1^H NMR; formation of two products were observed in the ratio of 77:23). Reaction in water at 70 °C under the above conditions gave a 33% conversion with a 63% selectivity for the product. Complete disappearance of starting materials after 24 hours with the formation of exclusively one product in 45% yield was observed when the reaction was performed at room temperature in water using 5 mol % CuI. Another reaction under similar conditions using CuI and DIPEA resulted in a lower yield of 35% after 24 hours. The next reaction was performed in water at 70 °C using 5 mol % catalysts in absence of a base. Interestingly, we observed complete disappearance of starting substrate in two hours with an excellent isolated yield of 95% for the exclusive product whereas addition of DIPEA under similar conditions resulted in the low yield of 48%. It is worth mentioning that the formation of the other regioisomer was not observed when the reactions were performed at 90 °C and 110 °C in water using 5 mol % CuI as catalyst. These results confirm the essential role of copper required for the high conversion and selectivity of the products (Table 1).

**Table 1:** Optimization of the reaction conditions for the cycloaddition.

Entry	Solvent	Base	Catalyst (CuI, mol %)	Temperature	Time (hours)	Yield %^a^

1	ACN	TEA	5	ambient	2	65
2	ACN	DIPEA	5	ambient	2	71
3	H_2_O	–	–	ambient	24	6^b^ (77% selectivity for **3a**)^c^
4	H_2_O	–	–	70 °C	24	33^b^ (63% selectivity for **3a**)^c^
5	H_2_O	–	5	ambient	24	45
6	H_2_O	DIPEA	5	ambient	24	35
**7**	**H_2_O**	**–**	**5**	**70 °C**	**2**	**95**
8	H_2_O	DIPEA	5	70 °C	2	48

^a^Isolated yield after column chromatography; ^b^conversion and ^c^product selectivity was measured by ^1^H NMR.

After screening various reaction conditions we found a “greener” protocol for the CuAAC reaction in water under mild heating and the use of base was eliminated. We have utilized this methodology for the synthesis of a range of cycloadducts (**3a–m**, Table 2) via iterative coupling of carbohydrate derived azide and propargyl building blocks to be used as metathesis substrates for the synthesis of novel sugar embedded macrocyclic molecules. Cycloaddition of xylose derived azide building blocks containing a primary azido group (**2a**) produced similar yields (i.e., **3a**, **3b** and **3d**) with xylose and arabinose derived building blocks containing a propargyl ether group on the primary OH group (**1a** and **1c**) or xylose derived building block containing a propargyl group on the secondary OH group (**1b**). Further, a comparatively lower yield for the cycloaddition reaction (**3c**) was obtained when both building blocks used contain a secondary azide group (**2b**) and a propargyl ether on the secondary OH group (**1c**). We have observed relatively low yields (**3e** and **3f**) when we used a combination of glucose (**1e** and **2c**) and xylose (**1b** and **2a**) derived building block whereas an excellent yield was obtained (**3g** and **3h**) when both coupling partners were derived from glucose (**1e**, **1d** and **2c**) irrespective of the position of the propargyl group on the primary OH (**1e**) or secondary OH group (**1d**). Next we thought of exploring the effect of protecting groups on the feasibility of the reaction and the yields and various building blocks with free OH groups were selected. It is worth mentioning that we did not observe any significant change in the reaction rate. Yields were relatively high (**3i, 3j, 3k** and **3l**) when we used combination of azide and propargyl building blocks containing at least one free OH group and generally yields were not influenced by the position of the azide or propargyl group onto building blocks. However, when both building blocks used for the cycloaddition containing a free OH group (**1f** and **2d**), the yield for the product was significantly low (**3m**). In conclusion, the CuAAC reaction of xylose derived building blocks gave relatively higher yields (**3a** and **3b**), except when both building blocks contain a secondary azide and a secondary propargyl ether group (**3c**). Cycloaddition of building blocks derived from glucose and xylose worked better with non-protected OH groups (**3i**, **3j** and **3k**) than with protected (**3e** and **3f**). Whereas glucose–glucose did work better with protected OH groups (**3g** and **3h**) than with non-protected (**3l** and **3m**).

**Table 2:** Copper catalyzed azide-alkyne cycloaddition.

Alkyne	Azide	Cycloadduct^a^	Yield %

**1a**	**2a**	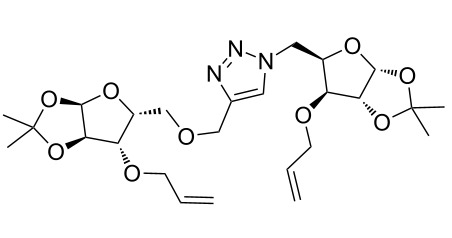 **3a**	95
**1b**	**2a**	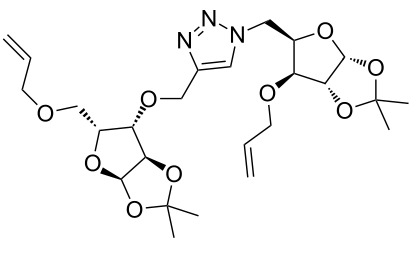 **3b**	94
**1b**	**2b**	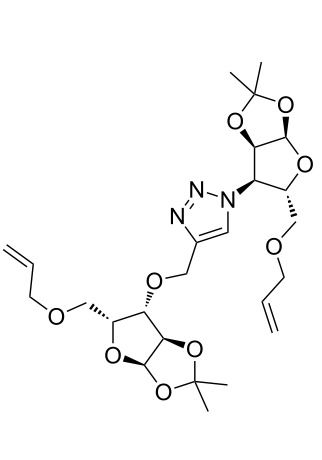 **3c**	75
**1c**	**2a**	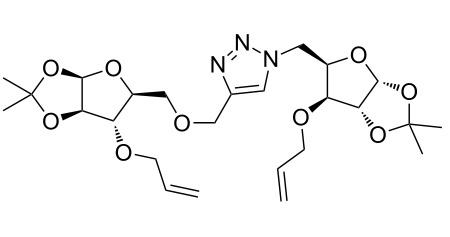 **3d**	90
**1e**	**2a**	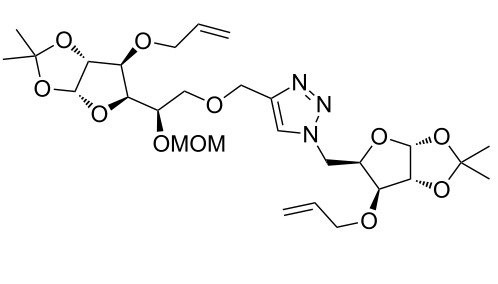 **3e**	76
**1b**	**2c**	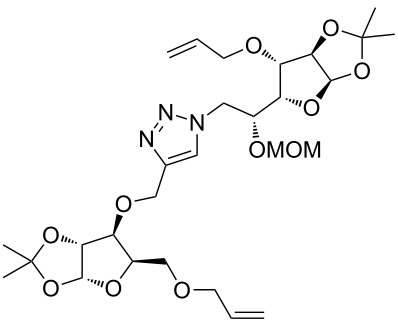 **3f**	78
**1e**	**2c**	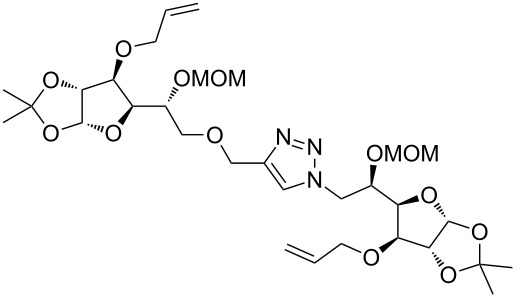 **3g**	92
**1d**	**2c**	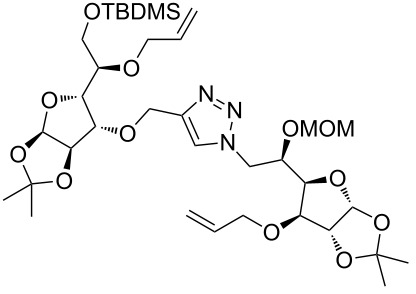 **3h**	91
**1b**	**2d**	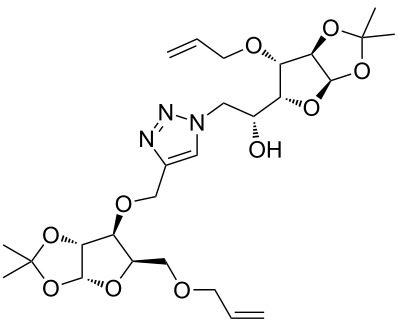 **3i**	85
**1a**	**2d**	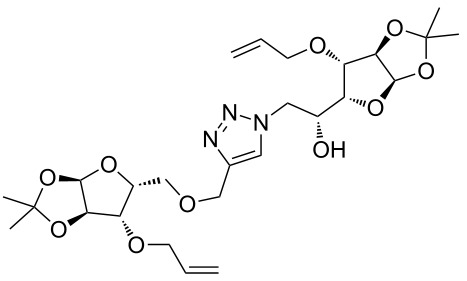 **3j**	87
**1f**	**2a**	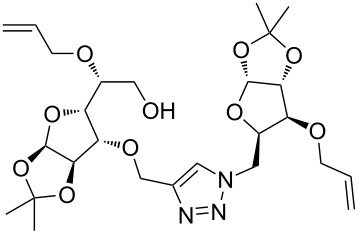 **3k**	95
**1e**	**2d**	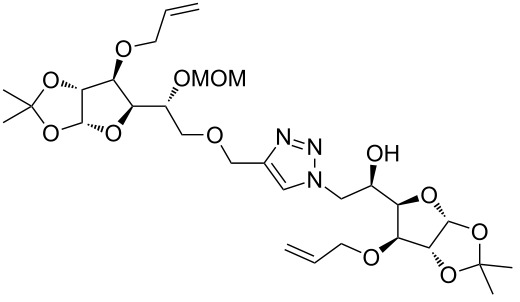 **3l**	77
**1f**	**2d**	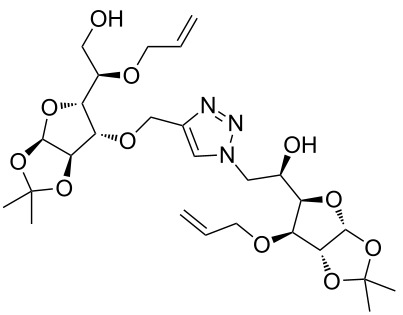 **3m**	67

^a^Method: CuI (5 mol %), water, 70 °C, 2 h.

### Pair phase: macrocyclization via Ru-catalyzed ring closing metathesis (RCM)

In the pair phase the range of linear substrates derived by CuAAC were cyclized via Ru-catalyzed ring closing metathesis reaction (Table 3). In general, RCM conditions used in this study proved to be very robust and delivered the macrocyclic product in moderate yields. To begin our RCM endeavor, we performed the macrocyclization reaction on cycloadduct **3a** in dichloromethane (10 mM) heating at 50 °C with 2 mol % second generation Grubbs catalyst. The reaction was incomplete after two hours and required an additional catalyst loading of 2 mol % and 1 mol % after every two hours. However, when we performed RCM reaction with 5 mol % catalyst under similar conditions, the reaction was completed in two hours with 61% isolated yield. The reaction was performed on the same substrate (i.e., **3a**) under high dilution (1 mM) with 5 mol % catalyst at 50 °C in dichloromethane and pleasingly we observed completion of the reaction in two hours with 63% isolated yield. Halogenated solvents are not preferred because of associated health and safety hazards and “greener” solvents for RCM reactions are always required. Ethyl acetate can be chosen as a “green, inexpensive and easily available reaction medium” for metathesis to synthesize this important yet synthetically challenging class of molecules [20]. Therefore, our next attention turns towards using ethyl acetate as “greener” solvent for the macrocyclization reaction. A reaction under high dilution (1 mM) with 5 mol % catalysts at 75 °C in ethyl acetate resulted in macrocycle **4a** in 84% yield (Table 3). The structure of **4a** was confirmed by ^1^H NMR based on the disappearance of the signal corresponding to the allyl group (from the starting material) and appearance of multiplet near δ 5.65 ppm for the alkenyl protons. Moreover, the complete structural assignment was done with the help of 2D NMR. It is worth mentioning here that the reaction proceeded with excellent selectivity for the *trans* product (confirmed by 2D NMR).

Next we performed the macrocyclization reaction with a range of metathesis precursors (**3b–m**) using dichloromethane and ethyl acetate solvents. Many of the RCM reactions were clean, however, to few the catalyst was added portion-wise until completion of the reaction judged by TLC analysis. The results are summarized in Table 3. Relatively better yields were observed in ethyl acetate compared to dichloromethane when the metathesis precursor consists of pentose (xylose and/or arabinose) building blocks irrespective of the position of the allyl group on the primary or secondary OH group (**4a**–**d**). However, yields were significantly lower in ethyl acetate when metathesis precursors were consisting of glucose with protected OH groups and xylose building blocks (**4e**, **4f**). Interestingly, metathesis substrate with both building blocks made-up of glucose with protected OH groups gave significantly better yield in ethyl acetate (**4g**, **4h**). Considerably low yields were observed in ethyl acetate when the metathesis substrate contains a free secondary OH group (**4i**, **4j**). Whereas yield was quite high in ethyl acetate when metathesis substrate contains a free primary OH group (**4k**). Metathesis yields were relatively higher in ethyl acetate when both glucose derived building blocks were used containing either one free OH group (**4l**) or two free OH groups (**4m**). Most notably RCM reactions in ethyl acetate produce almost the same or even better yields than in dichloromethane in most cases (apart from **4e**, **4f**, **4i**, **4j**) which confirms ethyl acetate as a viable, greener, inexpensive and easily available alternative to the highly hazardous chlorinated solvent which is a traditionally and most frequently used solvent for RCM reactions.

**Table 3:** Application of ring-closing metathesis reactions in the synthesis of macrocycles.

Substrate	Method^a^ (mol %; time; yield)	RCM product

**3a**	A (5; 2 h; 63%) B (5; 2 h; 84%)	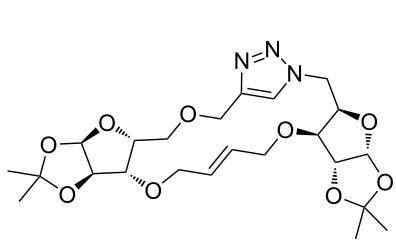 **4a**
**3b**	A (5; 2 h; 85%) B (5; 2 h; 94%)	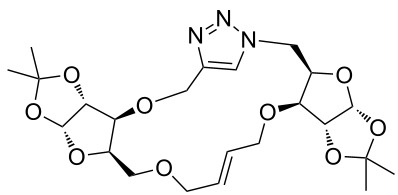 **4b**
**3c**	A (5; 2 h; 88%) B (5; 3 h; 94%)	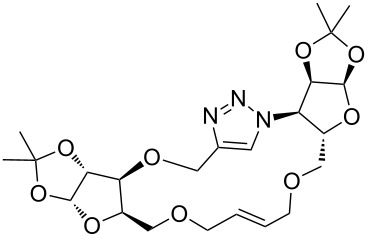 **4c**
**3d**	A (5; 2 h; 70%) B (5; 2 h; 90%)	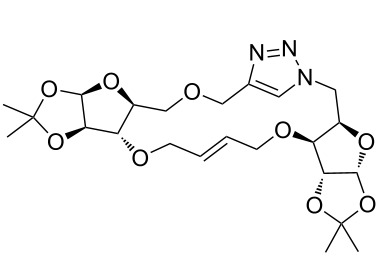 **4d**
**3e**	A (5+5; 3 h; 88%) B (5+3; 3 h; 40%)	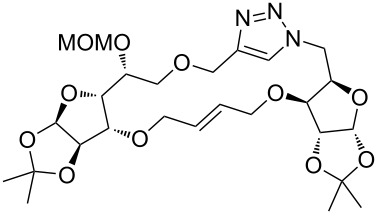 **4e**
**3f**	A (5+3; 3 h; 83%) B (5; 2 h; 39%)	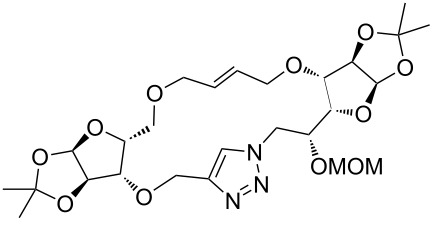 **4f**
**3g**	A (5; 2 h; 77%) B (5+3; 3 h; 92%)	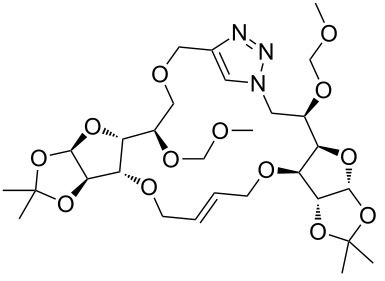 **4g**
**3h**	A (5+3; 3 h; 82%) B (5; 2 h; 92%)	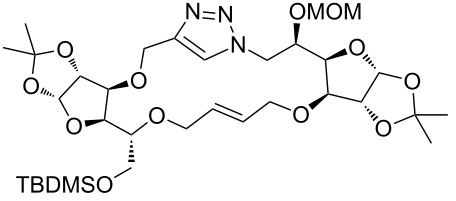 **4h**
**3i**	A (5; 2 h; 95%) B (5; 2 h; 19%)	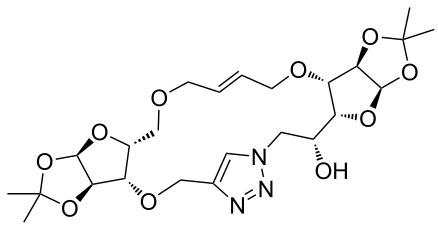 **4i**
**3j**	A (5; 2 h; 84%) B (5; 2 h; 56%)	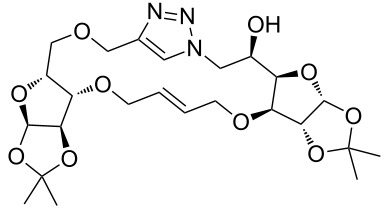 **4j**
**3k**	A (5; 2 h; 81%) B (5; 2 h; 96%)	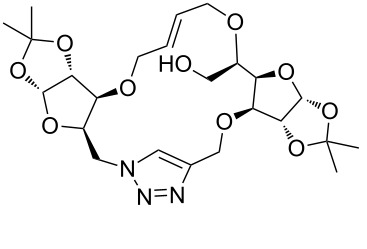 **4k**
**3l**	A (5; 2 h; 53%) B (5; 2 h; 61%)	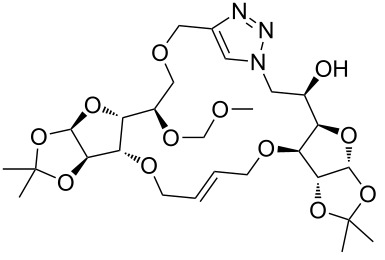 **4l**
**3m**	A (5; 2 h; 40%) B (5; 2 h; 55%)	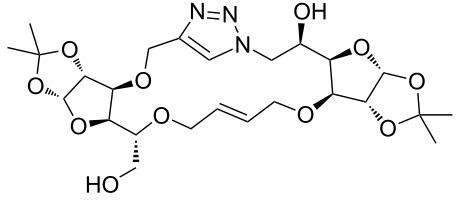 **4m**

^a^Methods: A: Grubbs second-generation catalyst, CH_2_Cl_2_, 50 °C; B: Grubbs second-generation catalyst, ethyl acetate, 75 °C.

To check the effect of purity of the cycloadduct on the rate and feasibility of subsequent RCM reactions and on isolated yield obtained in the individual steps, we explored the feasibility of the RCM reaction without isolating the product at the couple phase. Compounds **4e**–**h** were synthesized without purifying the respective cycloaddition products. The second generation Grubbs catalyst catalyzed RCM reaction was performed using the crude substrate in ethyl acetate at 75 °C (Table 4). Interestingly, isolated yields for **4e, 4f** and **4h** were comparable to the yields obtained when they were synthesized in two separate steps. However, the yield obtained in case of **4g** was significantly lower in case of the direct reaction compared to when the compound was synthesized via the two-step process (Table 4).

**Table 4:** Feasibility studies of cycloaddition and RCM reaction in single and two-step protocol.

RCM product		Two-step protocol^a^					Direct protocol^a^	
				
		CuAAC Yield (%)	Grubbs cat. (mol %)	RCM yield (%)	Combined yield (%)		Grubbs cat. (mol %)	Yield (%)
**4e**		76	(5+3)	40	31		(5+5)	32
**4f**		78	5	39	30		5	29
**4g**		92	(5+3)	92	85		5	49
**4h**		91	5	92	84		5	80

^a^Isolated yield after column chromatography.

**Scheme 2:**
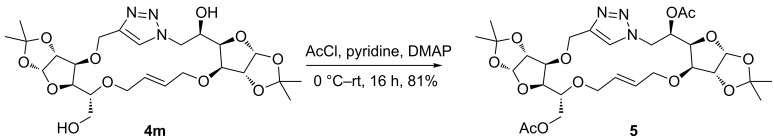
Acetylation of macrocycle **4m**.

Lastly, the macrocycle **4m** was acetylated in pyridine using acetyl chloride and a catalytic amount of DMAP to furnish diacetate **5**. The ^1^H NMR analysis of **5** clearly showed presence of two singlets at δ 2.08 and 2.06 ppm integrating for three protons each corresponding to acetate methyl groups. Acetate groups were further confirmed by ^13^C NMR wherein signals corresponding to two carbonyl groups apparent at δ 170.9, 170.5 ppm and two methyl groups at δ 21.0 and 20.9 ppm. The product was further confirmed by mass spectrometry (Scheme 2).

## Conclusion

In conclusion we report a novel and green route to synthesize sugar embedded macrocycles (in total we have synthesized 13 macrocycles with 17 to 19-membered rings) which involves CuAAC reaction and Ru-catalyzed RCM reaction. The CuAAC reaction were performed in water and produce moderate yields. Thus, we have successfully demonstrated novel application of build-couple-pair (B/C/P) strategy in DOS and synthesized 13 new macrocycles (**4a–m**). This synthetic method represents a significant advantage over current routes for sugar embedded macrocycles where reactions are rapid, eco-friendly without compromise in yield and selectivity.

## Supporting Information

File 1 Experimental details and analytical data.
