# Supplementary material for: An eco-compatible strategy for the diversity-oriented synthesis of macrocycles exploiting carbohydrate-derived building blocks
Source: Beilstein J Org Chem. 2017 Jun 9;13:1106–18. doi: 10.3762/bjoc.13.110 (PMC5480360; doi:10.3762/bjoc.13.110)
Supplement: File 1 — Experimental details and analytical data. [file Beilstein_J_Org_Chem-13-1106-s001.pdf]

Supporting Information

for

An eco-compatible strategy for the diversity-oriented synthesis of macrocycles exploiting carbohydrate-derived building blocks

Sushil K. Maurya[*,§,1,2] and Rohit Rana[1,2]

Address: [1]Natural Product Chemistry and Process Development Division, CSIR- Institute of Himalayan Bioresource Technology, Palampur, Himachal Pradesh, 176 061, India and [2]Academy of Scientific and Innovative Research, CSIR- Institute of Himalayan Bioresource Technology, Palampur, Himachal Pradesh, 176 061, India.

Email: Sushil K. Maurya - skmaurya@ihbt.res.in,

[§]Phone: +91-1894-230742

Experimental details and analytical data.

Content

Experimental S2

General information S2

Synthesis of building blocks S3–S34

Synthesis of cycloadducts S35–S42

Synthesis of RCM products S43–S52

References S53–S54

NMR spectra S55–S96

Experimental

General information

The ^{1}H and ^{13}C NMR spectra were recorded on Bruker-Avance 600 and 151 MHz instrument, respectively using TMS as internal standard. The chemical shift values are on δ scale and the coupling constants (*J*) are in Hz. Mass spectra were recorded either on Water Q-TOF mass spectrometer or on Agilent 6560 ion mobility Q-TOF LC/MS. All reactions were monitored by thin-layer chromatography (TLC) using pre-coated silica plates (Merck F_{254}, 0.25 mm thickness) and the spots were detected either under UV light, I_2 and by charring with *p*-anisaldehyde in glacial acetic acid and sulfuric acid. Silica gel (60–120, 100–200, 250–400 mesh, S. D. Fine make) was used for column chromatography and a gradient elution using n-hexane and ethyl acetate or methanol and dichloromethane was performed based on Merck F_{254} aluminium TLC sheets. Unless and otherwise noted, all commercially available starting materials and reagents were used without further purification. Unless otherwise noted, all solvents for routine isolation of products and chromatography were reagent grade and used without further purification. All evaporations were carried out under reduced pressure using Büchi rotary evaporator below 50 °C. Glassware were dried in oven at 100 °C for 12 hours. Air and moisture sensitive reactions were performed under an argon/ nitrogen atmosphere.

Synthesis of building blocks

Synthesis of xylose derived building blocks

6-Allyloxy-2,2-dimethyl-5-(prop-2-ynyloxymethyl)tetrahydrofuro[2,3-*d*][1,3]dioxole (1a):

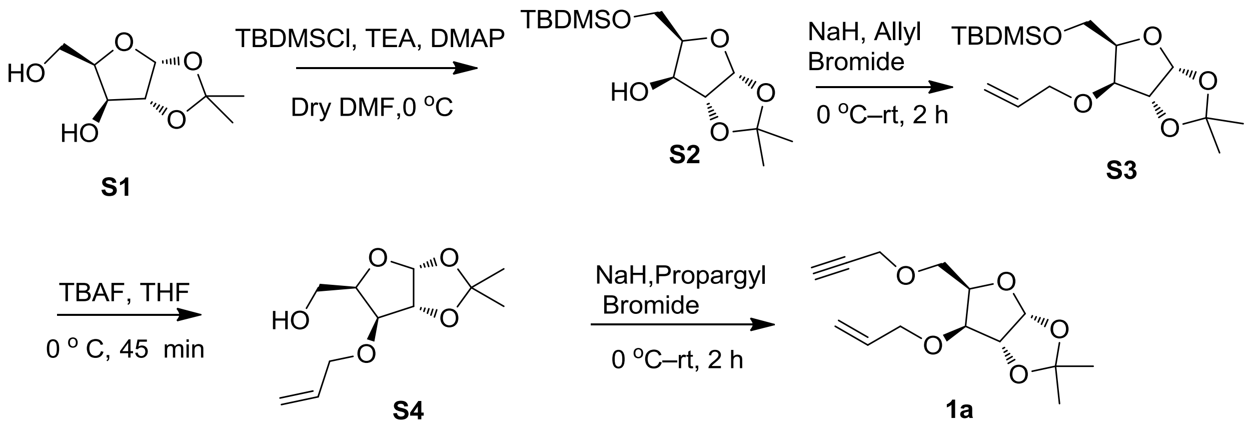

5-(Hydroxymethyl)-2,2-dimethyltetrahydrofuro[2,3-*d*][1,3]dioxol-6-ol (S1)

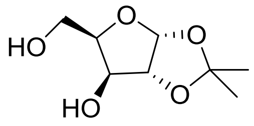

S1

S1 was prepared following literature method [SR1], [SR2].

To a solution of D-xylose (32.5 g, 0.22 mol) in acetone (780 mL) was added conc. H_2SO_4 (28.6 mL, 0.66 M in acetone) at 0 °C and the mixture was stirred at room temperature for 30 hours. A solution of Na_2CO_3 (39.0 g, 0.37 mol) in water (340 mL) was added to neutralize (pH = 6–7) the reaction mixture and stirred for a further 2.5 h. The mixture was filtered and the residue was washed with acetone. The solvent was removed completely from the filtrate under reduced pressure and the resulting residue was purified by column chromatography to give the desired product **S1** (39.3 g, 95%) as pale yellow syrup.

$\mathbf{R}_{\boldsymbol{f}}$ 0.50 (5:95, methanol:dichloromethane).

5-(((*tert*-Butyldimethylsilyl)oxy)methyl)-2,2-dimethyltetrahydrofuro[2,3-*d*][1,3]dioxol-6-ol (S2)

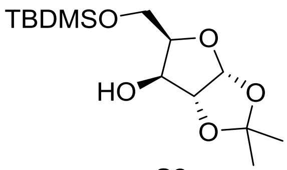

S2

S2 was prepared following literature method [SR3]

To a solution of **S1** (15.5 g, 81.58 mmol) in anhydrous DMF (50 mL) at 0 °C was added Triethyl amine (20.60 g, 203.95 mmol). The resulting mixture was stirred at 0 °C for 10 min and was added TBDMSCl (13.46 g, 89.74 mmol) and catalytic amount of DMAP (0.99 g, 8.16 mmol). The resulting mixture was stirred at 0 °C for 15 min. A white precipitate was formed which was filtered off, filtrate was diluted with water (100 mL) and extracted in ethyl acetate (3 × 200 mL). Pooled organic layers are washed with brine (200 mL), dried over Na_2SO_4 and solvent was removed under reduced pressure to obtain the crude product which was purified by silica gel column chromatography to afford **S2** as colourless oil (22.0 g, 88%).

R*f* 0.60 (20:80, ethyl acetate:hexane).

^{1}H NMR (600 MHz, $CDCl_3$): δ 5.95 (d, *J* = 3.6 Hz, 1H), 4.49 (d, *J* = 3.6 Hz, 1H), 4.37 (s, 1H), 4.32 (s, 1H), 4.15 – 4.10 (m, 3H), 1.47 (s, 3H, methyl), 1.31 (s, 3H, methyl), 0.88 (s, 9H, silyl), 0.10 (d, *J* = 2.9 Hz, 6H, silyl).

^{13}C NMR (151 MHz, $CDCl_3$): δ 111.6, 105.1 (anomeric), 85.7, 78.2, 62.5, 26.9 (methyl), 26.2 (methyl), 25.8 (silyl), 18.2 (silyl), -5.3 (silyl), -5.5 (silyl).

(6-(Allyloxy)-2,2-dimethyltetrahydrofuro[2,3-*d*][1,3]dioxol-5-yl)methoxy(*tert*-butyl) dimethylsilane (S3)

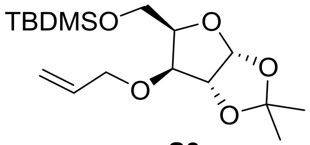

S3

To a solution of **S2** (7 g, 22.99 mmol) in DMF (35 mL) was added sodium hydride (1.11 g, 45.98 mmol) at 0 °C and stirred for 10 min. Allyl bromide (3.34 mL, 27.59 mmol) was added and reaction mixture stirred at to room temperature for 2 h. After completion (TLC), reaction mixture was diluted with water (60 mL) and extracted in ethyl acetate (3 × 120 mL), pooled organic layers were washed with brine (120 mL), dried over Na_2SO_4 and concentrated *in vacuo* and residue obtained was purified by silica gel column chromatography to afford the product **S3** as colorless oil (7.6 g, 96.20%).

$\mathbf{R}_f$ 0.58 (10:90, ethyl acetate:hexane).

^{1}H NMR (600 MHz, $CDCl_3$): δ 5.88 – 5.81 (m, 1H, allyl-H), 5.25 (dd, *J* = 17.2, 1.6 Hz, 1H, alkene t-H), 5.15 (dd, *J* = 10.4, 1.2 Hz, 1H, alkene c-H), 4.51 (d, *J* = 3.7 Hz, 1H), 4.21 – 4.18 (m, 1H), 4.10 (tdd, *J* = 12.8, 5.5, 1.2 Hz, 1H), 4.01 (tdd, *J*=12.8 ,5.5 ,1.2 Hz, 1H), 3.88 (d, *J* = 3.1, 1H), 3.83 (dd, *J* =9.9, 7.9 Hz, 1H), 3.78 (dd, *J* =9.9, 5.2 Hz, 1H), 1.47 (s, 3H, methyl), 1.28 (s, 3H, methyl), 0.86 (s, 9H, silyl), 0.04 (d, *J* = 2.9 Hz, 6H, silyl).

^{13}C NMR (151 MHz, $CDCl_3$): δ 134.2 (alkene), 117.2 (alkene), 111.5, 104.9 (anomeric), 82.6, 81.0, 80.7, 71.2, 59.8, 26.8 (methyl), 26.2 (methyl), 25.8 (silyl), 18.1 (silyl), -5.3 (silyl), -5.5 (silyl).

HRMS (TOF, MS, ES+): m/z calcd. for $C_{17}H_{33}O_5Si$: 345.2092; found: 345.2098 ($M+H^+$).

6-(Allyloxy)-2,2-dimethyltetrahydrofuro[2,3-*d*][1,3]dioxol-5-yl)methanol (S4)

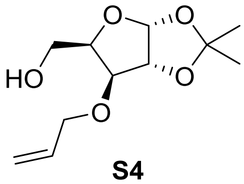

S4

S4 was prepared following literature method [SR4]

1 M Tetrabutylammonium fluoride (TBAF) (6.36 mL, 24.3 mmol) was added slowly to a solution of S3 (7.6 g, 22.09 mmol) in THF (38 mL) at 0 °C and stirred at room temperature for 45 min. The solvent was removed and the residue was taken up in ethyl acetate (100 mL), washed with water (50 mL), brine (50 mL), and dried over Na_2SO_4. The solvent was removed under reduced pressure and crude was purified by silica gel column chromatography to afford the product **S4** as colorless oil (3.6 g, 70.9%).

R_f 0.55 (40:60, ethyl acetate:hexane).

^{1}H NMR (600 MHz, CDCl$_3$): δ 5.96 (d, *J* =3.72 Hz, 1H), 5.89 – 5.82 (m, 1H, allyl-H), 5.29 (dd, *J* =17.2, 1.3 Hz, 1H, alkene t-H), 5.22 (d, *J* =10.3 Hz, 1H, alkene c-H), 4.57 (d, *J* =3.8 Hz, 1H), 4.28 (q, 4.4 Hz, 1H), 4.15 (dd, *J* = 12.8, 5.1 Hz, 1H), 3.99 – 3.93 (m, 3H), 3.88 – 3.86 (m, 1H), 1.48 (s, 3H, methyl), 1.30 (s, 3H, methyl).

^{13}C NMR (151 MHz, CDCl$_3$): δ 133.5 (alkene), 118.0 (alkene), 111.7, 105.0 (anomeric), 83.0, 82.5, 79.9, 70.0, 61.0, 26.8 (methyl), 26.3 (methyl).

(3a*R*,5*R*,6*S*,6a*R*)-2,2-Dimethyl-6-(prop-2-en-1-yloxy)-5-[(prop-2-yn-1-yloxy)methyl]-tetrahydro-2*H*-furo[2,3-*d*][1,3]dioxole (1a)

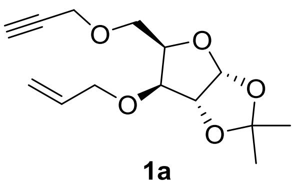

1a

Further, to a solution of **S4** (1.5 g, 6.52 mmol) in DMF (7.5 mL) was added sodium hydride (313 mg, 13.04 mmol) at 0 °C and stirred for 10 min at the same temperature. Propargyl bromide (1 mL, 7.17 mmol) was added and reaction mixture was warmed to room temperature and stirred at room temperature until completion (TLC). The reaction mixture was diluted with water (30 mL) and extracted in ethyl acetate (3 × 60 mL). The pooled organic layers were washed with brine (60 mL), dried over Na_2SO_4 and solvent was removed under reduced pressure to give the crude which was purified by passing through a column of silica to give **1a** as yellowish syrup (1.48 g, 85%)**.**

R*f* 0.59 (20:80, ethyl acetate:hexane).

^{1}H NMR (600 MHz, CDCl$_3$): δ 5.91 (d, *J* = 3.67 Hz, 1H), 5.90 – 5.83 (m, 1H, allyl), 5.28 (td, *J* = 17.1, 1.6 Hz, 1H), 5.19 (td , *J* = 10.5, 1.6 Hz, 1H), 4.54 (d, *J* = 3.7 Hz, 1H), 4.36 (m, 1H), 4.24 (ddd, *J* = 15.8, 2.3, 1.2 Hz, 1H), 4.16 (ddd, *J* = 15.8, 2.3, 1.2 Hz, 1H), 4.12 (ddd, *J* = 13.0, 5.9, 1.4 Hz, 1H), 4.00 (ddd, *J* = 13.0, 5. 9, 1.4 Hz, 1H), 3.90 (d, *J* = 3.0 Hz, 1H), 3.82 (ddd, *J* = 9.9, 5.7, 1.2 Hz, 1H), 3.72 (ddd, *J* = 9.9, 5.7, 1.2 Hz, 1H), 2.43 (m, 1H, alkyne-H), 1.48 (s, 3H, methyl), 1.30 (s, 3H, methyl).

^{13}C NMR (151 MHz, CDCl$_3$): δ 134.0 (alkene), 117.6 (alkene), 111.7, 105.1 (anomeric), 82.4, 81.6, 79.5 (alkyne), 79.0, 74.7, 71.1, 67.3, 58.6, 26.8 (methyl), 26.3 (methyl).

HRMS (TOF, MS, ES+): m/z calcd. for $C_{14}H_{20}NaO_5$: 291.1208, Found: 291.1196

5-Allyloxymethyl-2,2-dimethyl-6-(prop-2-ynyloxy)tetrahydrofuro[2,3-*d*][1,3]dioxole (1b):

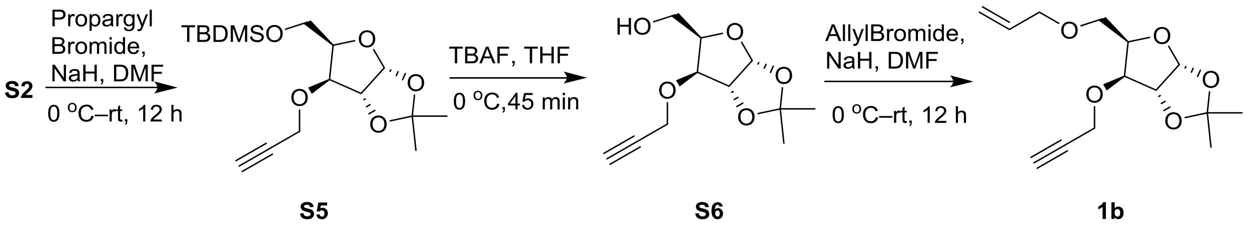

***tert*-Butyl-((2,2-dimethyl-6-(prop-2-yn-1-yloxy)tetrahydrofuro[2,3-*d*][1,3]dioxol-5-yl)methoxy)dimethylsilane (S5)**

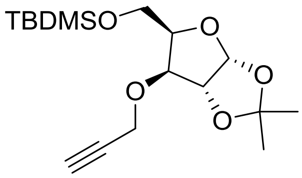

S5

S5 was prepared following literature method [SR5].

To a solution of **S2** (5 g, 16.45 mmol) in DMF (25 mL) was added sodium hydride (0.789 g, 32.9 mmol) at 0 °C and stirred for 10 min. Propargyl bromide (1.56 mL, 18.09 mmol) was added and reaction mixture stirred at to room temperature for 12 h. After completion (TLC), reaction mixture was diluted with water (30 mL) and extracted in ethyl acetate (3 × 60 mL), pooled organic layers were washed with brine (60 mL), dried over Na_2SO_4 and concentrated *in vacuo* to give the crude product **S5** which was purified by silica gel column chromatography to give **S5** as colorless oil (4.1g, 73%)**.**

R*f* 0.40 (10:90, ethyl acetate:hexane).

[(3a*R*,5*R*,6*S*,6a*R*)-2,2-Dimethyl-6-(prop-2-yn-1-yloxy)-tetrahydro-2H-furo[2,3-d][1,3]dioxol-5-yl]methanol (S6)

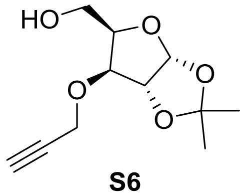

S6

1 M Tetrabutylammonium fluoride (TBAF) (12.3 mL, 12.3 mmol) was added slowly to a solution of **S5** (3.83 g, 11.2 mmol) in THF (19.5 mL) at 0 °C and stirred at for 45 min. The solvent was removed and the residue was taken up in ethyl acetate (100 mL), washed with water (50 mL), brine (50 mL), and dried over Na_2SO_4. The solvent was removed under reduced pressure and the crude product was purified by silica gel column chromatography to afford the desired product **S6** as colorless oil (1.91 g, 74%).

R_f 0.57 (40:60, ethyl acetate:hexane); **^{1}H NMR (600 MHz, $CDCl_3$):** δ 5.92 (d, *J* = 3.8 Hz, 1H), 4.59 (d, *J* = 3.8 Hz, 1H), 4.30 (ddd, *J* = 8.9, 5.1, 3.5 Hz, 1H), 4.26 (dd, *J* = 16.1, 2.4 Hz, 1H), 4.18 (dd, *J* = 16.1, 2.4 Hz, 1H), 4.15 (d, *J* = 3.4 Hz, 1H), 3.88 (dd, *J* = 11.9, 5.8 Hz, 1H), 3.82 (dd, *J* = 11.9, 5.8 Hz, 1H), 2.49 (t, *J* = 2.4 Hz, 1H, alkyne-H), 1.47 (s, 3H, methyl), 1.30 (s, 3H, methyl).

^{13}C NMR (151 MHz, $CDCl_3$): δ 111.8, 105.0 (anomeric), 82.3, 82.0, 80.1, 78.9 (alkyne), 55.4, 60.5, 57.4, 26.7 (methyl), 26.2 (methyl).

(3a*R*,5*R*,6*S*,6a*R*)-2,2-dimethyl-5-[(prop-2-en-1-yloxy)methyl]-6-(prop-2-yn-1-yloxy)-tetrahydro-2H-furo[2,3-d][1,3]dioxole 1b

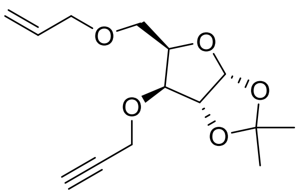

1b

Further, to a solution of **S6** (0.9 g, 3.95 mmol) in DMF (4.5 mL) was added sodium hydride (0.189 g, 7.9 mmol) at 0 ^{o}C and stirred for 10 min. Allyl bromide (0.409 mL, 4.74 mmol) was added and stirred at room temperature for 12 h. After completion (TLC), reaction mixture was diluted with water (50 mL) and extracted in ethyl acetate (3 × 60 mL), pooled organic layers were washed with brine (50 mL). Organic phase was dried over Na_2SO_4 and solvent was removed under reduced pressure to give the crude which was purified by silica gel column chromatography to furnish **1b** (0.867 g, 82%).

R*f* 0.60 (20:80, ethyl acetate:hexane).

^{1}H NMR (600 MHz, $CDCl_3$): δ 5.93– 5.86 (m, 1H, allyl-H), 5.89 (d, *J* = 3.8 Hz, 1H), 5.27 (dd, *J* = 17.3, 1.3 Hz, 1H), 5.17 (d, *J* = 10.4 Hz, 1H), 4.59 (d, *J* = 3.8 Hz, 1H), 4.38 (td, *J* = 6.1, 3.1 Hz, 1H), 4.21 (dd, *J* = 16.0, 2.3 Hz, 2H), 4.09 (d, *J* = 3.1 Hz, 1H), 4.06 (dd, *J* = 12.4, 5.63 Hz, 1H), 4.01 (dd, *J* = 12.9, 5.6 Hz, 1H), 3.68 (dd, *J* = 10.1, 5.9 Hz, 1H), 3.63 (dd, *J* = 10.2, 6.3 Hz, 1H), 2.45 (t, *J* = 2.4 Hz, 1H, alkyne-H), 1.49 (s, 3H, methyl), 1.30 (s, 3H, methyl).

^{13}CNMR (151 MHz, $CDCl_3$): δ 134.5 (alkene), 117.2 (alkene), 111.8, 105.0 (anomeric), 82.3, 81.5, 79.1 (alkyne), 79.0, 75.0 (alkyne), 72.4, 67.7, 57.6, 26.8 (methyl), 26.3 (methyl).

HRMS (TOF, MS, ES+): m/z calcd. for $C_{14}H_{20}NaO_5$: 291.1208; Found: 291.1198 [M+Na].

6-Allyloxy-5-azidomethyl-2,2-dimethyl-tetrahydro-furo[2,3-*d*][1,3]dioxole (2a):

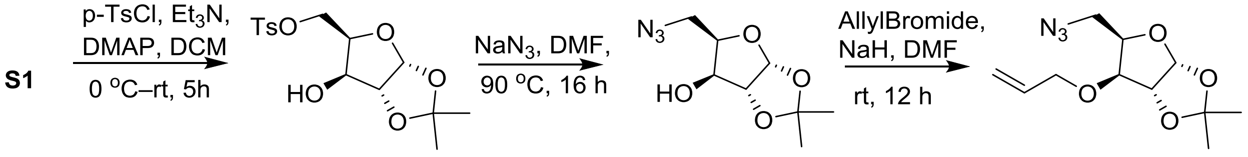

S7 **S8** **2a**

6-Hydroxy-2,2-dimethyltetrahydrofuro[2,3-*d*][1,3]dioxol-5-yl-methyl-4-methylbenzene sulfonate S7

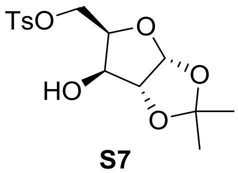

S7

S7 was prepared following literature method [SR6]

To a solution of **S1** (2.4 g, 12.62 mmol) in dichloromethane (24 mL) at 0 °C was added triethyl amine (3.5 mL, 25.24 mmol) and stirred for 10 min. then, *p*-toluenesulfonyl chloride (2.89 g, 15.14 mmol) and DMAP (0.77 g, 6.31 mmol) was added and stirred at room temperature for 5 hours. After completion (TLC), reaction was quenched with 1 N HCl and extracted the product in ethyl acetate (3 × 100 mL). Pooled organic layers were dried over Na_2SO_4, solvent was removed under reduced pressure and crude **S7** (4 g, 92%) was used for the next step without further purification.

R*f* 0.50 (40:60, ethyl acetate:hexane).

5-(Azidomethyl)-2,2-dimethyltetrahydrofuro[2,3-*d*][1,3]dioxol-6-ol (S8)

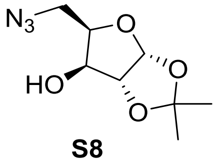

S8

S8 was prepared following literature method [SR6]

To a solution of crude **S7** (1.0 g, 2.90 mmol) dissolved in DMF (5 mL) was added NaN_3 (0.5 g, 7.25 mmol) and solution was heated at 90 ^oC for 16 hours. The reaction mixture was diluted with water (50 mL) and extracted in ethyl acetate (3 × 50 mL). Pooled organic layers were washed with brine (50 mL), dried over Na_2SO_4 and concentrated *in vacuo* and residue obtained was purified by silica gel column chromatography to furnish **S8** (0.5 g, 80%).

$\mathbf{R}_f$ 0.50 (30:70, ethyl acetate:hexane).

6-Allyloxy-5-azidomethyl-2,2-dimethyltetrahydrofuro[2,3-*d*][1,3]dioxole (2a):

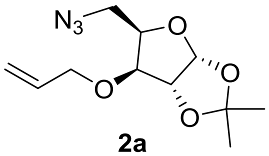

2a

To a solution of **S8** (0.20 g, 0.93 mmol) in DMF (1 mL) was added sodium hydride (0.045 g 1.8 mmol) and allyl bromide (0.096 mL, 1.1 mmol) at 0 °C and stirred at room temperature for 12 hours. After completion (TLC), the reaction mixture was diluted with water (50 mL) and extracted in ethyl acetate (3 × 50 mL). Pooled organic layers were washed with brine (50 mL), dried over Na_2SO_4 and concentrated *in vacuo* to give the residue which was further purified by silica gel column chromatography to furnish **2a** as syrup (0.215 g, 91%).

R_f 0.50 (20:80, ethyl acetate:hexane).

^{1}H NMR (600 MHz, CDCl$_3$): δ 5.90 (d, *J* = 3.3 Hz, 1H), 5.90 – 5.83 (m, 1H, allyl-H), 5.29 (d, *J* = 17.2 Hz, 1H), 5.21 (d, *J* = 10.3 Hz, 1H), 4.56 (d, *J* = 3.0 Hz, 1H), 4.29 (m, 1H), 4.14 (dd, *J* = 12.7, 4.9 Hz, 1H), 3.98 (dd, *J* = 12.7, 5.5 Hz, 1H), 3.92 (s, 1H), 3.59 – 3.51 (m, 2H), 1.48 (s, 3H, methyl), 1.30 (s, 3H, methyl).

^{13}C NMR (151 MHz, CDCl$_3$): δ 133.7 (alkene), 117.9 (alkene), 111.9, 105.0 (anomeric), 82.1, 81.4, 78.7, 70.9, 49.1, 26.8 (methyl), 26.3 (methyl).

HRMS (TOF, MS, ES+): m/z calcd. for $C_{11}H_{17}N_3NaO_4$: 278.1117; Found: 278.1102 [M+Na].

Synthesis of glucose-derived building blocks:

6-Allyloxy-5-(1-methoxymethoxy-2-(prop-2-ynyloxy)ethyl)-2,2-dimethyltetrahydrofuro[2,3-*d*][1,3]dioxole (1e):

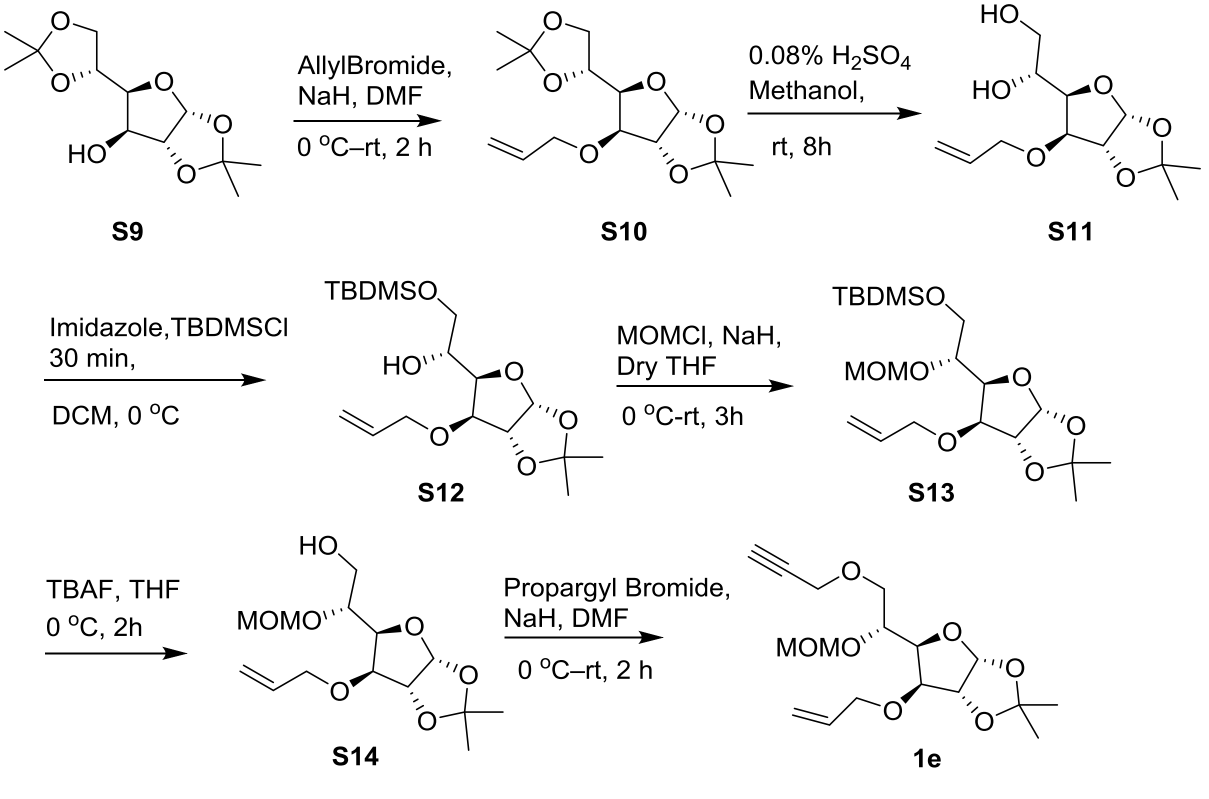

5-((*R*)-2,2-Dimethyl-1,3-dioxolan-4-yl)-2,2-dimethyltetrahydrofuro[2,3-*d*][1,3]dioxol-6-ol (S9)

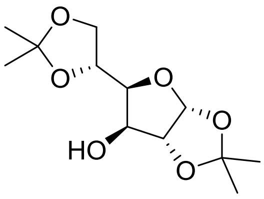

S9

S9 was prepared following literature method [SR2], [SR7].

D-Glucose (30 g, 166.5 mmol) was added to dry acetone (600 mL) at room temperature and was followed by anhydrous $CuSO_4$ (30 g, 610.60 mmol). The reaction mixture was cooled to 0 °C, and conc. H_2SO_4 (22 mL, 0.66 molar in acetone) was added drop-wise over a period of 10 min. The reaction mixture was stirred at room temperature for 30 h. After completion, (TLC) reaction mixture was neutralized with a saturated solution of K_2CO_3. The solution was filtered and the filtrate was evaporated under reduced pressure. The residue thus obtained was extracted with chloroform and dried over anhydrous Na_2SO_4, concentrated under reduced pressure to afford a yellowish solid, which was recrystallized from chloroform: hexane (1:9) to to afford the product glucose-di-acetone (GDA) **S9** (24 g, 55%) as white crystals**.**

R*f* 0.58 (60:40, ethyl acetate:hexane).

6-(Allyloxy)-5-((*R*)-2,2-dimethyl-1,3-dioxolan-4-yl)-2,2-dimethyltetrahydrofuro[2,3-*d*][1,3] dioxole (S10)

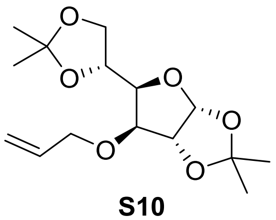

S10

S10 was prepared following literature method [SR8]

To a solution of the **S9** (6 g, 23.08 mmol) in DMF (30 mL) was added sodium hydride (0.96 g, 46.15 mmol) at 0 °C, and allyl bromide (2.9 mL,27.69 mmol) and stirred at room temperature for 12 hours. After completion (TLC), the reaction mixture was diluted with water (30 mL) and extracted in ethyl acetate (3 × 60 mL). Pooled organic layers were washed with brine, dried over Na_2SO_4 and solvent was removed under reduced pressure and residue was purified by silica gel column chromatography to afford **S10** (6.75 g, 97%) as colourless oil.

$\mathbf{R}_f$ 0.58 (20:80, ethyl acetate:hexane).

^{1}H NMR (600 MHz, $CDCl_3$): δ 5.90 – 5.83 (m, 2H), 5.28 (d, *J* =17.2 Hz, 1H, alkene t-H), 5.18 (d, *J* =10.5 Hz, 1H, alkene c-H), 4.52 (d, *J* =3.6 Hz, 1H), 4.29 (q, *J* =6.2 Hz, 1H), 4.14 – 4.06 (m, 4H), 3.97 (dd, *J* =8.4, 5.9 Hz, 1H), 3.92 (d, *J* =2.8 Hz, 1H), 1.47 (s, 3H, methyl), 1.40 (s, 3H, methyl), 1.33 (s, 3H, methyl), 1.29 (s, 3H, methyl).

6-(Allyloxy)-2,2-dimethyltetrahydrofuro[2,3-d][1,3]dioxol-5-yl)ethane-1,2-diol (S11)

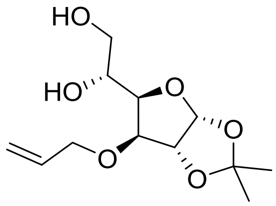

S11

S11 was prepared following literature method [SR8]

To a solution of S14 (6 g, 22.09 mmol) in methanol (60 mL) at 0 °C was added conc.H_2SO_4 (3.43 mL, 24.30 mmol). The resulting mixture was stirred at room temperature for 8 hours. After completion (TLC), solvent was removed and the residue was taken up in DCM and dried over Na_2SO_4, concentrated under reduced pressure and purified by column chromatography to afford **S11** (4.9 g, 94%) as colourless oil.

R_f 0.45 (70:30, ethyl acetate:hexane).

^{1}H NMR (600 MHz, $CDCl_3$): δ 5.90 – 5.84 (m, 2H), 5.28 (d, *J* =16.8 Hz, 1H, alkene t-H), 5.19 (d, *J* =10.4 Hz, 1H, alkene c-H), 4.53 (d, *J* =3.6 Hz, 1H), 4.14 (dd, *J* =12.3, 5.0 Hz, 1H), 4.08 (dd, *J* =8.0, 3.2 Hz, 1H), 4.04 (dd, *J* =12.7, 5.7 Hz, 1H), 4.00 (d, *J* =3.0 Hz, 1H), 3.90 (s, 1H), 3.79 (d, *J* =11.0 Hz, 1H), 3.68 (dd, *J* =11.2, 5.0 Hz, 1H), 3.17 (d, *J* =5.34 Hz, 1H), 1.45 (s, 3H, methyl), 1.28 (s, 3H, methyl).

^{13}C NMR (151 MHz, $CDCl_3$): δ 133.8 (alkene), 117.9 (alkene), 111.7, 105.0 (anomeric), 82.1, 81.9, 79.8, 71.9, 69.1, 64.3, 26.6 (methyl), 26.1 (methyl).

6-(Allyloxy)-2,2-dimethyltetrahydrofuro[2,3-d][1,3]dioxol-5-yl)-2-((*tert*-butyldimethylsilyl) oxy)ethanol (S12)

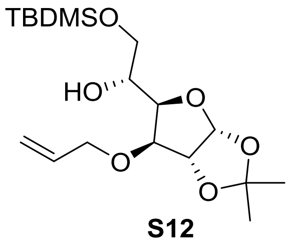

S12

S12 was prepared following literature method [SR9]

To a solution of **S11** (4.5 g, 17.3 mmol) in DCM (45 mL) at 0 °C was added imidazole (2.94 g, 43.27 mmol) and TBDMSCl (2.6 g, 17.3 mmol) and was stirred at 0 °C for 30 min. After completion (TLC), water (30 mL) was added and extracted with DCM (3 × 50 mL). Combined organic fractions were dried over anhydrous Na_2SO_4, concentrated under reduced pressure and purified by column chromatography to afford **S12** compound as a colourless oil (4.9 g, 75%).

R_f 0.55 (20:80, ethyl acetate:hexane).

^{1}H NMR (600 MHz, $CDCl_3$): δ 5.93 – 5.87 (m, 2H), 5.30 (d, *J* =17.2 Hz, 1H, alkene trans-H), 5.18 (d, *J* =10.3 Hz, 1H, alkene cis-H), 4.53 (d, *J* =3.6 Hz, 1H), 4.17 - 4.09 (m, 2H), 4.07 (dd, *J* =8.5, 2.7 Hz, 1H), 4.00 (d, *J* =2.7 Hz, 1H), 3.94– 3.90 (m, 1H), 3.80 (dd, *J* =10.0, 3.6 Hz, 1H), 3.72 (dd, *J* = 10.0, 5.0 Hz, 1H), 2.69 (d, *J* =6.54 Hz, 1H), 1.45 (s, 3H, methyl), 1.29 (s, 3H, methyl), 0.88 (s, 9H, silyl), 0.07 (s, 6H, silyl).

6-(Allyloxy)-2,2-dimethyltetrahydrofuro[2,3-*d*][1,3]dioxol-5-yl)-8,8,9,9-tetramethyl-2,4,7-trioxa-8-siladecane (S13)

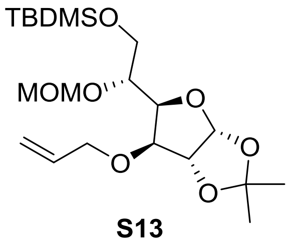

S13

S13 was prepared following literature method [SR10]

To a solution of **S12** (4.0g, 10.69 mmol) in DCM (40 mL) was added DIPEA (3.67 mL, 21.38 mmol) and cooled to 0 °C for 15 minutes. To the solution was added methoxymethane chloride (MOMCl)(1.055 mL, 13.89 mmol) and DMAP (130.6mg, 1.069 mmol) and stirred at room temperature for 3 hours. After completion (TLC), water (50 mL) was added and extracted with DCM (3 × 50 mL). Combined organic layers were dried over anhydrous Na_2SO_4, concentrated under reduced pressure and purified by silica gel column chromatography to afford **S13** as a colourless oil (4.02 g, 90%).

R*f* 0.60 (10:90, ethyl acetate:hexane).

^{1}H NMR (600 MHz, $CDCl_3$): δ 5.90 – 5.84 (m, 2H), 5.26 (d, *J* =17.1 Hz, 1H, alkene trans-H), 5.15 (d, *J* =10.3 Hz, 1H, alkene cis-H), 4.75 – 4.72 (m, 2H), 4.53 (d, *J* =3.7 Hz, 1H), 4.24 (dd, *J* =9.1, 2.8 Hz, 1H), 4.12 (dd, *J* =12.5, 5.4 Hz, 1H), 3.98 – 3.94 (m, 2H), 3.92 (d, *J* =2.8 Hz, 1H), 3.88 – 3.84 (m, 1H), 3.74 (dd, *J* =11.0, 4.4 Hz, 1H), 3.37 (s, 3H, MOM-methyl), 1.44 (s, 3H, methyl), 1.28 (s, 3H, methyl), 0.87 (s, 9H, silyl), 0.04 (s, 6H, silyl).

^{13}C NMR (151 MHz, $CDCl_3$): δ 134.0 (alkene), 117.3 (alkene), 111.5, 105.0 (anomeric), 97.2 (MOM-methylene), 81.6, 81.5, 78.2, 75.8, 70.8, 63.8, 55.6 (MOM-methyl), 26.6 (methyl), 26.3 (methyl), 25.9 (silyl), 18.3 (silyl) , -5.3 (silyl), -5.5 (silyl).

6-(Allyloxy)-2,2-dimethyltetrahydrofuro[2,3-*d*][1,3]dioxol-5-yl)-2-(methoxymethoxy)ethanol (S14)

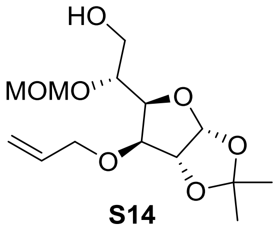

S14

S14 was prepared following literature method [SR4]

1 M Tetrabutylammonium fluoride (11.46 mL, 11.47 mmol, 1N in THF) was added slowly to a solution of **S13** (4.0 g, 9.56 mmol) in THF (20 mL) at 0 °C and stirred at room temperature for 45 min. After completion (TLC), solvent was removed and the residue was taken up in ethyl acetate (50 mL), washed with water (30 mL), brine (30 mL), and dried over Na_2SO_4. The solvent was removed under reduced pressure and crude was purified by silica gel column chromatography to afford the desired product **S14** as colorless oil (2.58 g, 89%).

R_f 0.50 (45:55, ethyl acetate:hexane).

^{1}H NMR (600 MHz, $CDCl_3$): δ 5.86 – 5.79 (m, 2H), 5.25 (dd, *J* =17.7, 1.6 Hz, 1H, alkene trans-H), 5.15 (d, *J* =10.4 Hz, 1H, alkene cis-H), 4.69 – 4.64 (m, 2H), 4.52 – 4.51 (m, 1H), 4.11 (dd, *J* =8.8, 1.7 Hz, 2H), 3.93 – 3.82 (m, 4H), 3.64 – 3.61 (m, 1H), 3.37 (t, *J* =2.4 Hz, 3H, MOM-methyl), 1.43 (s, 3H, methyl), 1.26 (s, 3H, methyl).

^{13}C NMR (151 MHz, CDCl$_3$): δ 133.7 (alkene), 117.5 (alkene), 111.6, 105.1 (anomeric), 97.5 (MOM-methylene), 81.5, 81.4, 79.2, 78.5, 70.8, 63.7, 55.7 (MOM-methyl), 26.6 (methyl), 26.1 (methyl).

(3a*R*,5*R*,6*S*,6a*R*)-5-[(1*R*)-1-(Methoxymethoxy)-2-(prop-2-yn-1-yloxy)ethyl]-2,2-dimethyl-6-(prop-2-en-1-yloxy)-tetrahydro-2*H*-furo[2,3-*d*][1,3]dioxole (1e)

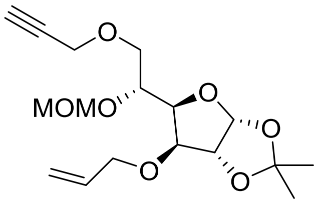

1e

To a solution of **S14** (1.5 g, 4.93 mmol) in DMF (7.5 mL) was added sodium hydride (0.236 g 9.86 mmol) at 0 °C and stirred for 10 min. Then, propargyl bromide (0.700 g, 5.92 mmol) was added and stirred at room temperature for 16 hours. The reaction mixture was diluted with water (50 mL) and extracted in ethyl acetate (3 × 50 mL). Pooled organic layers were washed with brine (50 mL), dried over Na_2SO_4 and solvent was removed under reduced pressure and crude was purified by silica gel column chromatography to furnish **1e** as yellowish syrup (1.09 g, 65%).

R_f 0.71 (30:70, ethyl acetate:hexane)

^{1}H NMR (600 MHz, CDCl$_3$): δ 5.91 – 5.84 (m, 2H), 5.28 (dd,*J* = 17.1, 1.5 Hz, 1H), 5.17 (dd,*J* = 10.4, 0.9 Hz, 1H), 4.73 (q, *J* = 7.4 Hz, 2H), 4.55 (d,*J* = 3.7 Hz, 1H), 4.28 (dd,*J* = 9.1, 3.0 Hz, 1H), 4.26 – 4.12 (m, 3H), 4.02 – 3.93 (m, 4H), 3.64 (dd, *J* = 10.4, 4.5 Hz, 1H), 3.40 (s, 3H. MOM-methyl), 2.39 (t, 1H, alkyne-H), 1.47 (s, 3H, methyl), 1.30 (s, 3H, methyl).

^{13}C NMR (151 MHz, $CDCl_3$): δ 133.8 (alkene), 117.4 (alkene), 11.7, 105.0 (anomeric), 97.1 (MOM-methylene), 81.6, 81.5, 79.8 (alkyne), 78.6, 74.3 (alkyne), 73.9, 70.7, 70.4, 58.6, 55.8 (MOM-methyl), 26.7 (methyl), 26.3 (methyl).

HRMS (TOF, MS, ES+): m/z calcd. for $C_{17}H_{26}NaO_7$: 365.1576; Found:365.1564 [M+Na].

1-(6-Allyloxy-2,2-dimethyltetrahydrofuro[2,3-*d*][1,3]dioxol-5-yl)-2-azidoethanol (2d):

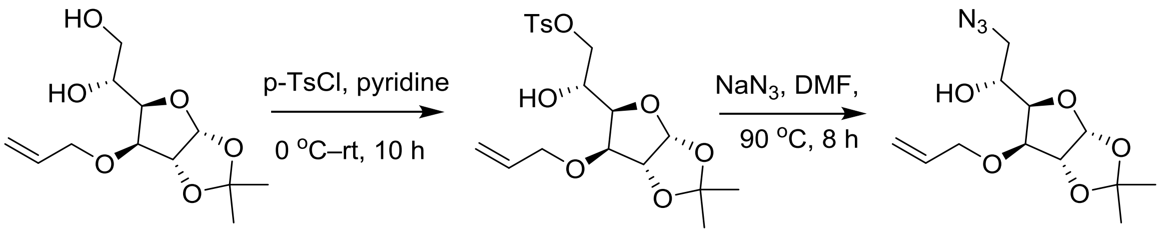

S11 S15 2d

1-(6-Allyloxy-2,2-dimethyltetrahydrofuro[2,3-*d*][1,3]dioxol-5-yl)-2-hydroxyethyl 4-methyl benzenesulfonate (S15):

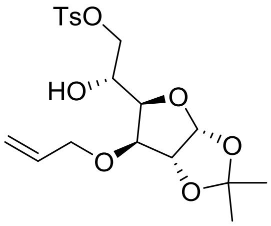

S15

To a solution of **S11** (2.97 g, 11.45 mmol) in pyridine (28 mL) at 0 °C was added *p*-toluenesulfonyl chloride (2.6 g, 13.74 mmol) and stirred for 1 hour and at room temperature for 16 hours. After completion (TLC), the reaction was quenched by adding 1 N HCl solution and extracted the product in ethyl acetate (3 × 100 mL). The pooled organic layer was dried over Na_2SO_4, solvent was removed under reduced pressure to give the crude which was further purified by silica gel column chromatography to furnish **S15** (3.75 g, 79%) as syrup.

R_f 0.58 (25:75, ethyl acetate:hexane).

^{1}H NMR (600 MHz, $CDCl_3$): δ 7.78 (d, *J* = 8.34 Hz, 2H, tosyl), 7.32 (d, *J* = 7.9 Hz, 2H, tosyl), 5.88 – 5.82 (m, 2H, allyl-H,), 5.27 (qd, *J* = 17.2, 1.4 Hz, 1H), 5.19 (dd, *J* = 10.4, 1.3 Hz, 1H), 4.52 (d, *J* = 3.7 Hz, 1H), 4.26 (dd, *J* = 10.2, 2.7 Hz, 1H), 4.18 – 4.01 (m, 6H), 2.42 (s, 3H, tosyl), 1.44 (s, 3H, methyl), 1.28 (s, 3H, methyl).

^{13}C NMR (151 MHz, $CDCl_3$): δ 149.6 (tosyl), 144.9 (tosyl), 133.6 (alkene), 129.9 (tosyl), 128.0 (tosyl), 118.0 (alkene), 111.9, 105.1 (anomeric), 82.1, 81.7, 79.2, 72.4, 71.2, 67.2, 26.7 (methyl), 26.2 (methyl), 21.6 (tosyl-methyl).

1-(6-Allyloxy-2,2-dimethyltetrahydrofuro[2,3-*d*][1,3]dioxol-5-yl)-2-azidoethanol (2d):

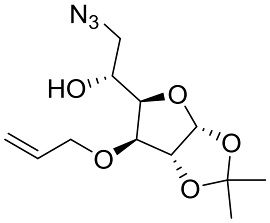

2d

To a solution of **S15** (3 g, 7.24 mmol) in DMF (15 mL) was added NaN_3 (0.94 g, 14.48 mmol) and mixture was heated at 90 °C for 16 hours. After completion (TLC), reaction mixture was diluted with water (50 mL) and extracted in ethyl acetate (3 × 50 mL). Pooled organic layers were washed with brine (50 mL), dried over Na_2SO_4, solvent was removed under reduced pressure and residue was purified by silica gel column chromatography to furnish **2d** (1.59 g, 82%).

R_f 0.65 (30:70, ethyl acetate:hexane)

^{1}H NMR (600 MHz, CDCl₃): δ 5.91 – 5.84 (m, 2H, allyl-H), 5.29 (d, *J* = 17.1 Hz, 1H), 5.21 (d, *J* = 10.3 Hz, 1H), 4.55 (d, *J* = 3.7 Hz, 1H), 4.16 (dd, *J* = 12.6, 5.3 Hz, 1H), 4.07 (s, 2H), 4.04 – 4.00 (m, 2H), 3.55 (d, *J* = 12.8 Hz, 1H), 3.43 (td, *J*=12.7, 2.7 Hz, 1H), 2.71 (s, 1H), 1.46 (s, 3H, methyl), 1.29 (s, 3H, methyl).

^{13}C NMR (151 MHz, $CDCl_3$): δ 128.8 (alkene), 113.5 (alkene), 107.1, 100.4 (anomeric), 77.3, 77.0, 75.2, 66.3, 63.7, 49.9, 22.0 (methyl), 21.5 (methyl).

HRMS (TOF, MS, ES+): m/z calcd. for $C_{12}H_{19}N_3NaO_5$: 308.1222; Found: 308.1207 [M+Na].

(3a*R*,5*S*,6*S*,6a*R*)-6-(Allyloxy)-5-((*R*)-2-azido-1-(methoxymethoxy)ethyl)tetrahydro-2,2-dimethylfuro[2,3-*d*][1,3]dioxole (2c):

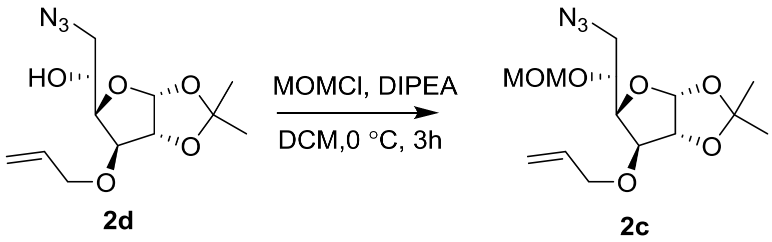

To a solution of **2d** (2.2 g, 7.71 mmol) in DCM (22 mL) was added DIPEA (2.64 mL, 15.4 mmol) and cooled to 0 ºC for 15 minutes. To this, MOMCl (0.76 mL, 10.0 mmol) and DMAP (94.2 mg, 0.77 mmol) was added and stirred at room temperature for 3 hours. After completion (TLC), water (50 mL) was added and was extracted with DCM (3 × 20 mL). Combined organic layers were dried over anhydrous Na_2SO_4, concentrated under reduced pressure and purified by column chromatography to afford **2c** as a colourless oil (1.27 g, 50.2%).

R_f 0.73 (20:80, ethyl acetate:hexane).

^{1}H NMR (600MHz, CDCl$_3$): δ 5.89-5.83 (m, 2H), 5.27 (d, *J* = 17.4 Hz, 1H), 5.18 (d, *J* = 10.4 Hz, 1H), 4.74 (d , *J* = 6.4 Hz, 1H), 4.68 (d, *J* = 6.4 Hz, 1H), 4.55 (d, *J* =3.9 Hz,1H), 4.24 (dd, *J* = 8.8, 3.0 Hz, 1H), 4.13 (dd, *J* = 12.8, 5.8 Hz), 4.00-3.93 (m, 3H), 3.67 (d, *J* = 12.8 Hz, 1H), 3.48 (dd, *J* = 12.8, 4.2 Hz, 1H), 3.41 (s, 3H, MOM-methyl), 1.48 (s, 3H, methyl), 1.30 (s, 3H, methyl).

^{13}C NMR (151MHz, CDCl$_3$): 133.7 (alkene), 117.6 (alkene), 111.9, 105.0 (anomeric), 97.5 (MOM-methylene), 81.6, 81.3, 78.9, 74.6, 70.7, 55.9 (MOM-methyl), 53.1, 26.8 (methyl), 26.3 (methyl).

HRMS (TOF, MS, ES+): m/z calcd. for $C_{14}H_{23}N_3O_6Na$: 352.1479; found: 352.1488 (M+Na$^+$).

[2-Hydroxy-2-(2,2-dimethyl-6-(prop-2-ynyloxy)tetrahydro-furo[2,3-*d*][1,3]dioxol-5-yl)-ethoxy]-*tert*-butyl-dimethyl-silane (S18):

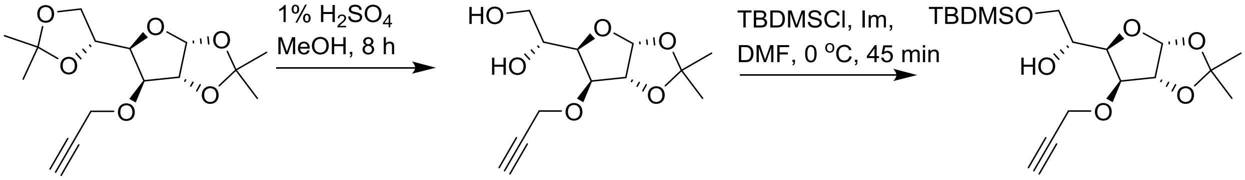

S16 **S17** **S18**

5-((*R*)-2,2-Dimethyl-1,3-dioxolan-4-yl)-2,2-dimethyl-6-(prop-2-yn-1-yloxy)tetrahydrofuro[2,3-*d*][1,3]dioxole (S16)

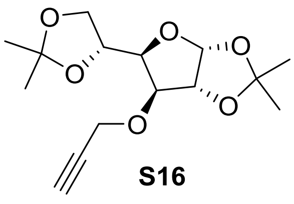

S16

S16 was prepared following literature method [SR11]

To a solution of **S9** (6 g, 23.1 mmol) in DMF (30 mL) was added sodium hydride (1.107 g, 46 mmol) at 0 °C and stirred for 10 min. Then, propargyl bromide (2.39 mL, 28 mmol) was added and stirred at room temperature for 16 hours. After completion (TLC), reaction mixture was diluted with water (50 mL) and extracted in ethyl acetate (3 × 50 mL). Pooled organic layers were washed with brine (50 mL), dried over Na_2SO_4 and solvent was removed under reduced pressure and crude was purified by silica gel column chromatography to furnish **S16** as colourless oil (6.8 g, 99%).

R*f* 0.38 (10:90, ethyl acetate:hexane).

^{1}H NMR (600 MHz, $CDCl_3$): δ 5.82 (d, *J* = 3.6 Hz, 1H), 4.57 (d, *J* = 3.6 Hz, 1H), 4.23 – 4.19 (m, 3H), 4.08 (dd, *J* = 7.6, 2.9 Hz, 1H), 4.04 – 4.01 (m, 2H), 3.93 (dd, *J* = 8.6, 5.4 Hz, 1H), 2.45 (t, *J* =2.3 Hz, 1H, alkyne), 1.44 (s, 3H, methyl), 1.37 (s, 3H, methyl), 1.29 (s, 3H, methyl), 1.26 (s, 3H, methyl).

^{13}C NMR (151 MHz, $CDCl_3$): δ 111.8, 108.9, 105.1 (anomeric), 82.8, 81.5, 81.0, 79.2 (alkyne), 74.9 (alkyne), 72.4, 67.1, 58.0, 26.7 (methyl), 26.7 (methyl), 26.2 (methyl), 25.3 (methyl).

(1*R*)-1-[(3a*R*,5*R*,6*S*,6a*R*)-2,2-Dimethyl-6-(prop-2-yn-1-yloxy)tetrahydro-2*H*-furo[2,3-*d*][1,3]dioxol-5-yl]ethane-1,2-diol (S17)

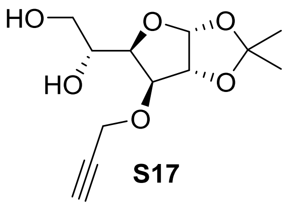

S17

Further, to a solution of crude **S16** (6.8 g, 22.79 mmol) in methanol (34 mL) at room temperature was added 1% aq. H_2SO_4 solution (23 mL) and stirred for 8 h. After completion (TLC), the reaction mixture was quenched with satd. aq. $NaHCO_3$ and extracted with ethyl acetate (50 mL ×

3). Pooled organic layers were dried over Na_2SO_4, solvent was removed under reduced pressure and residue was purified by silica gel column chromatography (ethyl acetate: hexane) to afford the diol **S17** (4.8 g, 81%) as pale yellow syrup.

R*f* 0.47 (70:30, ethyl acetate:hexane).

^{1}H NMR (600 MHz, $CDCl_3$): δ 5.87 (d, *J* = 3.7 Hz, 1H), 4.59 (d, *J* = 3.7 Hz, 1H), 4.32 – 4.21 (m, 2H), 4.19 (d, *J* = 3.1 Hz, 1H), 4.11 (dd, *J* = 8.4, 3.1 Hz, 1H), 3.95 – 3.92 (m, 1H), 3.81 (dd, *J* = 11.5, 3.1 Hz, 1H), 3.70 – 3.67 (m, 1H), 2.53 (t, *J* =2.3 Hz, 1H, alkyne-H), 1.46 (s, 3H, methyl), 1.28 (s, 3H, methyl).

^{13}C NMR (151 MHz, $CDCl_3$): δ 111.9, 105.1 (anomeric), 82.1, 81.5, 79.7 (alkyne), 79.1, 75.5 (alkyne), 68.9, 64.2, 57.6, 26.6 (methyl), 26.1 (methyl).

(1*R*)-1-[(3a*R*,5*R*,6*S*,6a*R*)-2,2-Dimethyl-6-(prop-2-yn-1-yloxy)tetrahydro-2*H*-furo[2,3-d][1,3]dioxol-5-yl]-2-[(*tert*-butyldimethylsilyl)oxy]ethan-1-ol (S18)

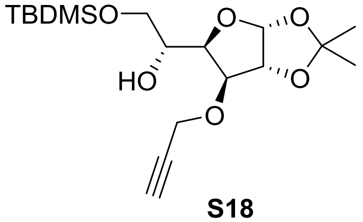

S18

Further, to solution of crude diol **S17** (3.5 g, 13.55 mmol) in dry DMF (17.5 mL) at 0 °C were added TBDMSCl (2.01 g, 13.41 mmol) and imidazole (2.03 g, 29.81 mmol) and stirred at 0 °C for 45 min. After completion (TLC), white precipitate was filtered off, and the filtrate was evaporated to dryness under high vacuum. The residue was dissolved in ethyl acetate (100 mL), washed with water (50 mL) and brine (50 mL), dried over Na_2SO_4, solvent was removed under reduced pressure to furnish **S18** as colorless oil (3.33 g, 66% crude).

R_f 0.45 (40:60, ethyl acetate:hexane);

^{1}H NMR (600 MHz, $CDCl_3$): δ 5.85 (d, *J* = 3.6 Hz, 1H), 4.59 (d, *J* = 3.6 Hz, 1H), 4.32 – 4.26 (m, 2H), 4.16 (d, *J* = 2.4 Hz, 1H), 4.08 (dd, *J* = 8.8, 2.7 Hz, 1H), 3.87 – 3.84 (m, 1H), 3.81 (dd, *J* = 10.1, 3.4 Hz, 1H), 3.71 – 3.69 (m, 1H), 2.47 (t, *J* =2.3 Hz, 1H, alkyne-H), 1.44 (s, 3H, methyl), 1.28 (s, 3H, methyl), 0.87 (s, 9H, silyl), 0.05(s, 6H, silyl).

^{13}C NMR (151 MHz, $CDCl_3$): δ 111.7, 105.1 (anomeric), 82.5, 81.5, 79.3 (alkyne), 75.0 (alkyne), 68.4, 64.4, 57.9, 26.6 (methyl), 26.2 (methyl), 25.8 (silyl), 18.2 (silyl), -5.4 (silyl).

2-Allyloxy-2-(2,2-dimethyl-6-(prop-2-ynyloxy)tetrahydrofuro[2,3-*d*][1,3]dioxol-5-yl)-ethanol (1f):

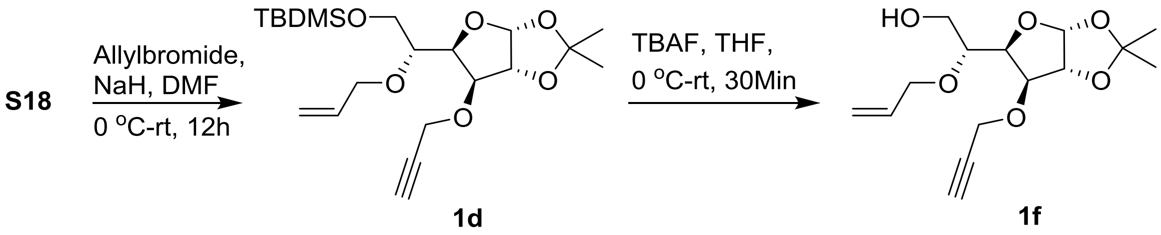

[(2*R*)-2-[(3a*R*,5*R*,6*S*,6a*R*)-2,2-Dimethyl-6-(prop-2-yn-1-yloxy)tetrahydro-2*H*-furo[2,3-*d*][1,3]dioxol-5-yl]-2-(prop-2-en-1-yloxy)ethoxy](*tert*-butyl)dimethylsilane (1d)

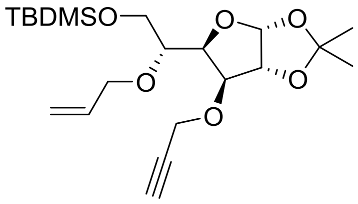

1d

To a solution of **S18** (3.9 g, 1.05 mmol) in DMF (20 mL) under argon was added sodium hydride (0.503 g, 20.96 mmol) at 0 °C and stirred for 10 min. Allyl bromide (1.08 mL, 12.58 mmol) was added and reaction mixture was warmed to room temperature and stirred for 12 hours. After completion (TLC), the reaction mixture was diluted with water (40 mL) and extracted in ethyl

acetate (3 × 60 mL). Pooled organic phase was washed with brine, dried over Na_2SO_4, solvent was removed under reduced pressure and purified by silica gel column chromatography to furnish **1d** in 81% yield.

R*f* 0.61 (40:60, ethyl acetate:hexane);

^{1}H NMR (600MHz, CDCl$_3$): δ 5.94-5.88 (m, 1H, allyl-H), 5.84 (m,1H), 5.25-5.21 (m, 1H), 5.10-5.09 (dd, *J* = 10.4, 1.6 Hz, 1H), 4.59-4.58 (m, 1H), 4.30-4.27 (m, 1H), 4.25-4.18 (m, 2H), 4.15-4.13 (dd, *J* = 8.9, 1.3 Hz, 1H), 4.10 (m, 1H), 4.08-4.05 (m, 1H), 3.95-3.93 (m, 1H), 3.70-3.64 (m, 2H), 2.44 (t, *J* = 2.4 Hz, 1H, alkyne-H), 1.43 (s, 3H, methyl), 1.27 (s, 3H, methyl), 0.86 (s, 9H, silyl), 0.03 (s, 6H, silyl).

^{13}C NMR (151MHz, CDCl$_3$): δ 135.4 (alkene), 116.4, 111.6, 105.0 (anomeric), 81.8, 81.4, 79.1, 78.5, 76.5, 75.1 (alkyne) 71.9, 64.0, 57.5, 26.7 (methyl), 26.3 (methyl), 25.9, 18.3 (silyl), -5.4 (silyl-methyl), -5.5(silyl-methyl).

HRMS (TOF, MS, ES+): m/z calcd. for $C_{21}H_{37}O_6Si$: 413.2354; found: 413.2373 (M+H$^+$).

(2*R*)-2-[(3a*R*,5*R*,6*S*,6a*R*)-2,2-Dimethyl-6-(prop-2-yn-1-yloxy)tetrahydro-2*H*-furo[2,3-*d*][1,3] dioxol-5-yl]-2-(prop-2-en-1-yloxy)ethan-1-ol (1f)

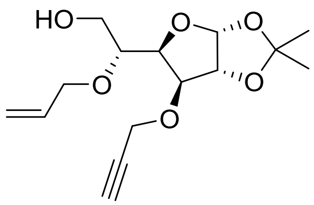

1f

Further, 1 M tetrabutylammonium fluoride (TBAF) (9.36 mL, 9.35 mmol) was added slowly to a solution of **1d** (3.5 g, 8.5 mmol) in THF (17.5 mL) at 0 °C. The dark brown solution was stirred at 0 °C for 30 min, solvent was removed under reduced pressure. Residue was dissolved in ethyl

acetate (100 mL), washed with water (50 mL) and brine (50 mL) and dried over Na_2SO_4. Solvent was removed under reduced pressure and crude was purified by silica gel column chromatography (ethyl acetate: hexane) to afford the desired product **1f** as colorless oil (2.02 g, 81%).

R_f 0.39 (40:60, ethyl acetate:hexane);

^{1}H NMR (600 MHz, $CDCl_3$): δ 5.96 – 5.89 (m, *J* = 16.1, 10.9, 5.7 Hz, 1H), 5.86 (d, *J* = 3.7 Hz, 1H), 5.28 (dd, *J* = 17.2, 1.5 Hz, 1H), 5.16 (dd, *J* = 10.3, 1.5 Hz, 1H), 4.62 (d, *J* = 3.7 Hz, 1H), 4.29 – 4.22 (m, 3H), 4.18 – 4.14 (m, 2H), 4.09 (dd, *J* = 12.4, 5.3 Hz, 1H), 3.90 – 3.86 (dd, *J* = 12.4, 5.3 Hz, 1H), 3.75 – 3.70 (m, 2H), 2.47 (t, *J* = 2.4 Hz, 1H), 2.17 (bs, 1H, -OH), 1.48 (s, 3H, methyl), 1.30 (s, 3H, methyl).

^{13}C NMR (151 MHz, $CDCl_3$): δ 134.7 (alkene), 117.1 (alkene), 111.9, 105.0 (anomeric), 81.5, 81.1, 79.5, 78.9 (alkyne), 75.4, 75.3 (alkyne), 71.2, 61.8, 57.3, 26.7 (methyl), 26.3 (methyl).

HRMS (TOF, MS, ES+): m/z calcd. for $C_{15}H_{23}NaO_6$: 321.1309, Found:321.1301 [M+Na].

(3a*R*,5*S*,6*R*,6a*R*)-6-Azido-2,2-dimethyl-5-[(prop-2-en-1-yloxy)methyl]tetrahydro-2*H*-furo[2,3-*d*][1,3]dioxole (2b)

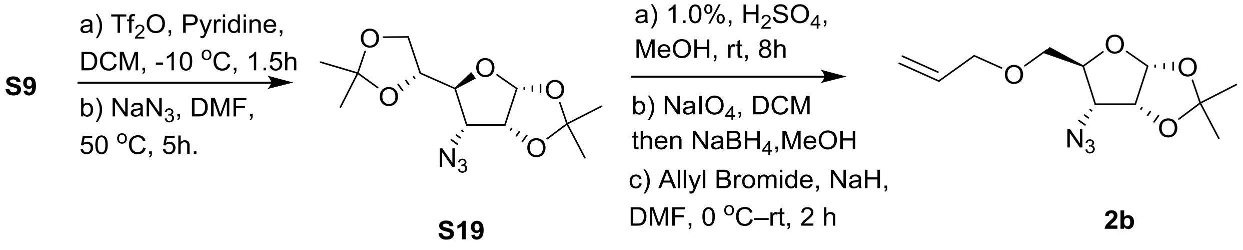

The compound **S19** was synthesized following literature procedure [SR12]. Thus, to a three-necked flask charged with **S9** (3 g, 11.54 mmol) in dichloromethane (30 mL) under argon cooled at −10 °C was slowly added triflic anhydride (6.5 g, 23.08 mmol) and pyridine (3.65 g, 0.384

mmol). After completion (TLC) reaction mixture was poured onto ice water (1 L) and the aqueous phase was extracted with dichloromethane (4 × 100 mL). Pooled organic layers were dried over Na_2SO_4, and repeatedly co-evaporated with toluene to remove pyridine. The brown residue was extracted with hexane (3 × 50 mL) and evaporation of hexane yielded the desired product as white crystals (4.25 g, 98%). A solution of triflyl sugar (3.2 g, 8.5 mmol) in DMF (5 mL) was slowly added to a solution of NaN_3 (1.105 g, 17 mmol), Bu_4NCl (0.1 g) in DMF (10 mL) at 50 °C. After completion (TLC), DMF was removed under reduced pressure, and the residue was dissolved in ethyl acetate (200 mL) and washed with H_2O (2 × 50 mL). The aqueous phase was re-extracted with ethyl acetate (2 × 50 mL), pooled organic layers were dried over Na_2SO_4 and evaporated to yield a syrup of crude **S19** along with elimination product. (^{1}H NMR confirms the ratio of **S19** to elimination product of 7:3). The crude product was purified by silica gel column (ethyl acetate:hexane) to yield **S19** as colorless liquid (1.71 g, 70%). **R*f*** 0.55 (1:3, ethyl acetate:hexane).

(3a*R*,5*S*,6*R*,6a*R*)-6-Azido-2,2-dimethyl-5-[(prop-2-en-1-yloxy)methyl]tetrahydro-2*H*-furo[2,3-*d*][1,3]dioxole (2b)

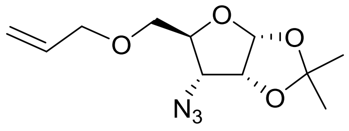

2b

To a solution of crude **S19** (1.70 g, 5.96 mmol) in 17 mL methanol at room temperature was added 11 mL, 1.0% aq. H_2SO_4 solution and stirred for 8 hours. After completion (TLC), the reaction mixture was quenched with satd. aq $NaHCO_3$ and extracted with ethyl acetate (3 × 50 mL). Pooled organic layers were dried over Na_2SO_4, solvent was removed under reduced pressure and crude was taken for next step. To a stirred solution of crude diol obtained from **S19**

(1.03 g, 3.61 mmol) in DCM (14 mL) was added $NaIO_4$ (926 mg, 4.33 mmol) adsorbed over silica gel at 0 °C and kept stirring for 4 hours. The reaction mixture was diluted with MeOH (15 mL), the inorganic salts were filtered off, and the filtrate was evaporated to obtain a light yellow syrup. The crude product was dissolved in MeOH (10 mL) and THF (4 mL) and was added $NaBH_4$ (185 mg, 4.9 mmol) and the reaction mixture stirred for 3 hours at 0 °C and filtered. The solvent was evaporated to give crude alcohol as white solid (0.691 g, 78%) which was taken for the next step without further purification. To a solution of the crude alcohol (280 mg, 1.1 mmol) in DMF (1 mL) was added sodium hydride (0.052 g 2.2 mmol) at 0 °C, and allyl bromide (0.114 mL,1.32 mmol) and stirred at room temperature for 12 hours. After completion (TLC), the reaction mixture was diluted with water (5 mL) and extracted in ethyl acetate (3 × 20 mL). Pooled organic layers were washed with brine, dried over Na_2SO_4 and solvent was removed under reduced pressure and residue was purified by silica gel column chromatography to afford **2b** (0.250 g, 86%).

R_f 0.50 (20:80, ethyl acetate:hexane);**^{1}H NMR (600 MHz, $CDCl_3$):** δ 5.90 – 5.83 (m, 1H, allyl-H), 5.79 (d, *J* = 3.7 Hz, 1H), 5.25 (dd, *J* = 17.3, 1.51 Hz, 1H), 5.17 (d, *J* = 10.5 Hz, 1H), 4.68 (t, *J* = 4.3 Hz, 1H), 4.19 – 4.16 (m, 1H), 4.05 (dd, *J* = 12.9, 5.7 Hz, 1H), 4.00 (dd, *J* = 12.9, 5.7 Hz, 1H), 3.77 (dd, *J* = 11.6, 2.5 Hz, 1H), 3.58 (dd, *J* = 11.6, 3.6 Hz, 1H), 3.55 (dd, *J* = 9.6, 4.6 Hz, 1H), 1.56 (s, 3H, methyl), 1.35 (s, 3H, methyl).

^{13}C NMR (151 MHz, $CDCl_3$): δ 134.2 (alkene), 117.6 (alkene), 113.0, 104.1 (anomeric), 79.9, 77.2, 72.6, 67.6, 60.4, 26.4 (methyl), 26.3 (methyl).

HRMS (TOF, MS, ES+): m/z calcd. for $C_{11}H_{17}N_3NaO_4$: 278.1111; Found: 278.1106 [M+Na].

(3a*S*,5*R*,6*R*,6a*S*)-6-(Allyloxy)-2,2-dimethyl-5-((prop-2-yn-1-yloxy)methyl)-tetrahydrofuro[2,3-*d*][1,3] dioxole (1c):

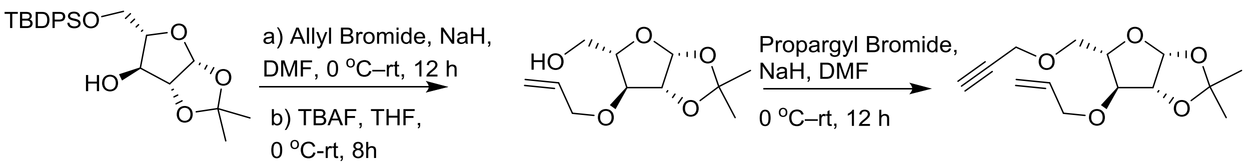

S20 **S21** **1c**

The compound **S20** and **S21** were prepared following literature method [SR13].

(3a*R*,5*S*,6*S*,6a*R*)-5-{[(*tert*-Butyldiphenylsilyl)oxy]methyl}-2,2-dimethyltetrahydro-2*H*-furo[2,3-*d*][1,3]dioxol-6-ol (S20)

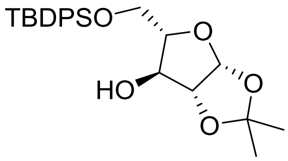

S20

To a solution of L-arabinose (10 g, 66.67 mmol) in dry DMF (50 mL) was added imidazole (9.10 g, 133.4 mmol), TBDPSCl (11.83 g, 66.67 mmol) and resulting mixture was stirred at 60 °Cfor 2 hours. After completion (TLC), mixture was poured into 50 mL 1 N HCl and extracted in DCM (3 × 150 mL). Pooled organic layers were washed sequentially with water (50 mL), sat. $NaHCO_3$ (50 mL) and brine (50 mL). The organic layer was dried over Na_2SO_4, solvent was removed under reduced pressure and crude was purified by silica gel column chromatography to furnish silyl ether as colourless oil (13.0 g, 50%).

Further, to a solution of silyl ether (13.0 g, 33.42 mmol) in anhydrous acetone (260 mL) at room temperature was added anhydrous $CuSO_4$ (15.9 g, 100.2 mmol). The solution was cooled to 0 °C and was added conc. H_2SO_4 (9.53 mL, 0.66 M in acetone) stirred at room temperature for 17 hours. After completion (TLC), reaction mixture was neutralized with a saturated K_2CO_3 solution. The solution was filtered and the filtrate was evaporated under reduced pressure. The

residue obtained was extracted in DCM (3 × 150 mL). Combined organic layer was dried over anhydrous Na_2SO_4, concentrated under reduced pressure. The crude was purified by silica gel column chromatography to afford **S20** (8.01 g, 56%) as colourless oil.

R*f* 0.55 (20:80, ethyl acetate:hexane).

[(3a*R*,5*S*,6*S*,6a*R*)-2,2-Dimethyl-6-(prop-2-en-1-yloxy)tetrahydro-2*H*-furo[2,3-*d*][1,3]dioxol-5-yl]methanol (S21)

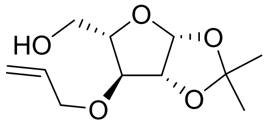

S21

S21 was prepared following literature method [SR14, SR15]

To a solution of **S20** (7.8 g, 18.2 mmol) in DMF (39 mL) was added sodium hydride (0.87 g, 36.4 mmol) at 0 °C and stirred for 10 min. Then, allyl bromide (1.86 mL, 21.8 mmol) was added and stirred at room temperature for 2 hours. The reaction mixture was diluted with water (50 mL) and extracted in ethyl acetate (3 × 100 mL). Pooled organic layers were washed with brine (50 mL), dried over Na_2SO_4 and solvent was removed under reduced pressure and the crude product was purified by silica gel column chromatography to furnish a colourless oil (6.15 g, 72%).

Further, 1 M TBAF (3.77 g, 14.4 mmol) was added slowly to a solution of the above oil (6.15 g, 13.1 mmol) in THF (30 mL) at 0 °C. The colourless solution turned dark during the addition. The dark solution was stirred at 0 °C for 2 hours. After completion (TLC), the reaction mixture was diluted with water (50 mL) and extracted in ethyl acetate (3 × 100 mL). Pooled organic layers were washed with brine (50 mL), dried over Na_2SO_4 and solvent was removed under

reduced pressure and crude was purified by silica gel column chromatography to afford **S21** as colourless oil (2.5 g, 83%).

R*f* 0.57 (40:60, ethyl acetate:hexane).

^{1}H NMR (600 MHz, $CDCl_3$): δ 5.89 – 5.82 (m, 2H), 5.27 (dd, *J* =17.1, 1.5 Hz, 1H), 5.19 (dd, *J* =10.3, 1.2 Hz, 1H), 4.58 (d, *J* =3.9 Hz, 1H), 4.11 – 4.06 (m, 2H), 4.00 (dd, *J* =12.6, 5.7 Hz, 1H), 3.88 (d, *J* =2.8 Hz, 1H), 3.74 – 3.69 (m, 1H), 1.50 (s, 3H, methyl), 1.30 (s, 3H, methyl).

^{13}C NMR (151 MHz, $CDCl_3$): δ 133.7 (alkene), 117.7 (alkene), 112.8, 105.5 (anomeric), 85.6, 85.1, 82.6, 70.7, 62.6, 27.0 (methyl), 26.2 (methyl).

(3a*R*,5*S*,6*S*,6a*R*)-2,2-Dimethyl-6-(prop-2-en-1-yloxy)-5-[(prop-2-yn-1-yloxy)methyl]-tetrahydro-2H-furo[2,3-d][1,3]dioxole 1c

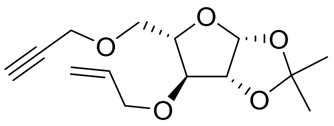

1c

To a solution of **S21** (0.5 g, 2.17 mmol) in DMF (2.5 mL) was added sodium hydride (104.16 mg, 4.34 mmol) and propargyl bromide (224 µL, 2.6 mmol) at 0 ^{o}C and solution was stirred at room temperature for 12 hours. After completion (TLC), the reaction mixture was diluted with water (30 mL) and extracted in ethyl acetate (3 × 60 mL). Pooled organic layers were washed with brine (1 × 60 mL), dried over Na_2SO_4, solvent was removed under reduced pressure to give the crude which was purified by silica gel column chromatography to afford **1c** as yellowish syrup (0.520 g, 89%).

R*f* 0.59 (20:80, ethyl acetate:hexane).

^{1}H NMR (600 MHz, $CDCl_3$): δ 5.89 – 5.81 (m, 1H, allyl-H), 5.83 (d, *J* = 3.88 Hz, 1H), 5.27 (dd, *J* = 17.2, 1.5 Hz, 1H), 5.17 (dd, *J* = 10.4, 1.5 Hz, 1H), 4.55 (d, *J* = 3.9 Hz, 1H), 4.19 – 4.16 (m, 2H), 4.13 (td, *J* = 6.2, 3.3 Hz, 1H), 4.06 – 3.98 (m, 2H), 3.91 (d, *J* = 2.3 Hz, 1H), 3.67 (d, *J* = 6.3 Hz, 2H), 2.41 (t, *J* = 2.4 Hz, 1H, alkyne-H), 1.50 (s, 3H, methyl), 1.29 (s, 3H, methyl).

^{13}C NMR (151 MHz, $CDCl_3$): δ 133. 8 (alkene), 117.6 (alkene), 112.6, 105.7 (anomeric), 84.9, 83.5, 83.0, 79.5 (alkyne), 74.7 (alkyne), 70.6, 69.7, 58.5, 27.0 (methyl), 26.2 (methyl).

HRMS (TOF, MS, ES+): m/z calcd. for $C_{14}H_{20}NaO_5$: 291.1208; Found: 291.1197 [M+Na].

Synthesis of cycloadducts

General procedure A: Copper catalyzed azide–alkyne cycloaddition (CuAAC) reaction

To a suspension of propargyl ether (1.0 eq) and azide (1.0 eq) in water (5 mL/mmol) was added Copper (I) Iodide (5 mol %) at room temperature and the resulting suspension was stirred for the 2 hour at 70 °C. After completion (TLC), the reaction mixture was concentrated *in vacuo*, residue adsorbed over silica gel and purified by silica gel column chromatography eluting with ethyl acetate-hexane to furnish cycloadduct.

4-((((3a*R*,5*R*,6*S*,6a*R*)-6-(Allyloxy)-tetrahydro-2,2-dimethylfuro[3,2-*d*][1,3]dioxol-5-yl)methoxy)methyl)-1-(((3a*R*,5*R*,6*S*,6a*R*)-6-(allyloxy)tetrahydro-2,2-dimethylfuro[2,3-*d*][1,3]dioxol-5-yl)methyl)-1*H*-1,2,3-triazole (3a): Following the general procedure A, the title compound (**3a**) was obtained as yellow syrup (Yield 95%), **R_f** 0.50 (5:95, MeOH: dichloromethane) **^{1}H NMR ($CDCl_3$, 600 MHz):** δ 7.67 (s, 1H), 5.95 (d, *J* = 3.7 Hz, 1H), 5.91 (d, *J* = 3.8 Hz, 1H), 5.90 – 5.80 (m, 2H), 5.32 (dd, *J* = 17.3, 1.2 Hz, 1H), 5.24 (d, *J* = 8.9 Hz, 1H),

5.23 (s, 1H), 5.18 (dd, *J* = 10.3, 1.2 Hz, 1H), 4.73 – 4.69 (m, 2H), 4.65 (d, *J* = 12.3 Hz, 1H), 4.60 (d, *J* = 3.7 Hz, 1H), 4.56-4.48 (m, 3H), 4.49 (dd, *J* = 13.6, 7.7 Hz, 1H), 4.37 (m, 1H), 4.18 (dd, *J* = 12.6, 5.1 Hz, 1H), 4.10 (dd, *J* = 12.6, 5.1 Hz, 1H), 3.98 (dd, *J* = 12.8, 5.6 Hz, 2H), 3.89 (dd, *J* = 13.0, 3.1 Hz, 2H), 3.80 (dd, *J* = 10.0, 5.7 Hz, 1H), 3.76 (dd, *J* = 10.0, 6.5 Hz, 1H), 1.45 (s, 3H), 1.43 (s, 3H), 1.30 (s, 6H); **^{13}C NMR (151 MHz, $CDCl_3$):** δ 133.6, 133.4, 118.2, 117.0, 112.0, 111.6, 105.2, 105.0, 82.4, 82.1, 81.7, 81.6, 79.1, 78.8, 71.0, 68.0, 64.9, 49.1, 26.8, 26.7, 26.3, 26.2; **HRMS (TOF, MS, ES+):** m/z calcd. for $C_{25}H_{38}N_3O_9$: 524.2603; found: 524.2590 ($M+H^+$).

4-(((3a*R*,5*R*,6*S*,6a*R*)-5-((Allyloxy)methyl)-dihydro-2,2-dimethyl-5*H*-furo[2,3-*d*][1,3]dioxol-6-yloxy)methyl)-1-(((3a*R*,5*R*,6*S*,6a*R*)-6-(allyloxy)tetrahydro-2,2-dimethylfuro[3,2-*d*][1,3]-dioxol-5-yl)methyl)-1*H*-1,2,3-triazole (3b): Following the general procedure A, the title compound (**3b**) was obtained as syrup (Yield 94%), **R_f** 0.60 (5:95, MeOH:dichloromethane); **^{1}H NMR (600 MHz, $CDCl_3$):** δ 7.65 (s, 1H), 5.94 (d, *J* = 3.4 Hz, 1H), 5.89 (d, *J* = 3.4 Hz, 1H), 5.92 – 5.83 (m, 2H), 5.31 (d, *J* = 17.2 Hz, 1H), 5.26 (d, *J* = 10.2 Hz, 1H), 5.24 (s, 1H), 5.16 (d, *J* = 10.2 Hz, 1H), 4.75 (d, *J* = 12.4 Hz, 1H), 4.71 (dd, *J* = 12.6, 3.4 Hz, 1H), 4.67 (d, *J* = 12.7 Hz, 1H), 4.60 (d, *J* = 3.2 Hz, 2H), 4.55 – 4.46 (m, 2H), 4.38 – 4.34 (m, 1H), 4.18 (dd, *J* = 12.6, 5.0 Hz, 1H), 4.05 – 4.01 (m, 2H), 3.98 (dd, *J* = 12.9, 5.0 Hz, 2H), 3.92 (d, *J* = 3.0 Hz, 1H), 3.69 – 3.61 (m, 2H), 1.47 (s, 3H), 1.42 (s, 3H), 1.29 (s, 6H); **^{13}C NMR (151 MHz, $CDCl_3$):** δ 144.7, 134.7, 133.5, 123.8, 118.4, 117.3, 112.2, 111.8, 105.3, 105.1, 82.5, 82.2, 82.0, 81.8, 79.0, 78.9, 72.5, 71.1, 67.3, 63.9, 49.4, 26.4, 26.4, 26.3, 26.3; **HRMS (TOF, MS, ES+):** m/z calcd. for $C_{25}H_{38}N_3O_9$: 524.2603; found: 524.2595 ($M+H^+$).

4-(((3a*R*,5*R*,6*S*,6a*R*)-5-((Allyloxy)methyl)dihydro-2,2-dimethyl-5*H*-furo[2,3-*d*][1,3]dioxol-6-yloxy)methyl)-1-((3a*R*,5*S*,6*R*,6a*R*)-5-((allyloxy)methyl)-tetrahydro-2,2-dimethylfuro[3,2-*d*][1,3]dioxol-6-yl)-1*H*-1,2,3-triazole (3c): Following the general procedure A, the title

compound (**3c**) was obtained as yellow syrup (Yield 75%); $\mathbf{R}_f$ 0.52 (50:50, ethyl acetate:hexane); **^{1}H NMR** (600 MHz, $CDCl_3$): δ 7.76 (s, 1H), 5.97 (d, *J* = 3.7 Hz, 1H), 5.86 (d, *J* = 3.7 Hz, 1H), 5.90 – 5.76 (m, 2H), 5.23 (d, *J* = 17.2 Hz, 1H), 5.17 (d, *J* = 17.2 Hz, 1H), 5.14 (d, *J* = 3.4 Hz, 1H), 5.15 – 5.12 (m, 2H), 4.82 (t, *J* = 4.0 Hz, 1H), 4.75 (d, *J* = 12.2 Hz, 1H), 4.68 (d, *J* = 12.2 Hz, 1H), 4.57 (d, *J* = 3.7 Hz, 1H), 4.55 – 4.50 (m, 1H), 4.38 – 4.35 (m, 1H), 4.01 (dd, *J* = 11.1, 3.6 Hz, 2H), 3.93 (m, 3H), 3.70 – 3.68 (m, 2H), 3.62 (dd, *J* = 10.0, 6.58 Hz, 1H), 3.48 (dd, *J* = 11.8, 3.9 Hz, 1H), 1.58 (s, 3H), 1.44 (s, 3H), 1.32 (s, 3H), 1.27 (s, 3H); **^{13}C NMR (151 MHz, $CDCl_3$):** δ 144.5, 134.6, 134.0, 122.9, 117.6, 117.1, 113.5, 111.7, 105.0, 104.5, 82.4, 82.2, 78.9, 78.7, 77.9, 72.6, 72.3, 67.2, 67.2, 63.8, 61.2, 26.7, 26.6, 26.4, 26.3; **HRMS (TOF, MS, ES+):** m/z calcd. for $C_{25}H_{38}N_3O_9$: 524.2603; found: 524.2591 ($M+H^+$).

4-((((3a*R*,5*S*,6*S*,6a*R*)-6-(Allyloxy)tetrahydro-2,2-dimethylfuro[2,3-*d*][1,3]dioxol-5-yl)methoxy)methyl)-1-(((3a*R*,5*R*,6*S*,6a*R*)-6-(allyloxy)tetrahydro-2,2-dimethylfuro[3,2-*d*][1,3]dioxol-5-yl)methyl)-1*H*-1,2,3-triazole (3d): Following the general procedure A, the title compound (**3d**) was obtained as yellow syrup (Yield 90%); $\mathbf{R}_f$ 0.54 (50:50, ethyl acetate:hexane); **^{1}H NMR (600 MHz, $CDCl_3$):** δ 7.66 (s, 1H), 5.94 (d, *J* = 3.7 Hz, 1H), 5.93 – 5.85 (m, 3H), 5.35 (dd, *J* = 17.2, 1.8 Hz, 1H), 5.28-5.22 (m, 2H), 5.18 (dd, *J* = 10.7, 1.8 Hz, 1H), 4.72 – 4.65 (m, 3H), 4.62 (d, *J* = 3.7 Hz, 1H), 4.58 – 4.50 (m, 2H), 4.51 (dd, *J* = 13.5, 7.2 Hz, 1H), 4.22 – 4.16 (m, 2H), 4.09 – 4.03 (m, 2H), 4.02 – 3.98 (m, 1H), 3.92 (d, *J* = 12.8 Hz, 2H), 3.71 (dd, *J* = 6.30, 1.77 Hz, 2H), 1.46 (s, 3H), 1.41 (s, 3H), 1.30 (s, 3H), 1.28 (s, 3H); **^{13}C NMR (151 MHz, $CDCl_3$):** δ 145.0, 133.8, 133.5, 123.8, 118.3, 117.7, 112.7, 112.1, 105.7, 105.2, 84.9, 83.6, 83.0, 82.1, 81.6, 78.8, 71.0, 70.6, 70.5, 64.8, 49.2, 27.0, 26.7, 26.3, 26.2; **HRMS (TOF, MS, ES+):** m/z calcd. for $C_{25}H_{38}N_3O_9$: 524.2603; found: 524.2596 ($M+H^+$).

4-(((*R*)-2-((3a*R*,5*R*,6*S*,6a*R*)-6-(Allyloxy)tetrahydro-2,2-dimethylfuro[3,2-*d*][1,3]dioxol-5-yl)-2-(methoxymethoxy)ethoxy)methyl)-1-(((3a*R*,5*R*,6*S*,6a*R*)-6-(allyloxy)-tetrahydro-2,2-dimethylfuro[2,3-*d*][1,3]dioxol-5-yl)methyl)-1*H*-1,2,3-triazole (3e): Following the general procedure A, the title compound (**3e**) was obtained as syrup (Yield 76%); $\mathbf{R}_f$ 0.70 (70:30, ethyl acetate:hexane); **^{1}H NMR (600 MHz, CDCl$_3$):** δ 7.68 (s, 1H), 5.92 (d, *J* = 3.7 Hz, 1H), 5.84 (d, *J* = 3.7 Hz, 1H), 5.92 – 5.85 (m, 2H), 5.30 (t, *J* = 17.2 Hz, 2H), 5.24 (d, *J* = 10.3 Hz, 1H), 5.18 (d, *J* = 10.3 Hz, 1H), 4.73 – 4.63 (m, 5H), 4.58 (d, *J* = 3.6 Hz, 2H), 4.50 (dd, *J* = 14.3, 7.2 Hz, 1H), 4.27 (dd, *J* = 9.2, 2.4 Hz, 1H), 4.16 (td, *J* = 13.6, 5.4 Hz, 2H), 4.03 – 3.96 (m, 3H), 3.95 – 3.88 (m, 3H), 3.70 (dd, *J* = 10.3, 4.6 Hz, 1H), 3.33 (s, 3H), 1.45 (s, 3H), 1.40 (s, 3H), 1.28 (s, 3H), 1.27 (s, 3H); **^{13}C NMR (151 MHz, CDCl$_3$):** δ 145.4, 133.9, 133.5, 123.6, 118.2, 117.4, 112.0, 111.6, 105.2, 105.0, 97.0, 82.1, 81.6, 81.5, 81.5, 78.8, 78.5, 74.0, 71.0, 71.0, 70.7, 65.0, 55.7, 48.9, 26.7, 26.7, 26.3, 26.2; **HRMS (TOF, MS, ES+):** m/z calcd. for $C_{28}H_{43}N_3O_{11}$: 598.2970; found: 598.2959 (M+H$^+$).

4-(((3a*R*,5*R*,6*S*,6a*R*)-5-((Allyloxy)methyl)-dihydro-2,2-dimethyl-5*H*-furo[2,3-*d*][1,3]dioxol-6-yloxy)methyl)-1-((*R*)-2-((3a*R*,5*R*,6*S*,6a*R*)-6-(allyloxy)tetrahydro-2,2-dimethylfuro[3,2-*d*][1,3]dioxol-5-yl)-2-(methoxymethoxy)ethyl)-1*H*-1,2,3-triazole (3f): Following the general procedure A, the title compound (**3f**) was obtained as yellow syrup (Yield 78%); $\mathbf{R}_f$ 0.50 (50:50, ethyl acetate:hexane); **^{1}H NMR (600 MHz, CDCl$_3$):** δ 7.67 (s, 1H), 5.87-5.80 (m, 4H), 5.23 (m, 2H), 5.16 (d, *J* = 10.3 Hz, 1H), 5.11 (d, *J* = 10.3 Hz, 1H), 4.82 (d, *J* = 14.8 Hz, 1H), 4.70 (d, J = 12.9 Hz, 1H), 4.65-4.53 (m, 5H), 4.44 (dd, *J* = 14.6, 5.5 Hz, 1H), 4.46 (dd, *J* = 14.5, 5.1 Hz, 1H), 4.31 (m, 1H), 4.21 (m, 1H), 4.11 (dd, *J* = 12.9, 5.3 Hz, 2H), 4.01-3.85 (m, 6H), 3.57-3.64 (m, 2H), 3.30 (s, 3H), 1.42 (s, 3H), 1.37 (s, 3H), 1.26 (s, 3H), 1.25 (s, 3H); **^{13}C NMR (151 MHz, CDCl$_3$):** δ 144.2, 134.6, 133.5, 124.0, 117.9, 117.1, 111.9, 111.6, 105.0, 104.9, 97.1, 82.3, 81.9,

81.5, 81.1, 79.0, 78.9, 73.3, 72.3, 70.8, 67.3, 63.7, 56.1, 51.8, 26.7, 26.6, 26.3, 26.2; **HRMS (TOF, MS, ES+):** m/z calcd. for $C_{28}H_{44}N_3O_{11}$: 598.2970; found: 598.2968 ($M+H^+$).

4-(((*R*)-2-((3a*R*,5*R*,6*S*,6a*R*)-6-(Allyloxy)tetrahydro-2,2-dimethylfuro[2,3-*d*][1,3]dioxol-5-yl)-2-(methoxymethoxy)ethoxy)methyl)-1-((*R*)-2-((3a*R*,5*R*,6*S*,6a*R*)-6-(allyloxy)tetrahydro-2,2-dimethylfuro[3,2-*d*][1,3]dioxol-5-yl)-2-(methoxymethoxy)ethyl)-1*H*-1,2,3-triazole (3g): Following the general procedure A, the title compound (**3g**) was obtained as yellow syrup (Yield 92%); **R*f*** 0.59 (50:50, ethyl acetate:hexane); **^{1}H NMR (600 MHz, CDCl$_3$):** δ 7.70 (s, 1H), 5.86-5.81 (m, 4H), 5.23 (dd, *J* = 17.2, 9.0 Hz, 2H), 5.14 (dd, *J* = 16.5, 10.3 Hz, 2H), 4.78 (d, *J* = 14.3 Hz, 1H), 4.69-4.64 (m, 3H), 4.61 (d, *J* = 12.7 Hz, 1H), 4.55-4.54 (m, 2H), 4.51 (d, *J* = 3.7 Hz, 1H), 4.47 (d, *J* = 7.9 Hz, 1H), 4.43 (dd, *J* = 14.6, 6.23 Hz, 1H), 4.24 - 4.21 (m, 2H), 4.12 - 4.07 (m, 2H), 3.95 - 3.91 (m, 4H), 3.87 (m, 2H), 3.82 (d, *J* = 10.5 Hz, 2H), 3.62 (dd, *J* = 10.5, 4.5 Hz, 1H), 3.30 (s, 3H), 3.25 (s, 3H), 1.42 (s, 3H), 1.39 (s, 3H), 1.26 (s, 6H); **^{13}C NMR (151 MHz, CDCl$_3$):** δ 144.9, 133.9, 133.6, 124.0, 117.9, 117.4, 111.9, 111.6, 105.0, 104.9, 97.1, 97.0, 81.6, 81.5, 81.4, 81.2, 79.2, 78.5, 73.9, 73.6, 70.8, 70.7, 64.9, 56.0, 55.7, 51.9, 26.7, 26.6, 26.3; **HRMS (TOF, MS, ES+):** m/z calcd. for $C_{31}H_{49}N_3O_{13}$: 672.3338; found: 672.3330 ($M+H^+$).

1-[(2*R*)-2-[(3a*R*,5*R*,6*S*,6a*R*)-2,2-Dimethyl-6-(prop-2-en-1-yloxy)-tetrahydro-2*H*-furo[2,3-d][1,3]dioxol-5-yl]-2-(methoxymethoxy)ethyl]-4-({[(3a*R*,5*R*,6*S*,6a*R*)-5-[(1*R*)-2-[(*tert*-butyldimethylsilyl)oxy]-1-(prop-2-en-1-yloxy)ethyl]-2,2-dimethyl-tetrahydro-2*H*-furo[2,3-*d*][1,3]dioxol-6-yl]oxy}methyl)-1*H*-1,2,3-triazole (**3h):** Following the general procedure A, the title compound (**3h)** was obtained as syrup (Yield 91%); **R*f*** 0.44 (40:60, ethyl acetate:hexane); **^{1}H NMR (600 MHz, CDCl$_3$):** δ 7.69 (s, 1H), 5.90 (d, *J* = 3.7 Hz, 1H), 5.91-5.82 (m, 2H), 5.82 (d, *J* = 3.7 Hz, 1H), 5.30-5.19 (m, 3H), 5.08 (d, *J* = 10.4 Hz, 1H), 4.85 (dd, *J* = 14.4, 2.6 Hz, 1 H), 4.73 (d, *J* = 11.9 Hz, 1H), 4.67 (d , *J* =11.9 Hz, 1H), 4.61 (d, *J* = 6.9 Hz, 1H), 4.57 (m, 3H),

4.47 (dd, *J* = 14.1, 5.7 Hz, 1H), 4.29 - 4.24 (m, 2H), 4.15 (m, 2H), 4.07 (d, *J* = 3.0 Hz, 1H), 4.02 (dd, *J* = 12.2, 5.02 Hz, 2H), 3.98 - 3.92 (m, 3H), 3.89 (d, *J* = 2.8 Hz, 1H), 3.69 (m, 1H), 3.33 (s, 3H), 1.44 (s, 3H), 1.40 (s, 3H), 1.29 (s, 3H), 1.28 (s, 3H), 0.87 (s, 9H), 0.03 (s, 6H); **^{13}C NMR (151 MHz, $CDCl_3$):** δ 144.2, 135.4, 133.5, 124.0, 118.0, 116.2, 112.0, 111.7, 105.1, 105.0, 97.1, 82.0, 81.9, 81.5, 81.2, 78.9, 78.6, 76.7, 73.4, 71.8, 70.9, 64.0, 63.8, 56.2, 51.8, 26.6, 26.3, 26.3, 25.9, 18.2, -5.4, -5.5; **HRMS (TOF, MS, ES+):** m/z calcd. for $C_{35}H_{60}N_3O_{12}Si$: 742.3941; found:742.3931 ($M+H^+$).

(*R*)-2-(4-(((3a*R*,5*R*,6*S*,6a*R*)-5-((Allyloxy)methyl)-dihydro-2,2-dimethyl-5*H*-furo[2,3-*d*][1,3]-dioxol-6-yloxy)methyl)-1*H*-1,2,3-triazol-1-yl)-1-((3a*R*,5*R*,6*S*,6a*R*)-6-(allyloxy)-tetrahydro-2,2-dimethylfuro[3,2-*d*][1,3]dioxol-5-yl)ethanol (3i): Following the general procedure A, the title compound (**3i**) was obtained as yellow syrup (Yield 85%); **R_f** 0.64 (70:30, ethyl acetate:hexane); **^{1}H NMR (600 MHz, $CDCl_3$):** δ 7.65 (s, 1H), 5.88 (d, *J* = 3.7 Hz, 1H), 5.85 (d, *J* = 3.7 Hz, 1H), 5.89 – 5.81 (m, 2H), 5.25 (t, *J* = 15.6 Hz, 2H), 5.16 (dd, *J* = 18.5, 10.4 Hz, 2H), 4.71 (d, *J* = 12.6 Hz, 2H), 4.62 (d, *J* = 12.6 Hz, 1H), 4.56 (dd, *J* = 17.3, 3.8 Hz, 2H), 4.37 (dd, *J* = 14.0, 7.3 Hz, 1H), 4.35 – 4.29 (m, 2H), 4.13 (dd, *J* = 13.0, 5.8 Hz, 1H), 4.05 – 3.94 (m, 5H), 3.86 (dd, *J* = 8.7, 3.2 Hz, 1H), 3.65 (dd, *J* = 9.8, 6.4 Hz, 1H), 3.60 (dd, *J* = 9.8, 6.4 Hz, 1H), 3.47 (bs, 1H), 1.44 (s, 3H), 1.40 (s, 3H), 1.28 (s, 3H), 1.27 (s, 3H); **^{13}C NMR (151 MHz, $CDCl_3$):** δ 144.4, 134.6, 133.8, 124.3, 118.2, 117.3, 112.0, 111.8, 105.3, 105.0, 82.4, 82.4, 82.0, 81.2, 80.3, 79.0, 72.4, 71.4, 67.7, 67.2, 63.7, 54.0, 26.8, 26.8, 26.4, 26.3; **HRMS (TOF, MS, ES+):** m/z calcd. for $C_{26}H_{40}N_3O_{10}$: 554.2708; found: 554.2690 [$M+H^+$].

(*R*)-2-(4-((((3a*R*,5*R*,6*S*,6a*R*)-6-(Allyloxy)tetrahydro-2,2-dimethylfuro[2,3-*d*][1,3]dioxol-5-yl)methoxy)methyl)-1*H*-1,2,3-triazol-1-yl)-1-((3a*R*,5*R*,6*S*,6a*R*)-6-(allyloxy)tetrahydro-2,2-dimethylfuro[3,2-*d*][1,3]dioxol-5-yl)ethanol (3j): Following the general procedure A, the title

compound (**3j**) was obtained as yellow syrup (Yield 87%); **R*f*** 0.60 (70:30, ethyl acetate:hexane); **^{1}H NMR (600 MHz, CDCl$_3$):** δ 7.66 (s, 1H), 5.88 (d, *J* = 3.7 Hz, 1H), 5.87 (d, *J* = 3.7 Hz, 1H), 5.87 – 5.77 (m, 2H), 5.27 – 5.22 (m, 2H), 5.18 – 5.14 (m, 2H), 4.70 (dd, *J* = 14.0, 2.1 Hz, 1H), 4.65 (d, *J* = 12.7 Hz, 1H), 4.60 (d, *J* = 12.7 Hz, 1H), 4.54 (d, *J* = 3.7 Hz, 1H), 4.50 (d, *J* = 3.7 Hz, 1H), 4.37 (dd, *J* = 14.0, 7.6 Hz, 1H), 4.33 – 4.29 (m, 2H), 4.13 (dd, *J* = 12.8, 5.6 Hz, 1H), 4.07 (dd, *J* = 12.8, 5.6 Hz, 1H), 4.04 – 3.99 (m, 2H), 3.94 (dd, *J* = 12.8, 5.6 Hz, 1H), 3.88 (dd, *J* = 8.5, 3.1 Hz, 1H), 3.85 (d, *J* = 3.1 Hz, 1H), 3.78 (dd, *J* = 10.2, 5.5 Hz, 1H), 3.73 (dd, *J* = 10.2, 6.4 Hz, 1H), 3.50 (bs, 1H), 1.43 (s, 3H), 1.40 (s, 3H), 1.28 (s, 3H), 1.27 (s, 3H); **^{13}C NMR (151 MHz, CDCl$_3$):** δ 144.7, 134.0, 133.8, 124.3, 118.0, 117.5, 112.0, 111.6, 105.2, 105.0, 82.4, 81.7, 81.2, 80.2, 79.1, 71.3, 71.0, 68.0, 67.6, 64.8, 53.9, 26.8, 26.8, 26.3, 26.3; **HRMS (TOF, MS, ES+):** m/z calcd. for $C_{26}H_{40}N_3O_{10}$: 554.2708; found: 554.2695 [M+H$^+$].

(*R*)-2-((3a*R*,5*R*,6*S*,6a*R*)-6-((1-(((3a*R*,5*R*,6*S*,6a*R*)-6-(Allyloxy)tetrahydro-2,2-dimethylfuro-[2,3-*d*][1,3]dioxol-5-yl)methyl)-1*H*-1,2,3-triazol-4-yl)methoxy)dihydro-2,2-dimethyl-5*H*-furo[2,3-*d*][1,3]dioxol-5-yl)-2-(allyloxy)ethanol (3k): Following the general procedure A, the title compound (**3k**) was obtained as yellow syrup (Yield 95%); **R*f*** 0.49 (70:30, ethyl acetate:hexane); **^{1}H NMR (600 MHz,CDCl$_3$):** δ 7.64 (s, 1H), 5.93 (d, *J* = 3.7 Hz, 1H), 5.86 (d, *J* = 3.7 Hz, 1H), 5.92 – 5.84 (m, 2H), 5.30 (dd, *J* = 17.3, 1.6 Hz, 1H), 5.25 (s, 1H), 5.24 (dd, *J* = 5.3, 1.5 Hz, 1H), 5.14 (dd, *J* = 10.5, 1.3 Hz, 1H), 4.77 (d, *J* = 12.0 Hz, 1H), 4.70 (dd, *J* = 13.3, 3.1 Hz, 1H), 4.65 (d, *J* = 12.1 Hz, 1H), 4.60 (dd, *J* = 16.3, 3.7 Hz, 1H, 4.55 – 4.47 (m, 2H), 4.23 (dd, *J* = 8.2, 3.2 Hz, 1H), 4.17 (dd, *J* = 12.8, 5.3 Hz, 1H), 4.14 (dd, *J* = 12.8, 5.3 Hz, 1H), 4.08 (d, *J* = 3.2 Hz, 1H), 4.00 (td, *J* = 13.2, 5.3 Hz, 2H), 3.92 (d, *J* = 3.2 Hz, 1H), 3.87 – 3.84 (m, 1H), 3.73 – 3.69 (m, 2H), 1.46 (s, 3H), 1.40 (s, 3H), 1.29 (s, 3H), 1.28 (s, 3H); **^{13}C NMR (151 MHz, CDCl$_3$):** δ 144.3, 134.8, 133.4, 123.7, 118.3, 116.9, 112.1, 111.8, 105.2, 105.0, 82.1, 82.0, 81.7,

81.7, 79.5, 78.8, 75.6, 71.0, 71.0, 63.5, 61.9, 49.3, 26.7, 26.7, 26.2, 26.1; **HRMS (TOF, MS, ES+):** m/z calcd. for $C_{26}H_{40}N_3O_{10}$: 554.2708; found: 554.2693 (M+H$^+$).

(*R*)-2-(4-(((*R*)-2-((3a*R*,5*R*,6*S*,6a*R*)-6-(Allyloxy)tetrahydro-2,2-dimethylfuro[2,3-*d*][1,3]-dioxol-5-yl)-2-(methoxymethoxy)ethoxy)methyl)-1*H*-1,2,3-triazol-1-yl)-1-((3a*R*,5*R*,6*S*,6a*R*)-6-(allyloxy)tetrahydro-2,2-dimethylfuro[3,2-*d*][1,3]dioxol-5-yl)ethanol (3l): Following the general procedure A, the title compound (**3l**) was obtained as syrup (Yield 77%); **R*f*** 0.65 (70:30, ethyl acetate:hexane); **^{1}H NMR (600 MHz, CDCl$_3$):** δ 7.74 (s, 1H), 5.88 (d, *J* = 3.7 Hz, 1H), 5.86 – 5.81 (m, 2H), 5.81 (d, *J* = 3.7 Hz, 1H), 5.27 (dd, *J* = 17.9, 1.6 Hz, 2H), 5.16 (t, *J* = 8.9 Hz, 2H), 4.74 (d, *J* = 12.7 Hz, 1H), 4.72 – 4.66 (m, 3H), 4.60 (d, *J* = 12.7 Hz, 1H), 4.55 – 4.52 (m, 2H), 4.37 – 4.32 (m, 1H), 4.34 (dd, *J* = 13.6, 7.7 Hz, 1H), 4.29 (dd, *J* = 8.7, 2.9 Hz, 2H), 4.14 – 4.08 (m, 2H), 4.03 (dd, *J* = 10.3, 3.4 Hz, 2H), 3.96 – 3.92 (m, 3H), 3.90 (d, *J* = 3.4 Hz, 1H), 3.85 – 3.82 (m, 1H), 3.64 (dd, *J* = 10.3, 3.4 Hz, 1H), 3.59 (bs, 1H), 3.33 (s, 3H), 1.45 (s, 3H), 1.42 (s, 3H), 1.28 (s, 6H); **^{13}C NMR (151 MHz, CDCl$_3$):** δ 133.9, 133.8, 124.2, 117.9, 117.4, 111.9, 111.7, 105.2, 104.9, 97.0, 82.3, 81.6, 81.4, 81.2, 80.4, 78.3, 74.0, 71.3, 70.7, 70.3, 67.8, 64.8, 55.7, 54.0, 26.8, 26.7, 26.3, 26.3; **HRMS (TOF, MS, ES+):** m/z calcd. for $C_{29}H_{45}N_3O_{12}Na$: 650.2895; found: 650.2899 (M+Na$^+$).

(2*R*)-2-[(3a*R*,5*R*,6*S*,6a*R*)-6-({1-[(2*R*)-2-[(3a*R*,5*R*,6*S*,6a*R*)-2,2-Dimethyl-6-(prop-2-en-1-yloxy)tetrahydro-2*H*-furo[2,3-*d*][1,3]dioxol-5-yl]-2-hydroxyethyl]-1*H*-1,2,3-triazol-4-yl} methoxy)-2,2-dimethyl-tetrahydro-2*H*-furo[2,3-*d*][1,3]dioxol-5-yl]-2-(prop-2-en-1-yloxy)ethan-1-ol (3m): Following the general procedure A, the title compound (**3m**) was obtained as yellow syrup (Yield 67%); **R*f*** 0.60 (70:30, ethyl acetate:hexane); **^{1}H NMR (600 MHz, CDCl$_3$):** δ 7.65 (s, 1H), 5.88 (d, *J* = 3.7 Hz, 1H), 5.82 (d, *J* = 4.1 Hz, 1H), 5.87 – 5.80 (m, 2H), 5.25 (d, *J* = 17.6 Hz, 1H), 5.22 (d, *J* = 17.6 Hz, 1H), 5.16 (d, *J* = 10.5 Hz, 1H), 5.10 (d, *J* =

10.5 Hz, 1H), 4.75 – 4.69 (m, 2H), 4.63 – 4.59 (m, 2H), 4.54 (d, *J* = 3.7 Hz, 1H), 4.37 (dd, *J* = 14.4, 7.2 Hz, 1H), 4.29 (t, *J* = 7.2 Hz, 1H), 4.19 (dd, *J* = 8.6, 2.9 Hz, 1H), 4.14 – 4.08 (m, 2H), 4.05 (d, *J* = 2.9 Hz, 1H), 4.03 – 3.95 (m, 3H), 3.87 (dd, *J* = 8.7, 3.3 Hz, 1H), 3.85 – 3.81 (m, 1H), 3.71 – 3.64 (m, 2H), 3.52 (bs, 1H), 2.37 (bs, 1H), 1.44 (s, 3H), 1.40 (s, 3H), 1.28 (s, 6H); **^{13}C NMR (151 MHz, $CDCl_3$):** δ 143.9, 134.8, 133.7, 124.4, 118.1, 116.8, 112.0, 111.9, 105.2, 105.0, 82.2, 81.8, 81.7, 81.1, 80.2, 79.4, 75.7, 71.2, 71.0, 67.6, 63.3, 61.9, 53.9, 26.7, 26.7, 26.3, 26.2; **HRMS (TOF, MS, ES+):** m/z calcd for $C_{27}H_{42}N_3O_{11}$ 584.2814; found: 584.2803.

Synthesis of RCM products

General procedure B: Ring-closing metathesis (RCM)

To a solution of triazole (1.0 eq) in anhydrous dichloromethane or ethyl acetate (0.001M) was added *Grubbs second-generation catalyst* (5 mol %) and the solution was stirred for 2-3 hours at 50 °C for dichloromethane or at 75 °C for ethyl acetate. Reaction was monitored by TLC (after 1 and 2 hours) and if reaction was stalled then additional catalyst 3–5 mol % was loaded until the completion of the reaction. After completion (TLC), reaction mixture was concentrated under reduced pressure and residue was purified by silica gel column chromatography using ethyl acetate and petroleum ether as mobile phase. The *E:Z* product ratio was determined by ^{1}H NMR.

(3*R*,5*R*,9*R*,10*S*,13*E*,17*S*,18*R*,22*R*,24*R*)-7,7,20,20-Tetramethyl-4,6,8,11,16,19,21,23,26-nonaoxa-1,29,30-triazahexacyclo[26.2.1.0^{3,10}.0^{5,9}.0^{17,24}.0^{18,22}]hentriaconta-13,28(31),29-triene (4a)

Following the general procedure B, the title compound (**4a**) was obtained as yellow syrup (Yield dichloromethane- 63% and ethyl acetate- 84%) (Product ratio *E:Z*; 96:4); **R_f** 0.55 (5:95, dichloromethane:methanol); **^{1}H NMR (600 MHz, $CDCl_3$)**: δ 7.54 (s, 1H), 5.92 (d, *J* = 3.7 Hz,

1H), 5.85 (d, *J* = 3.7 Hz, 1H), 5.70 – 5.60 (m, 2H), 4.84 (d, *J* = 13.4 Hz, 1H), 4.73 (td, *J* = 7.0, 3.67 Hz, 1H), 4.63 (d, *J* = 7.0 Hz, 2H), 4.60 (d, *J* = 13.2 Hz, 1H), 4.55 (d, *J* = 3.7 Hz, 1H), 4.52 (d, *J* = 3.7 Hz, 1H), 4.39 – 4.35 (m, 1H), 4.23 (dd, *J* = 12.6, 4.6 Hz, 1H), 4.18 (d, *J* = 12.6 Hz, 1H), 3.92 – 3.87 (m, 2H), 3.78 (dd, *J* = 12.6, 6.4 Hz, 1H), 3.74 – 3.69 (m, 2H), 3.58 (dd, *J* = 8.3, 4.5 Hz, 1H), 1.50 (s, 3H), 1.48 (s, 3H), 1.31 (s, 3H), 1.30 (s, 3H); **^{13}C NMR (151 MHz, $CDCl_3$)**: δ 144.5, 132.1, 126.5, 123.5, 112.2, 111.8, 105.0, 104.9, 82.4, 81.5, 81.1, 78.2, 78.1, 77.8, 69.3, 68.7, 65.6, 65.0, 46.7, 26.8, 26.3, 26.2; **HRMS (TOF, MS, ES+):** m/z calcd. for $C_{23}H_{34}N_3O_9$: 496.2290; found: 496.2269 ($M+H^+$).

(3*R*,5*R*,9*R*,10*S*,13*E*,18*S*,20*S*,24*S*,25*R*)-7,7,22,22-Tetramethyl-4,6,8,11,16,19,21,23,26-nona-oxa-1,29,30-triazahexacyclo[26.2.1.0^{3,10}.0^{5,9}.0^{18,25}.0^{20,24}]hentriaconta-13,28(31),29-triene (4b)

Following the general procedure B, the title compound (**4b**) was obtained as yellow syrup (Yield dichloromethane- 85% and ethyl acetate- 94%) (Product ratio: *E:Z*; 97:3).

R_f 0.40 (70:30, ethyl acetate:hexane); **^{1}H NMR (600 MHz, $CDCl_3$):** δ 7.57 (s, 1H), 5.91 (d, *J* = 3.7 Hz, 1H), 5.90 (d, *J* = 3.7 Hz, 1H), 5.69 – 5.65 (m, 2H), 4.86 (d, *J* = 13.0 Hz, 1H), 4.75 (td, *J* = 7.2, 3.3 Hz, 1H), 4.70 (d, *J* = 3.7 Hz, 1H), 4.63 (d, *J* = 13.1 Hz, 1H), 4.61 (m, 3H), 4.58 (d, *J* = 3.7 Hz, 1H), 4.33 – 4.29 (m, 1H), 4.22 (dd, *J* = 13.5, 4.1 Hz, 1H), 4.08 (d, *J* = 13.1 Hz, 1H), 4.00 (d, *J* = 3.1 Hz, 1H), 3.84 (dd, *J* = 13.1, 4.1 Hz, 1H), 3.78 – 3.72 (m, 2H), 3.70 (t, *J* = 8.7 Hz, 1H), 3.55 (dd, *J* = 8.8, 4.9 Hz, 1H), 1.49 (s, 3H), 1.48 (s, 3H), 1.32 (s, 3H), 1.31 (s, 3H); **^{13}C NMR (151 MHz, $CDCl_3$):** δ 144.2, 132.4, 126.9, 123.8, 112.2, 111.8, 105.0, 104.9, 81.6, 81.5, 80.2, 78.5, 78.0, 77.9, 70.4, 69.0, 66.2, 62.9, 46.8, 26.8, 26.7, 26.2, 26.2; **HRMS (TOF, MS, ES+):** m/z calcd. for $C_{23}H_{34}N_3O_9$: 496.2290, found: 496.2279 ($M+H^+$).

(2*R*,3*R*,7*R*,9*S*,13*E*,18*R*,20*R*,24*R*,25*S*)-5,5,22,22-tetramethyl-4,6,8,11,16,19,21,23,26-nona oxa-1,29,30-triazahexacyclo[26.2.1.0^{2,9} .0^{3,7} .0^{18} ,^{25} .0^{20} ,^{24}]hentriaconta-13,28(31),29-triene 4c:

Following the general procedure B, the title compound (**4c**) was obtained as syrup (Yield dichloromethane- 88% and ethyl acetate- 94%) (Product ratio: *E:Z*; 94:6); $\mathbf{R_{\mathit{f}}}$ 0.55 (70:30, ethyl acetate:hexane); **^{1}H NMR (600 MHz, $CDCl_3$):** δ 7.71 (s, 1H), 5.96 (s, 1H), 5.93 (s, 1H), 5.46 (td, *J* = 15.5, 5.7 Hz, 1H), 5.37 (td, *J* = 15.7, 5.7, Hz, 1H), 5.97 (dd, *J* = 10.15, 3.67 Hz, 1H), 4.88 (t, *J* = 3.7 Hz, 1H), 4.83 (d, *J* = 12.9 Hz, 1H), 4.65 (s, 1H), 4.64 – 4.57 (m, 2H), 4.30 (m, 1H), 4.24 (s, 1H), 4.06 (d, *J* = 12.7 Hz, 1H), 3.90 (dd, *J* = 12.9, 5.6 Hz, 2H), 3.86 (dd, *J* = 13.0, 6.6 Hz, 1H), 3.66 (dd, *J* = 10.7, 5.1 Hz, 1H), 3.51 (d, *J* = 10.3, 3.3 Hz, 1H), 3.37 – 3.33 (m, 1H), 3.30 – 3.26 (m, 1H), 1.60 (s, 3H), 1.48 (s, 3H), 1.32 (s, 6H); **^{13}C NMR (151 MHz, $CDCl_3$):** δ 144.4, 131.0, 128.6, 123.0, 113.5, 111.8, 105.2, 104.3, 82.4, 81.3, 78.8, 78.7, 76.1, 70.9, 70.5, 67.3, 66.7, 63.1, 62.5, 26.8, 26.5, 26.3, 26.3; **HRMS (TOF, MS, ES+):** m/z calcd. for $C_{23}H_{34}N_3O_9$: 496.2290, found: 496.2282 ($M+H^+$).

(3*R*,5*R*,9*R*,10*S*,13*E*,17*S*,18*R*,22*R*,24*S*)-7,7,20,20-Tetramethyl-4,6,8,11,16,19,21,23,26-nona oxa-1,29,30-triazahexacyclo[26.2.1.0^{3,10}.0^{5,9}.0^{17,24}.0^{18,22}]hentriaconta-13,28(31),29-triene (4d):

Following the general procedure B, the title compound (**4d**) was obtained as syrup (Yield dichloromethane- 70% and ethyl acetate- 90%) (Product ratio: *E:Z*; 97:3); $\mathbf{R_{\mathit{f}}}$ 0.58 (70:30, ethyl acetate:hexane); **^{1}H NMR (600 MHz, $CDCl_3$):** δ 7.62 (s, 1H), 5.95 (d, *J* = 3.7 Hz, 1H), 5.85 (d, *J* = 3.7 Hz, 1H), 5.66 (td, *J* = 15.7, 5.9 Hz, 1H), 5.59 (td, *J* = 15.7, 5.9 Hz, 1H), 4.80 – 4.74 (m, 2H), 4.73 – 4.69 (m, 1H), 4.62 – 4.58 (m, 2H), 4.56 – 4.51 (m, 2H), 4.27 (dd, *J* = 12.6, 5.2 Hz,

1H), 4.16 (d, *J* = 2.3 Hz, 1H), 4.13 – 4.10 (m, 1H), 4.07 (dd, *J* = 13.8, 5.5 Hz, 1H), 4.01 (dd, *J* = 13.8, 5.5 Hz, 1H), 3.78 (dd, *J* = 12.3, 6.8 Hz, 1H), 3.75 (dd, *J* = 9.2, 4.3 Hz, 1H), 3.70 (d, *J* = 8.9 Hz, 1H), 3.68 (d, *J* = 2.3 Hz, 1H), 1.47, 1.47 (2s, 6H), 1.31(s, 3H), 1.30 (s, 3H); **^{13}C NMR (151 MHz, $CDCl_3$):** δ 145.31, 130.27, 130.01, 123.91, 112.84, 112.13, 105.61, 105.05, 85.56, 82.65, 81.07, 80.81, 80.68, 77.70, 70.01, 69.50, 68.34, 65.23, 47.15, 27.26, 26.77, 26.33, 26.23; **HRMS (TOF, MS, ES+):** m/z calcd. for $C_{23}H_{34}N_3O_9$: 496.229; found: 496.2280 ($M+H^+$).

(3*R*,5*R*,9*R*,10*S*,13*E*,17*S*,18*R*,22*R*,24*R*,25*R*)-25-(Methoxymethoxy)-7,7,20,20-tetramethyl-4,6,8,11,16,19,21,23,27-nonaoxa-1,30,31-triazahexacyclo[27.2.1.0^{3,10}.0^{5,9}.0^{17,24}.0^{18,22}] dotriaconta-13,29(32),30-triene (4e):

Following the general procedure B, the title compound (**4e**) was obtained as yellow syrup (Yield dichloromethane- 88% and ethyl acetate- 40%) (Product ratio: *E:Z*; 96:4); **R_f** 0.40 (70:30, ethyl acetate:hexane); **^{1}H NMR (600 MHz, $CDCl_3$):** δ 7.62 (s, 1H), 5.91 (d, *J* = 3.7 Hz, 1H), 5.86 (d, *J* = 3.7 Hz, 1H), 5.63 (td, *J* = 15.0, 4.7 Hz, 1H), 5.43 (m, 1H), 4.87 (d, *J* = 13.5 Hz, 1H), 4.85 – 4.82 (m, 1H), 4.81 (d, *J* = 7.2 Hz, 1H), 4.71 (d, *J* = 7.0 Hz, 1H), 4.64 (dd, *J* = 13.2, 4.2 Hz, 1H), 4.58 (d, *J* = 3.7 Hz, 1H), 4.56 – 4.52 (m, 1H), 4.50 (d, *J* = 13.2 Hz, 1H), 4.46 (d, *J* = 3.8 Hz, 1H), 4.34 (dd, *J* = 3.4, 1.9 Hz, 1H), 4.25 (dd, *J* = 12.8, 4.5 Hz, 1H), 4.13 (d, *J* = 13.2 Hz, 1H), 4.07 (d, *J* = 8.2 Hz, 1H), 3.92 (dd, *J* = 10.7, 1.7 Hz, 1H), 3.88 – 3.82 (m, 3H), 3.80 (d, *J* = 3.7 Hz, 1H), 3.67 (dd, *J* = 10.7, 8.5 Hz, 1H), 3.37 (s, 3H), 1.47 (s, 3H), 1.44 (s, 3H), 1.30 (s, 3H), 1.28 (s, 3H); **^{13}C NMR (151 MHz, $CDCl_3$):** δ 145.4, 131.1, 125.5, 124.2, 112.0, 111.5, 104.9, 104.8, 96.6, 83.9, 81.5, 81.3, 80.9, 78.8, 77.6, 75.3, 72.4, 69.2, 68.7, 64.9, 55.5, 47.0, 26.8, 26.8, 26.3, 26.2; **HRMS (TOF, MS, ES+):** m/z calcd. for $C_{26}H_{40}N_3O_{11}$: 570.2657; found: 570.2650 ($M+H^+$).

(4*S*,5*R*,9*R*,11*R*,15*E*,19*S*,20*R*,24*R*,26*R*,27*R*)-27-(Methoxymethoxy)-7,7,22,22-tetramethyl-3,6,8,10,13,18, 21,23,25-nonaoxa-29,30,31-triazahexacyclo[27.2.1.0^{4,11}.0^{5,9}.0^{19,26}.0^{20,24}] dotriaconta-1(32),15,30-triene (4f):

Following the general procedure B, the title compound (**4f**) was obtained. (Yield dichloromethane- 83% and ethyl acetate- 39%) (Product ratio: *E*:*Z*; 88:12); **R_f** 0.35 (60:40, ethyl acetate:hexane); **^{1}H NMR (600 MHz, $CDCl_3$):** δ 7.65 (s, 1H), 5.91 (d, *J* = 3.67 Hz, 1H), 5.90 (d, *J* = 3.7 Hz, 1H), 5.85 (td, *J* = 15.7, 5.9 Hz, 1H), 5.77 (td, *J* = 15.7, 5.9 Hz, 1H), 4.85 (d, *J* = 13.7 Hz, 1H), 4.64-4.58 (m, 5H), 4.50 (d, *J* = 7.1 Hz, 1H), 4.32 (t, *J* = 3.8 Hz, 1H), 4.28 (d, *J* = 7.2 Hz, 2H), 4.25-4.21 (m, 2H), 4.14 (dd, *J* = 12.7, 6.0 Hz, 1H), 3.93 (d, *J* = 3.2 Hz, 1H), 3.89 (dd, *J* = 12.5, 5.9 Hz, 1H), 3.85 (d, *J* = 3.2 Hz, 1H), 3.81 (dd, *J* =12.2, 5.9 Hz, 1H), 3.65 (dd, *J* = 9.4 , 5.4 Hz, 1H), 3.48 (dd, *J* = 9.5, 7.2 Hz, 1H), 3.06 (s, 3H), 1.47 (s, 3H), 1.41 (s, 3H), 1.30 (s, 3H), 1.27 (s, 3H); **^{13}C NMR (151 MHz, $CDCl_3$):** δ 145.7, 129.4, 128.9, 121.9, 112.0, 111.7, 105.1, 105.0, 96.5, 83.4, 81.7, 81.2, 80.8, 80.4, 78.7, 76.2, 70.9, 69.8, 67.2, 62.8, 55.9, 52.9, 26.8, 26.7, 26.3, 26.2; **HRMS (TOF, MS, ES+):** m/z calcd. for $C_{26}H_{40}N_3O_{11}$: 570.2657; found:570.2657 (M+H$^+$).

(3*R*,4*R*,6*R*,10*R*,11*S*,14*E*,18*S*,19*R*,23*R*,25*R*,26*R*)-3,26-Bis(methoxymethoxy)-8,8,21,21-tetramethyl-5,7,9,12,17,20,22,24,28-nonaoxa-1,31,32-triazahexacyclo [28.2.1.0^{4,11}.0^{6,10}.0^{18,25}.0^{19,23}]tritriaconta-14,30(33),31-triene (4g):

Following the general procedure B, the title compound (**4g**) was obtained as yellow syrup (Yield dichloromethane- 77% and ethyl acetate- 92%) (Product ratio: *E*:*Z*; 90:10); **R_f** 0.40 (70:30, ethyl acetate:hexane); **^{1}H NMR (600 MHz, $CDCl_3$):** δ 7.65 (s, 1H), 5.93 (s, 1H), 5.72 (s, 3H), 4.93 (d, *J* = 14.1 Hz, 1H), 4.73 (dd, *J* = 5.5, 1.8 Hz, 1H), 4.68 (d, *J* = 14.1 Hz, 1H), 4.64-4.57 (m, 2H),

4.55-4.50 (m, 3H), 4.43 (d, *J* = 2.7 Hz, 1H), 4.41 (dd, *J* = 6.7, 1.6 Hz, 1H), 4.32-4.30 (m, 2H), 4.25 (d, *J* = 11.8 Hz, 1H), 4.21-4.18 (m, 1H), 4.06-4.02 (m, 2H), 3.97-3.91 (m, 3H), 3.84 (m, 2H), 3.74-3.68 (m, 2H), 3.32 (m, 3H), 3.07 (m, 3H), 1.46 (s, 3H), 1.41 (s, 3H), 1.29 (s, 3H), 1.24 (s, 3H); **^{13}C NMR (151 MHz, $CDCl_3$):** δ 147.0, 129.0, 127.1, 121.8, 111.9, 111.6, 105.2, 104.8, 96.9, 96.7, 82.5, 82.0, 81.5, 80.9, 80.8, 80.6, 75.9, 74.4, 73.0, 69.4, 68.6, 65.9, 55.7, 55.6, 52.9, 26.8, 26.7, 26.2; **HRMS (TOF, MS, ES+):** m/z calcd. for $C_{29}H_{46}N_3O_{13}$: 644.3025; found: 644.3025 (M+H$^+$).

(4*S*,5*R*,9*R*,11*R*,12*R*,15*E*,19*S*,20*R*,24*R*,26*R*,27*R*)-12-{[(*tert*-Butyldimethylsilyl)oxy]methyl}-27-(methoxymethoxy)-7,7,22,22-tetramethyl-3,6,8,10,13,18,21,23,25-nonaoxa-29,30,31-triazahexacyclo[27.2.1.0^{4,11}.0^{5,9}.0^{19,26}.0^{20,24}]dotriaconta-1(32),15,30-triene (4h):

Following the general procedure B, the title compound (**4h**) was obtained (Yield dichloromethane- 82% and ethyl acetate- 92%) (Product ratio: *E:Z*; 97:3); **R_f** 0.58 (55:45, ethyl acetate:hexane); **^{1}H NMR (600 MHz, $CDCl_3$):** δ 7.69 (s,1H), 5.95 (d, *J* = 3.3 Hz, 1H), 5.87 (d, *J* = 3.3 Hz, 1H), 5.75(td, *J* = 15.7, 4.5 Hz, 1H), 5.66 (td, *J* = 15.7, 4.6 Hz, 2H), 4.90 (d, *J* = 13.7 Hz, 1H), 4.76 (d, *J* = 14.5 Hz, 1H), 4.72 (d, *J* = 14.5 Hz, 1H), 4.69 (d, *J* = 5.6 Hz, 1H), 4.65 (d, *J* = 7.3 Hz, 1H), 4.60-4.55 (m, 2H), 4.51 (d, *J* = 7.3 Hz, 1H), 4.49 (s, 1H), 4.32-4.24 (m, 3H), 4.12 (dd, *J* = 8.6, 2.78 Hz, 1H), 4.01-3.96 (m, 4H), 3.83 (dd, *J* =12.4, 4.3 Hz, 1H), 3.75-3.69 (m, 2H), 3.16 (s, 3H), 1.48 (s, 3H), 1.42 (s, 3H), 1.31 (s, 3H), 1.28 (s, 3H), 0.87 (s, 9H), 0.03 (s, 6H); **^{13}C NMR (151 MHz, $CDCl_3$):** δ 144.5, 130.2, 127.0, 122.2, 112.0, 111.5, 105.2, 104.7, 96.0, 84.8, 81.5, 81.2, 80.5, 79.3, 78.3, 76.0, 70.1, 69.1, 63.4, 62.1, 55.6, 55.6, 52.7, 26.9, 26.6, 26.3, 26.2, 25.9, 18.2, -5.4, -5.5; **HRMS (TOF, MS, ES+):** m/z calcd. for $C_{33}H_{56}N_3O_{11}$:714.3628; found: 714.3619 (M+H$^+$).

(4*S*,5*R*,9*R*,11*R*,15*E*,19*S*,20*R*,24*R*,26*R*,27*R*)-7,7,22,22-Tetramethyl-3,6,8,10,13,18,21,23,25-nonaoxa-29,30,31-triazahexacyclo[27.2.1.0^{4,11}.0^{5,9}.0^{19,26}.0^{20,24}]dotriaconta-1(32),15,30-trien-27-ol (4i):

Following the general procedure B, the title compound (**4i**) was obtained. (Yield dichloromethane- 95% and ethyl acetate- 19%) (Product ratio: *E:Z*; 98:2); **R*f*** 0.30 (70:30, ethyl acetate:hexane); **^{1}H NMR (600 MHz, $CDCl_3$):** δ 7.72 (s, 1H), 5.93 (d, *J* = 3.7 Hz, 1H), 5.90 (d, *J* = 3.7 Hz, 1H), 5.72 (td, *J* = 15.9, 5.7 Hz, 1H), 5.68 (*J* = 15.9, 4.5 Hz, 1H), 4.86 (d, *J* = 13.7 Hz, 1H), 4.68 (d, *J* = 13.7 Hz, 2H), 4.63 (d, *J* = 3.7 Hz, 1H), 4.56 (d, *J* = 3.7 Hz, 1H), 4.53 (dd, *J* = 14.2, 7.2 Hz, 1H), 4.35 – 4.31 (m, 2H), 4.31 – 4.27 (m, 1H), 4.25 (dd, *J* = 11.8, 2.9 Hz, 1H), 4.10 (dd, *J* = 13.0, 5.0 Hz, 1H), 4.02 (d, *J* = 3.0 Hz, 1H), 3.93 (d, *J* = 3.0 Hz, 1H), 3.88 (dd, *J* = 13.0, 5.0 Hz, 1H), 3.72 – 3.67 (m, 2H), 3.50 (dd, *J* = 9.6, 5.0 Hz, 1H), 1.48 (s, 3H), 1.47 (s, 3H), 1.30 (s, 6H); **^{13}C NMR (151 MHz, $CDCl_3$):** δ 145.3, 129.7, 128.3, 123.6, 112.0, 111.8, 105.0, 104.9, 84.3, 81.9, 81.7, 80.9, 79.8, 78.0, 70.8, 70.3, 69.9, 66.3, 63.0, 53.7, 26.8, 26.7, 26.2; **HRMS (TOF, MS, ES+):** m/z calcd. for $C_{24}H_{36}N_3O_{10}$: 526.2395; found: 526.2380 ($M+H^+$).

(3*R*,4*R*,6*R*,10*R*,11*S*,14*E*,18*S*,19*R*,23*R*,25*R*)-8,8,21-Trimethyl-5,7,9,12,17,20,22,24,27-nonaoxa-1,30,31-triazahexacyclo[27.2.1.0^{4,11}.0^{6,10}.0^{18,25}.0^{19,23}]dotriaconta-14,29(32),30-trien-3-ol (4j):

Following the general procedure B, the title compound (**4j**) was obtained as yellow syrup (Yield dichloromethane- 84% and ethyl acetate- 56%) (Product ratio: *E:Z*; 95:5); **R*f*** 0.30 (70:30, Ethyl acetate:Hexane); **^{1}H NMR (600 MHz, $CDCl_3$):** δ 7.70 (s, 1H), 5.93 (d, *J* = 3.7 Hz, 1H), 5.87 (d, *J* = 3.7 Hz, 1H), 5.60 (td, *J* = 15.8, 5.3 Hz, 1H), 5.59 (td, *J* = 15.8, 5.3 Hz, 1H), 4.83 (s, *J* = 13.3 Hz, 1H), 4.74 – 4.68 (m, 2H), 4.56 (d, *J* = 3.7 Hz, 1H), 4.51 (d, *J* = 3.7 Hz, 1H), 4.39 – 4.36 (m,

1H), 4.29 (d, J = 2.5 Hz, 2H), 4.14 (dd, J = 13.2, 5.3 Hz, 1H), 4.10 (dd, J = 12.1, 4.5 Hz, 1H), 4.00 (d, J = 2.2 Hz, 1H), 3.90 – 3.86 (m, 2H), 3.64 – 3.58 (m, 3H), 3.57 (dd, J = 12.3, 5.7 Hz, 1H), 1.49 (s, 6H), 1.30 (s, 6H); **^{13}C NMR (151 MHz, $CDCl_3$)**: δ 145.5, 129.5, 127.8, 124.1, 112.0, 111.9, 105.0, 104.7, 84.2, 81.9, 81.7, 81.3, 80.6, 78.3, 70.3, 69.9, 69.0, 65.7, 65.3, 53.7, 26.7, 26.2, 26.2; **HRMS (TOF, MS, ES+):** m/z calcd. for $C_{24}H_{36}N_3O_{10}$: 526.2395; found: 526.2381 ($M+H^+$).

[(3*R*,5*R*,9*R*,10*S*,13*E*,17*R*,18*R*,20*R*,24*R*,25*S*)-7,7,22,22-Tetramethyl-4,6,8,11,16,19,21,23,26-nonaoxa-1,29,30-triazahexacyclo[26.2.1.0^{3,10}.0^{5,9}.0^{18,25}.0^{20,24}]hentriaconta-13,28(31),29-trien-17-yl]methanol (4k):

Following the general procedure B, the title compound (**4k**) was obtained (Yield dichloromethane- 81% and ethyl acetate- 96%) (Product ratio: *E:Z*; 95:5); **R_f** 0.53 (neat ethyl acetate); **^{1}H NMR (600 MHz, $CDCl_3$):** δ 7.58 (s, 1H), 5.94 (d, J = 3.7 Hz, 1H), 5.89 (d, J = 3.7 Hz, 1H), 5.60 (td, J = 15.7, 5.8 Hz, 1H), 5.55 (td, J = 15.7, 5.8 Hz, 1H), 4.89 (d, J = 12.9 Hz, 1H), 4.87 – 4.83 (m, 1H), 4.79 (d, J = 3.7 Hz, 1H), 4.66 (d, J = 13.3 Hz, 1H), 4.62 (s, 1H), 4.61 (s, 1H), 4.54 (d, J = 3.7 Hz, 1H), 4.20 (dd, J = 9.0, 3.4 Hz, 1H), 4.22 – 4.15 (m, 2H), 4.04 (d, J = 3.0 Hz, 1H), 3.94 (dd, J = 12.8, 5.4 Hz, 1H), 3.87 (dd, J = 11.9, 3.8 Hz, 1H), 3.78 – 3.72 (m, 2H), 3.65 – 3.62 (m, 2H), 1.51 (s, 3H), 1.49 (s, 3H), 1.33 (s, 3H), 1.31 (s, 3H); **^{13}C NMR (151 MHz, $CDCl_3$)**: δ 143.4, 132.6, 126.8, 124.2, 112.2, 111.9, 105.1, 104.8, 81.9, 80.2, 79.5, 79.5, 78.6, 77.6, 73.8, 69.0, 68.4, 61.9, 61.4, 47.0, 26.8, 26.7, 26.2, 26.2; **HRMS (TOF, MS, ES+):** m/z calcd. for $C_{24}H_{36}N_3O_{10}$: 526.2395; found: 526.2377 ($M+H^+$).

(3*R*,4*R*,6*R*,10*R*,11*S*,14*E*,18*S*,19*R*,23*R*,25*R*,26*R*)-26-(Methoxymethoxy)-8,8,21,21-tetramethyl-5,7,9,12,17,20,22,24,28-nonaoxa-1,31,32-triazahexacyclo [28.2.1.$0^{4,11}$.$0^{6,10}$.$0^{18,25}$.$0^{19,23}$]tritriaconta-14,30(33),31-trien-3-ol (4l):

Following the general procedure B, the title compound (**4l**) was obtained (Yield dichloromethane- 53% and ethyl acetate- 61%) (Product ratio: *E:Z*; 87:13); $\mathbf{R_f}$ 0.50 (70:30, ethyl acetate:hexane); **^{1}H NMR (600 MHz, $CDCl_3$):** δ 7.78 (s, 1H), 5.93 (d, *J* = 3.7 Hz, 1H), 5.83 (d, *J* = 3.7 Hz, 1H), 5.81 (td, *J* = 15.7, 5.2 Hz, 1H), 5.76 (td, *J* = 15.7, 5.2 Hz, 1H), 4.95 (d, *J* = 13.8 Hz, 1H), 4.79 (d, *J* = 13.8 Hz, 1H), 4.71 (d, *J* = 6.4 Hz, 1H), 4.63 (d, *J* = 6.4 Hz, 1H), 4.55 (d, *J* = 3.7 Hz, 1H), 4.52 (d, *J* = 14.0 Hz, 1H), 4.46 (d, *J* = 3.8 Hz, 1H), 4.33 (dd, *J* = 14.0, 9.35 Hz, 1H), 4.25 (t, *J* = 9.35 Hz, 1H), 4.19 (dd, *J* = 13.0, 4.6 Hz, 1H), 4.14 (d, *J* = 7.3, 2.7 Hz, 1H), 4.02 (dd, *J* = 10.3, 6.0 Hz, 1H), 3.99-3.92 (m, 3H), 3.88-3.81 (m, 3H), 3.79 (dd, *J* = 13.0, 5.0 Hz, 1H), 3.65 (t, *J* = 7.7 Hz, 1H), 3.34 (s, 3H), 1.50 (s, 3H), 1.46 (s, 3H), 1.32 (s, 3H), 1.29 (s, 3H); **^{13}C NMR (151 MHz, $CDCl_3$)**: δ 146.0, 129.6, 129.4, 123.9, 112.0, 111.8, 105.3, 104.9, 97.1, 82.4, 82.2, 81.9, 81.2, 81.0, 74.8, 74.2, 69.4, 68.9, 66.0, 55.9, 54.2, 26.8, 26.7, 26.2, 26.2; **HRMS (TOF, MS, ES+):** m/z calcd. for $C_{27}H_{42}N_3O_{12}$: 600.2763; found: 600.2780 ($M+H^+$).

(4*S*,5*R*,9*R*,11*R*,12*R*,15*E*,19*S*,20*R*,24*R*,26*R*,27*R*)-12-(Hydroxymethyl)-7,7,22,22-tetramethyl-3,6,8,10,13,18,21,23,25-nonaoxa-29,30,31-triazahexacyclo [27.2.1.$0^{4,11}$.$0^{5,9}$.$0^{19,26}$.$0^{20,24}$]-dotriaconta-1(32),15,30-trien-27-ol (4m):

Following the general procedure B, the title compound (**4m**) was obtained (Yield dichloromethane- 40% and ethyl acetate- 55%) (Product ratio: *E:Z*; 88:12); $\mathbf{R_f}$ 0.45 (75:25, ethyl acetate:hexane); **^{1}H NMR (600 MHz, $CDCl_3$):** δ 7.70 (s, 1H), 5.91 (s, 1H), 5.88 (s, 1H), 5.53 (s, 2H), 4.90 (dd, *J* = 12.7, 2.4 Hz, 1H), 4.72 (d, *J* = 12.7 Hz, 1H), 4.67 (s, 1H), 4.62 (s, 2H), 4.49

(dd, *J* = 3.5, 1.6 Hz, 1H), 4.45 (bs, 1H), 4.30 (d, *J* = 1.6 Hz, 1H), 4.20 (m, 1H), 4.13 (d, *J* = 13.2 Hz, 1H), 4.07 (m, 3H), 3.94 (dd, *J* = 12.9, 4.5 Hz, 1H), 3.91 (s, 1H), 3.88 (d, *J* = 12.9 Hz, 1H), 3.74 (d, *J* = 3.7 Hz, 2H), 3.27 (dd, *J* = 12.9, 5.2 Hz, 1H), 1.47 (s, 3H), 1.31 (s, 3H), 1.28 (s, 3H), 1.23 (s, 3H); **^{13}C NMR (151 MHz, $CDCl_3$)**: δ 145.1, 130.1, 128.0, 124.0, 112.0, 111.9, 104.9, 104.2, 84.6, 81.5, 80.8, 80.3, 78.9, 72.9, 70.2, 69.7, 67.7, 63.3, 60.6, 53.5, 26.7, 26.2, 26.1; **HRMS (TOF, MS, ES+):** m/z calcd. for $C_{25}H_{38}N_3O_{11}$: 556.2501; found: 556.2527 ($M+H^+$).

[(4*S*,5*R*,9*R*,11*R*,12*R*,15*E*,19*S*,20*R*,24*R*,26*R*,27*R*)-27-(Acetyloxy)-7,7,22,22-tetramethyl-3,6,8,10,13,18,21,23,25-nonaoxa-29,30,31-triazahexacyclo[27.2.1.0^{4,11}.0^{5,9}.0^{19,26}.0^{20,24}] dotriaconta-1(32),15,30-trien-12-yl]methyl acetate (5):

The macrocycle **4m** (0.120 g, 0.21 mmol) dissolved in pyridine (5 mL) under argon cooled at 0 °C was added acetyl chloride (0.041 g, 0.518 mmol) and DMAP (0.003 g, 0.024 mmol). The reaction mixture was stirred at room temperature for 16 hours. After completion (TLC), the pyridine was removed under reduced pressure and the residue was dissolved in ethyl acetate (50 mL), washed sequentially with 1 N HCl (20 mL), water (50 mL), brine (20 mL) and the organic layers were dried over Na_2SO_4. The solvent was removed under reduced pressure and the residue obtained was purified by silica gel column chromatography to afford diacetate **5** as syrup (0.108 g, 81%) (Product ratio: *E:Z*; 88:12); **R_f** 0.45 (80:20, ethyl acetate:hexane); **^{1}H NMR (600 MHz, $CDCl_3$):** δ 7.62 (s, 1H), 5.97 (d, *J* = 3.7 Hz, 1H), 5.88 (d, *J* = 3.7 Hz, 1H), 5.79 (td, *J* = 15.7, 5.0 Hz, 1H), 5.68 (dd, *J* = 15.7, 5.0 Hz, 1H), 5.44 (d, *J* = 9.0 Hz, 1H), 4.89 (d, *J* = 13.8 Hz, 1H), 4.81 (d, *J* = 15.0 Hz, 1H), 4.72 (m, 3H), 4.66 (dd, *J* = 14.9, 9.5 Hz, 1H), 4.59 (d, *J* = 3.7 Hz, 1H), 4.40 (d, *J* = 3.7 Hz, 1H), 4.28 (dd, *J* = 12.3, 5.1 Hz, 1H), 4.26-4.20 (m, 2H), 4.07-4.02 (m, 3H), 3.91 (dd, *J* = 12.4, 4.7 Hz, 1H), 3.89-3.84 (m, 2H), 2.08 (s, 3H), 2.06 (s, 3H), 1.47 (s, 3H), 1.45 (s, 3H), 1.32 (s, 3H), 1.31 (s, 3H); **^{13}C NMR (151 MHz, $CDCl_3$)**: δ 170.9, 170.5, 144.7, 129.6,

127.4, 122.2, 112.2, 111.8, 105.4, 104.7, 84.8, 81.4, 81.2, 80.8, 79.1, 78.2, 73.4, 71.9, 70.2, 68.4, 62.3, 62.1, 51.3, 26.8, 26.3, 26.2, 21.0, 20.9; **HRMS (TOF, MS, ES+):** m/z calcd. for $C_{29}H_{42}N_3O_{13}$: 640.2712; found: 640.2702 ($M+H^+$).

References

[SR1] Jayachandra, R.; Reddy, S.R. *RSC Adv.* **2016**, 6(46), 39758 – 39761.

[SR2] Rapi, Z.; Ozohanics, O.; Toth, G.; Bako, P.; Hofler, L.; Nemcsok, T.; Kanya, N.; Keglevich, G. *J. incl. Phenom. Macrocycle Chem.* **2006**, 85, 19-32.

[SR3] Bauer, T.; Majdechi, M.; Jurczak, J. *Tetrahedron* **2013**, 69, 1930-1939.

[SR4] Wuts, P.G.M.; Greene, T.W. *Greene's Protective Groups in Organic Synthesis, Fourth Edition* Wiley: India **2007**, P.P. 197.

[SR5] Rochet, P.; Vatele, JM.; Gore, J. *Synthesis* **1994**, 795 – 799.

[SR6] Raluy, E.; Pamies, O.; Dieguez, M. *Adv. Synth. Catal.* **2009**, 351, 1648-1680.

[SR7] Azad, CS.; Khan, I.A.; Narula, A.K. *Org. Biomol. Chem.* **2016**, 14, 11454 – 11461.

[SR8] Totani, K.; Shinoda, Y. Shiba, M.; Iwamoto, S.; Koizumi, A,; Matsuzaki, Y.; Hirano, M. *RSC Adv*. **2015**, 5, 75918 – 75922.

[SR9] Torrente, S.; Noya, B.; Branchadell, V.; Alonso, R. *J. Org. Chem.* **2003**, 68, 4772 – 4783.

[SR10] Wuts, P.G.M.; Greene, T.W. *Greene's Protective Groups in Organic Synthesis, Fourth Edition* Wiley: India **2007**, P.P. 30.

[SR11] Mishra, A.; Mishra, B.B.; Tiwari, V.K. *RSC Adv.* **2015**, 5, 41520 – 41535.

[SR12] Gruner, S. A. W.; Truffault, V.; Voll, G.; Locardi, E.; Stockle, M.; Kessler, H. *Chem. Eur. J.* **2002**, 8, 4365-4376;

[SR13] Cipolla, L.; Airoldi, C.; Sperandeo, P.; Gianera, S.; Polissi, A.: Nicotra, F.; Gabrielli, L. *Carbohydrate Research* **2014**, 389, 186 – 191; Gabrielli, L.; Airoldi, C.; Sperandeo, P.; Gianera, S.; Polissi, A.: Nicotra, F.; Cipolla, L. *Eur. J. Org. Chem.* **2013**, 7776 – 7784; (a) Dahlman, O.; Garegg, P. J.; Mayer, H.; Schramek, S. *Acta Chem. Scand. B* **1986**, 40, 15-20; (b) Doboszewski, B.; Herdewijn, P. *Tetrahedron* **1996**, 52, 1651-1668; (c) Doboszewski, B.; Herdewijn, P. *Tetrahedron* **2008**, 64, 5551–5562; (d) Soares, F. D. P.; Silva, M. J.; Doboszewski, B. *Carbohydrate Research* **2013**, 380, 143-148.

[SR14] Izumi, M.; Tsuruta, O.; Hashimoto, H. *Carbohydrate Research* **1996**, 280, 287 – 302.

[SR15] Hashimoto, H.; Izumi, M. *Chemistry Letter* **1992,** 25 - 28.

NMR spectrum of building blocks (S55-S68)

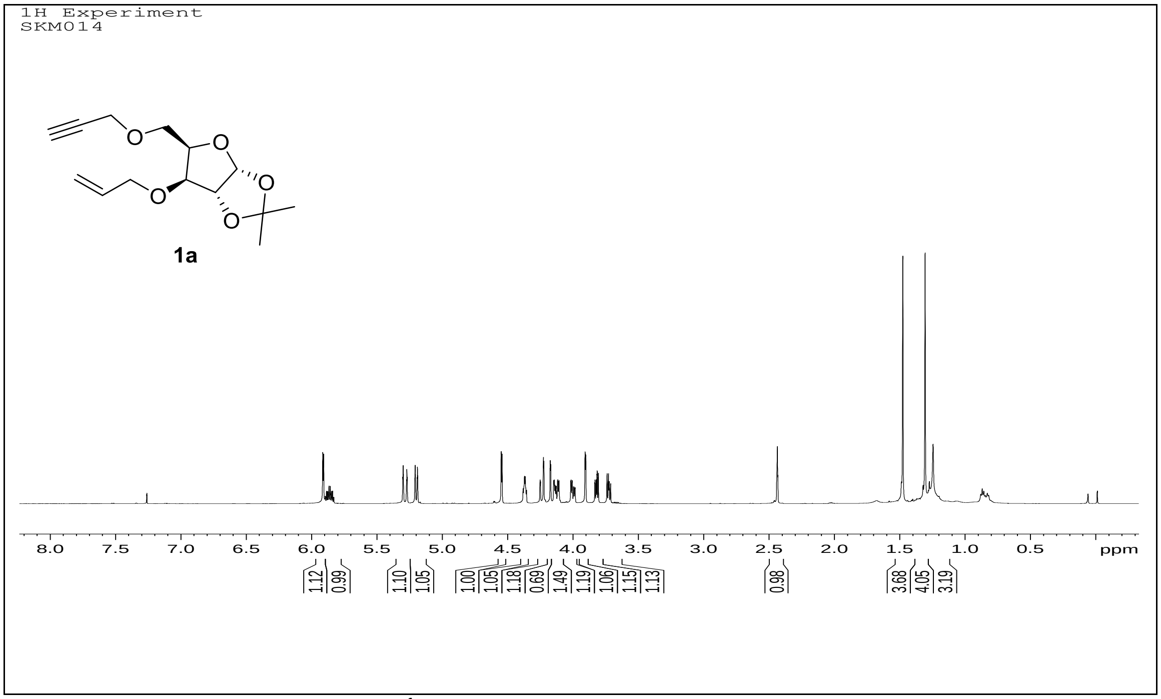

^{1}H NMR spectrum of compound 1a in $CDCl_3$

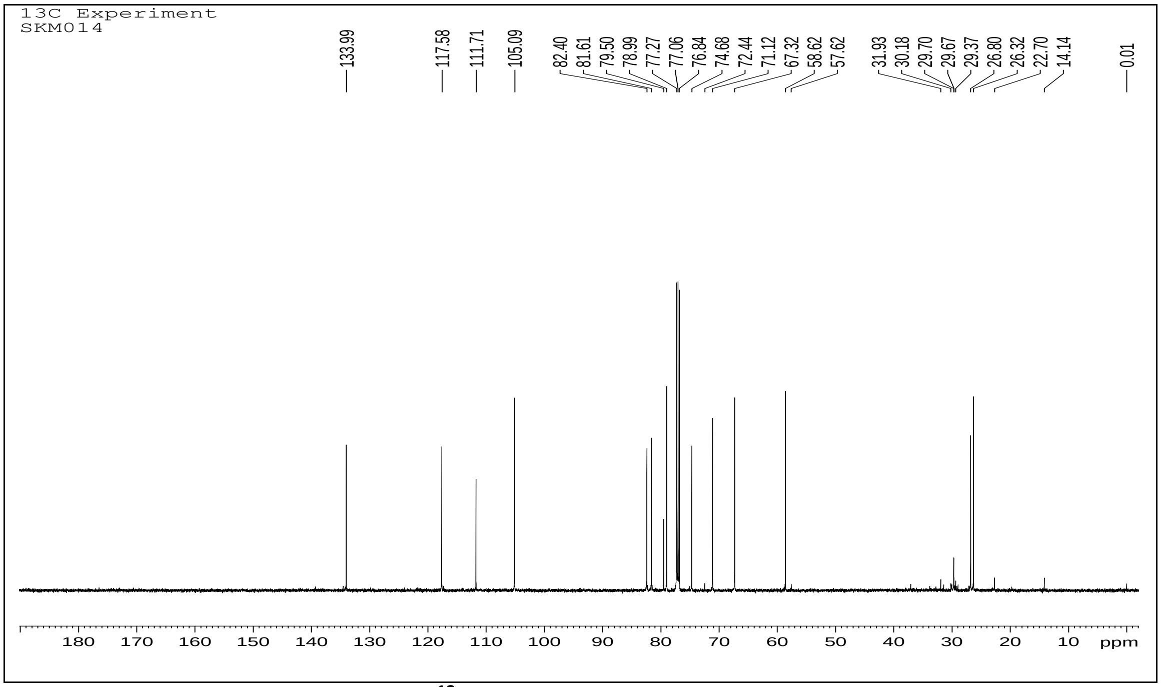

^{13}C spectrum of compound 1a in $CDCl_3$

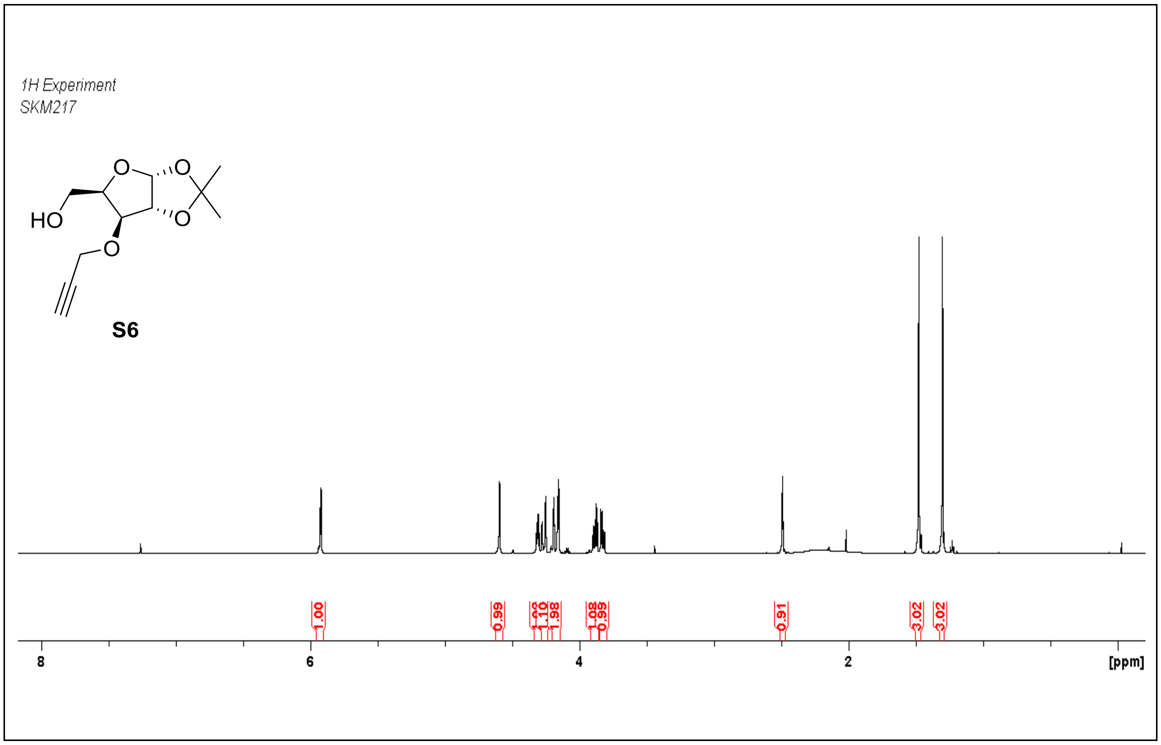

^{1}H NMR spectrum of compound S6 in $CDCl_3$

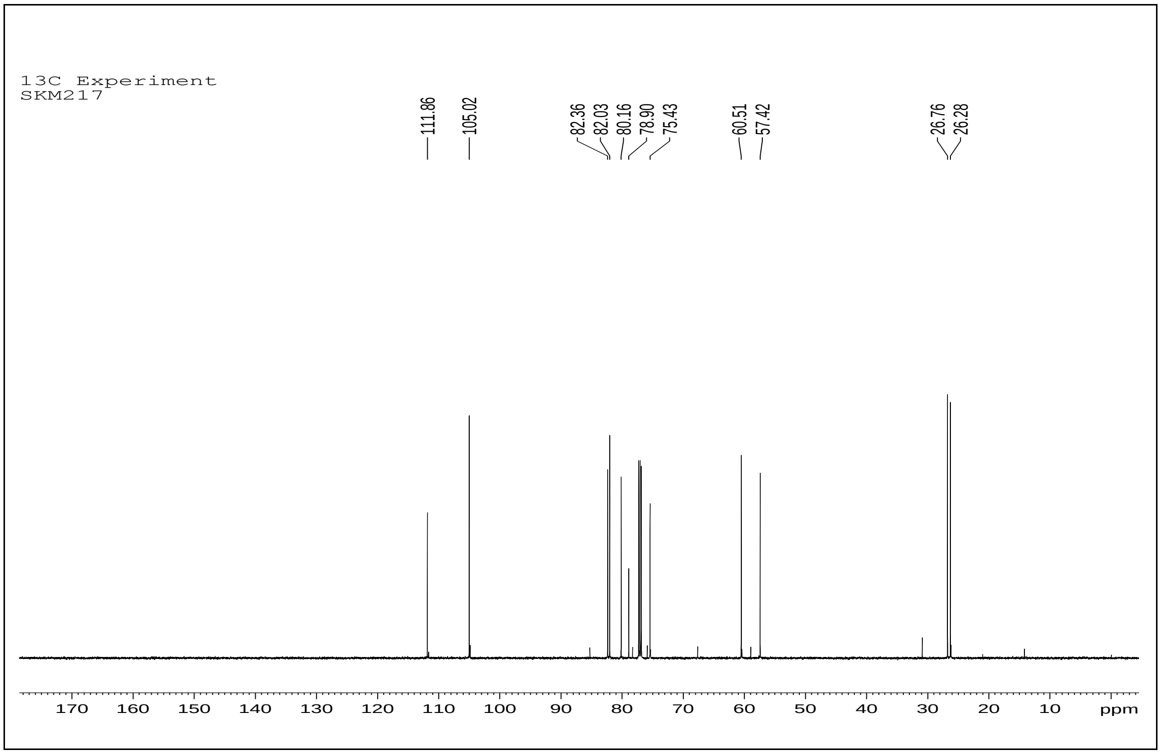

^{13}C spectrum of compound S6in $CDCl_3$

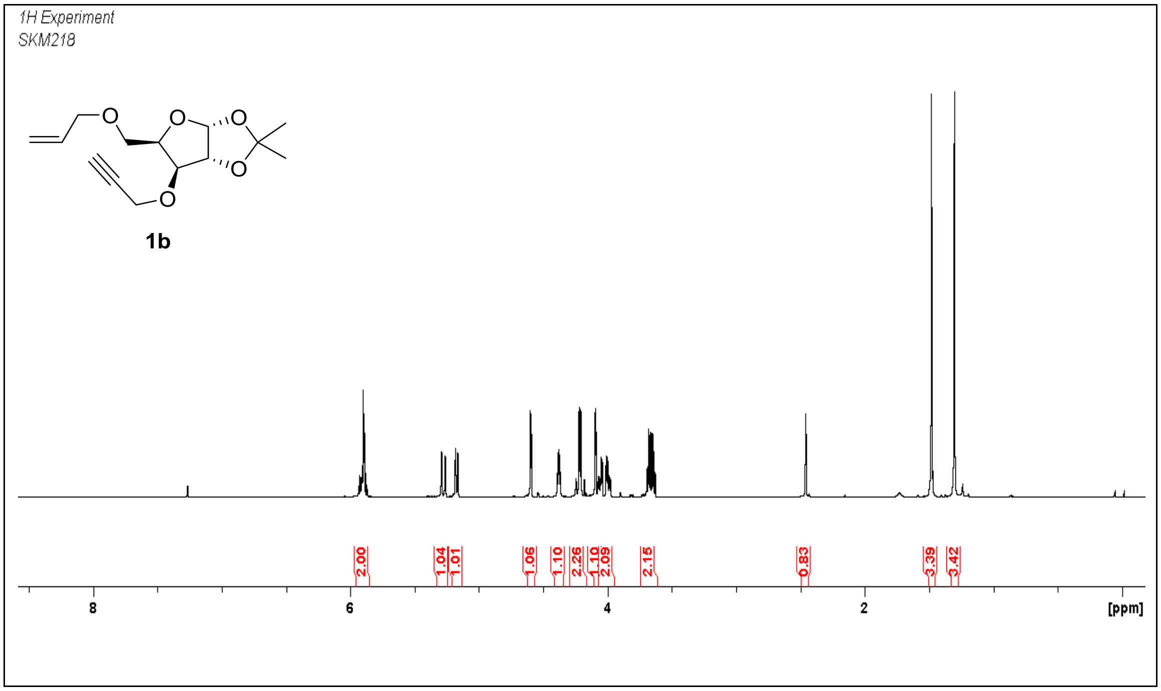

^{1}H NMR spectrum of compound 1b in $CDCl_3$

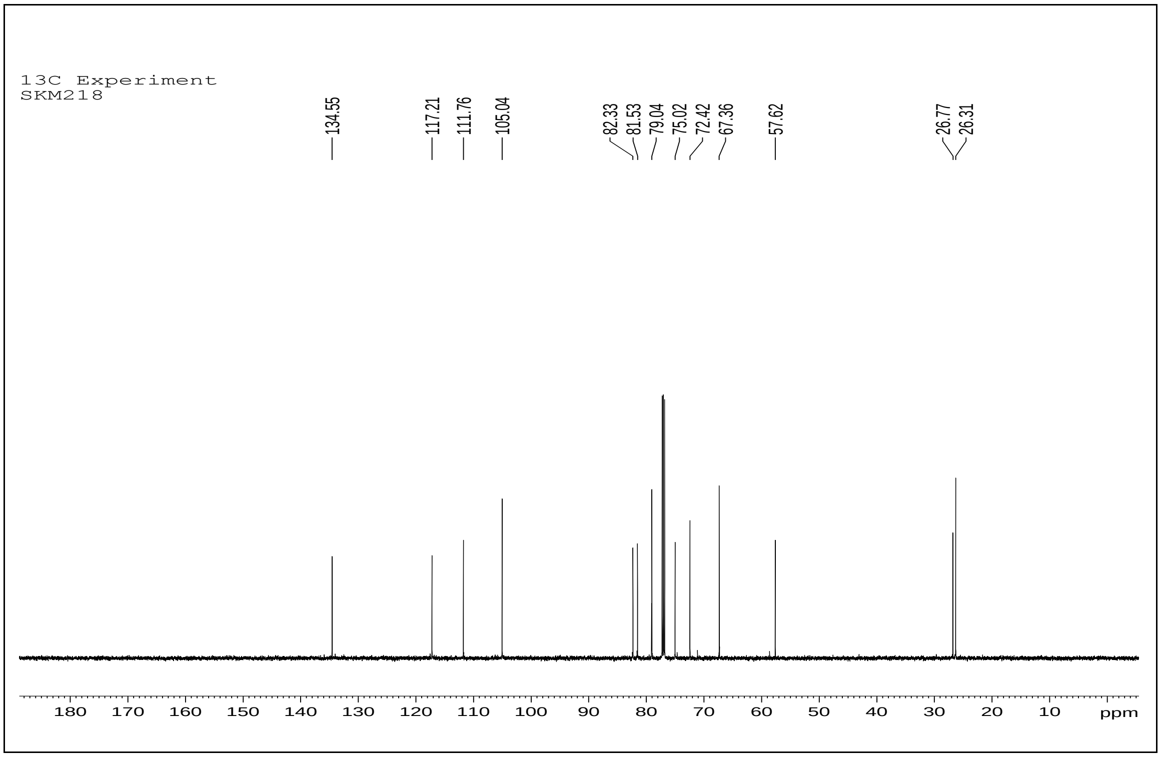

^{13}C spectrum of compound 1b in $CDCl_3$

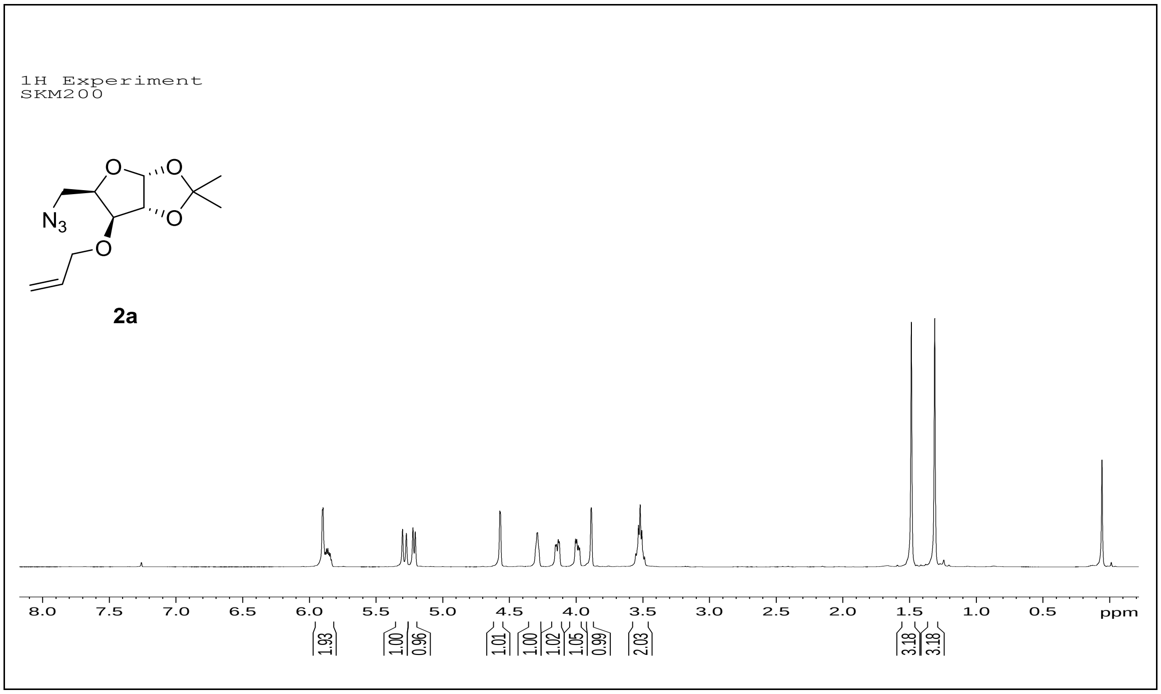

^{1}H NMR spectrum of compound 2a in $CDCl_3$

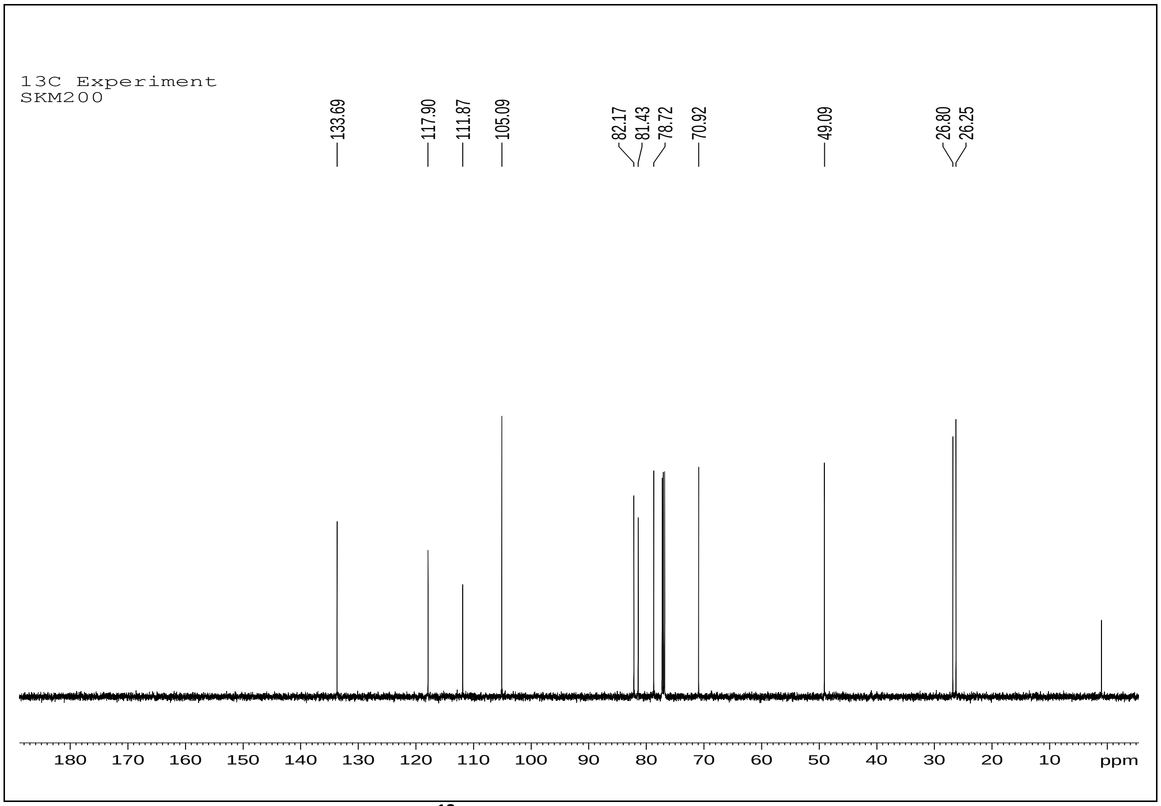

^{13}C spectrum of compound 2a in $CDCl_3$

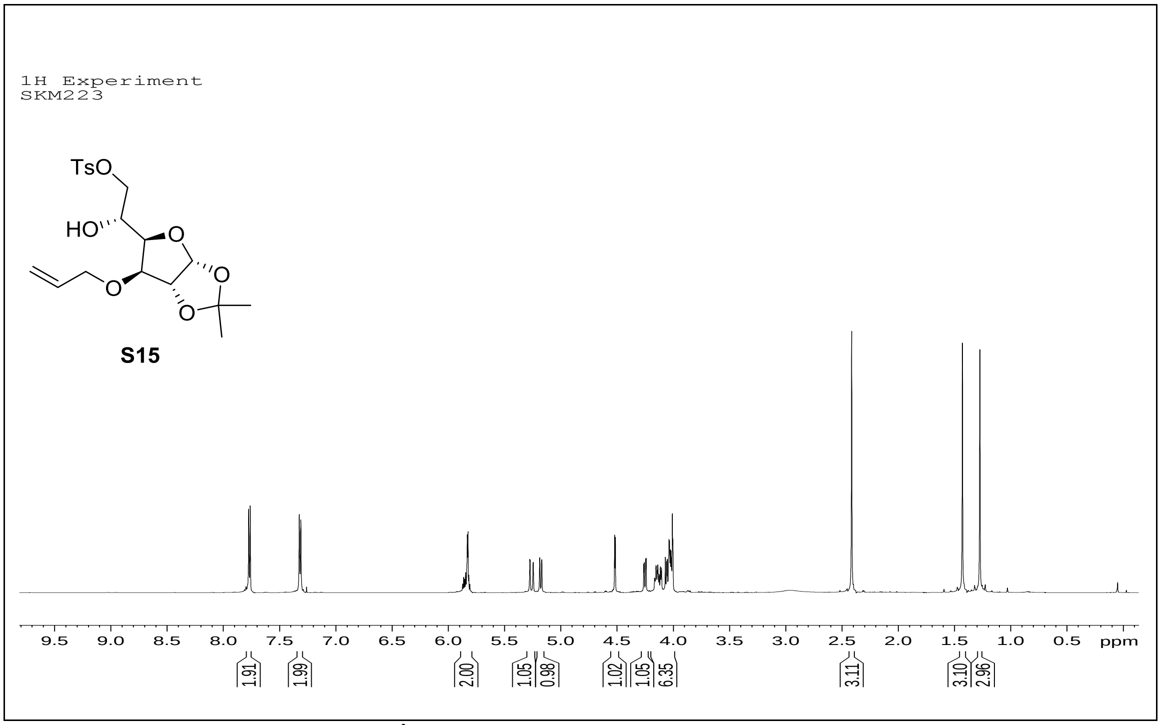

^{1}H NMR spectrum of compound S15 in $CDCl_3$

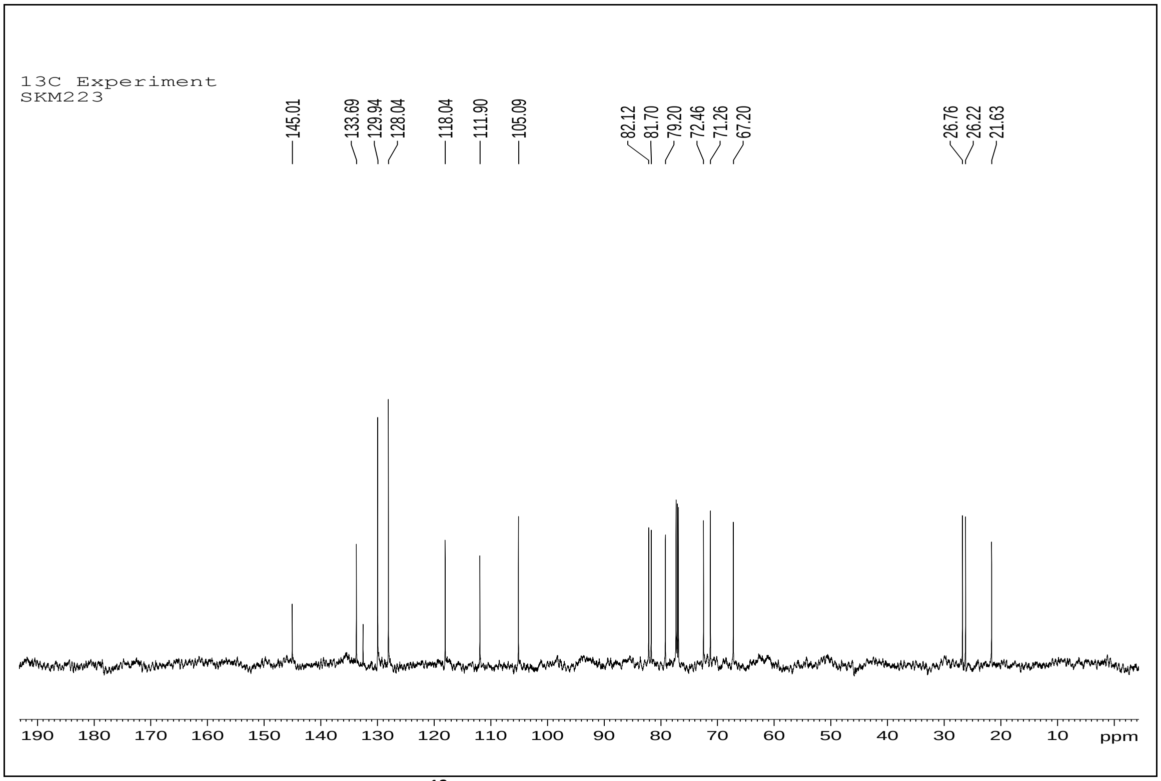

^{13}C spectrum of compound S15 in $CDCl_3$

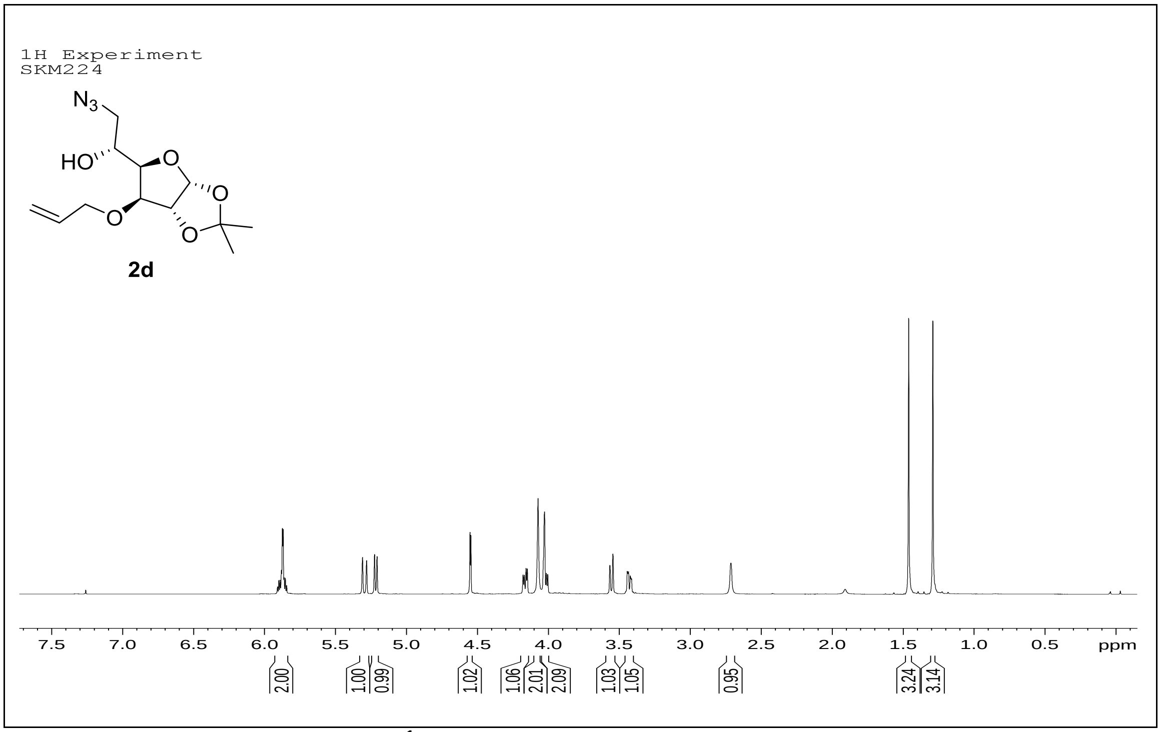

^{1}H NMR spectrum of compound 2d in $CDCl_3$

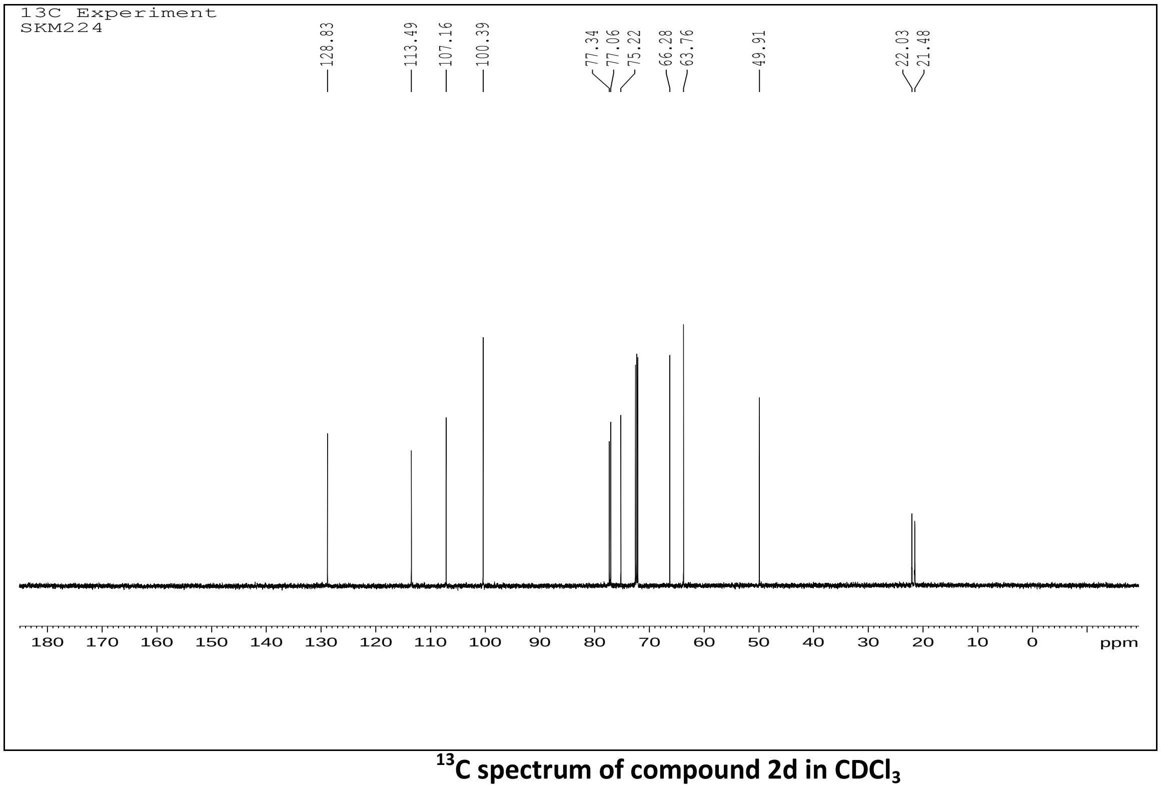

^{13}C spectrum of compound 2d in $CDCl_3$

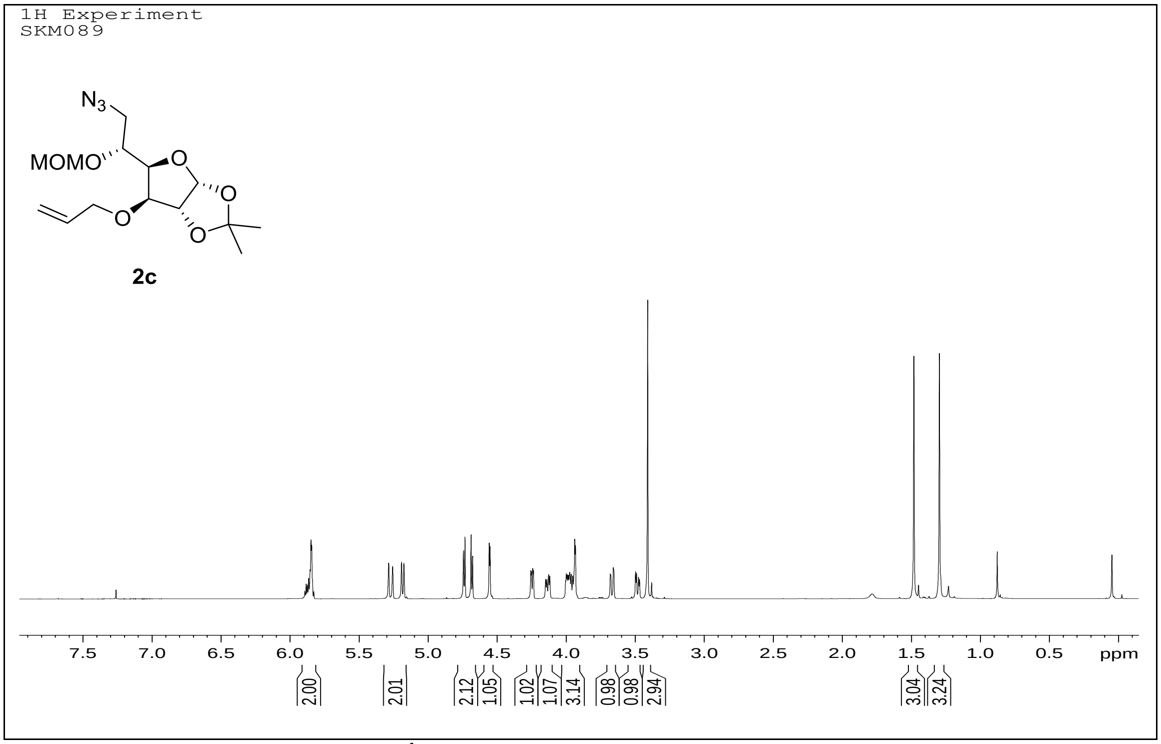

^{1}H NMR spectrum of compound 2c in $CDCl_3$

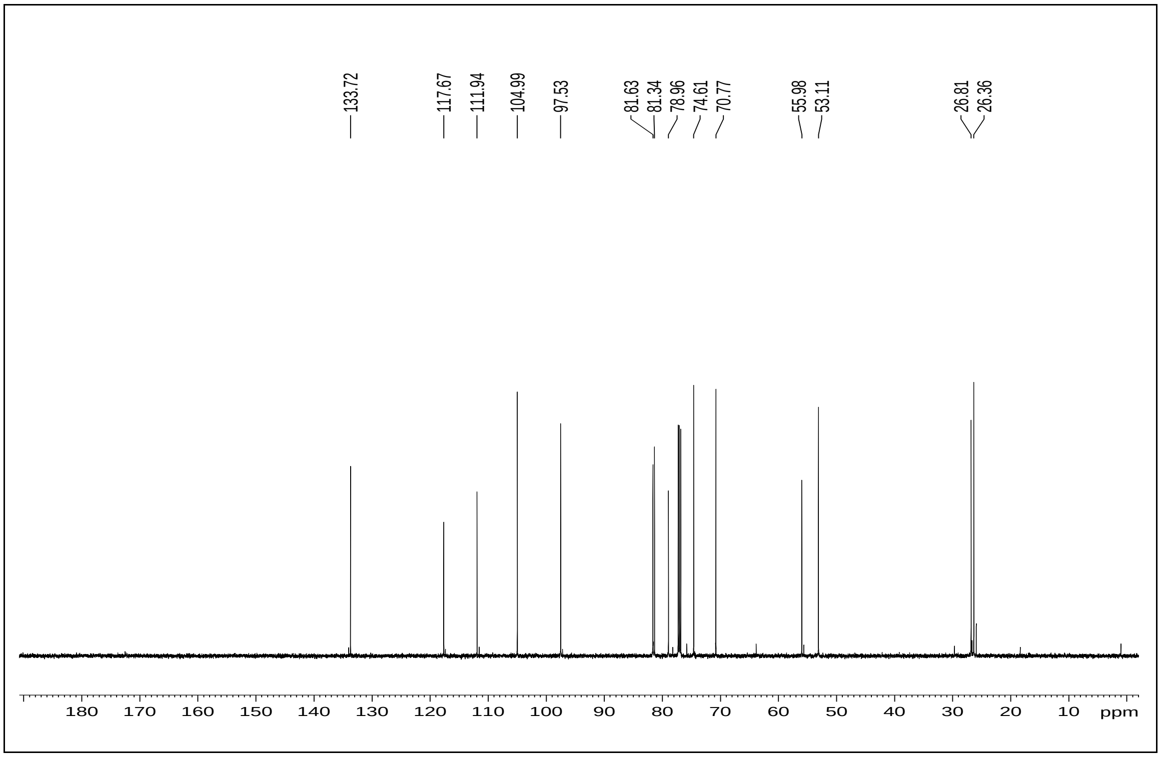

^{13}C spectrum of compound 2c in $CDCl_3$

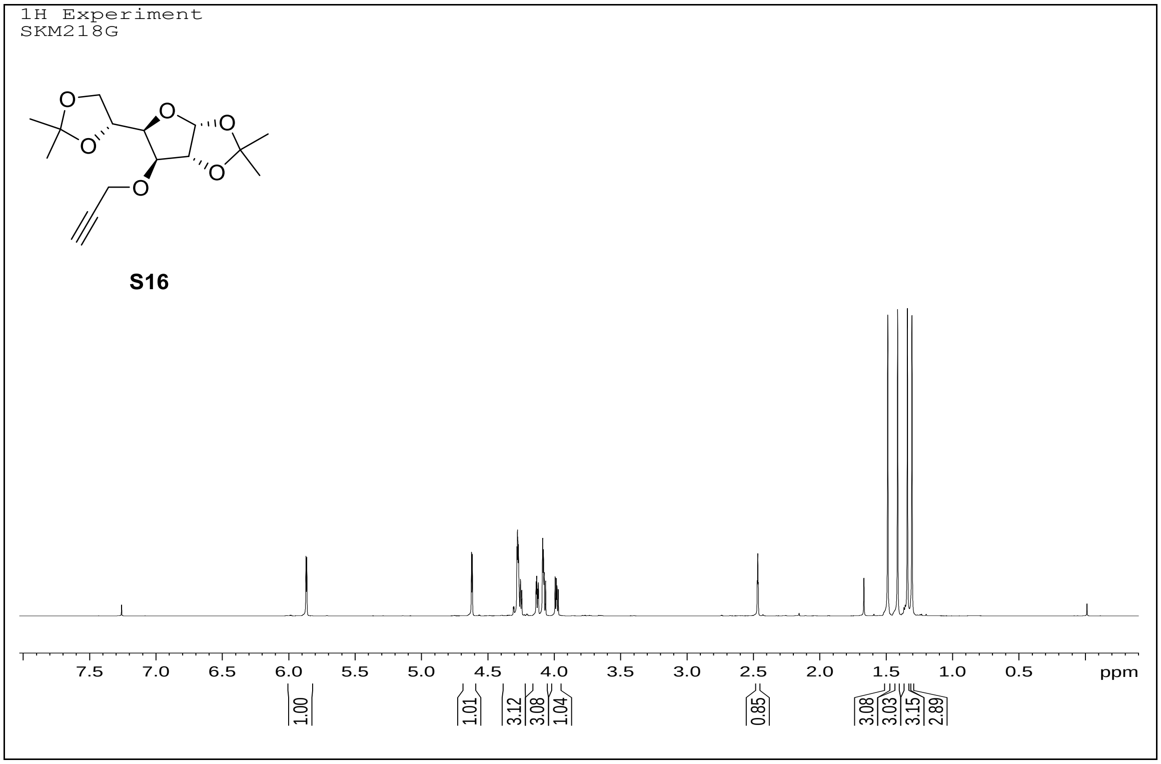

^{1}H NMR spectrum of compound S16 in $CDCl_3$

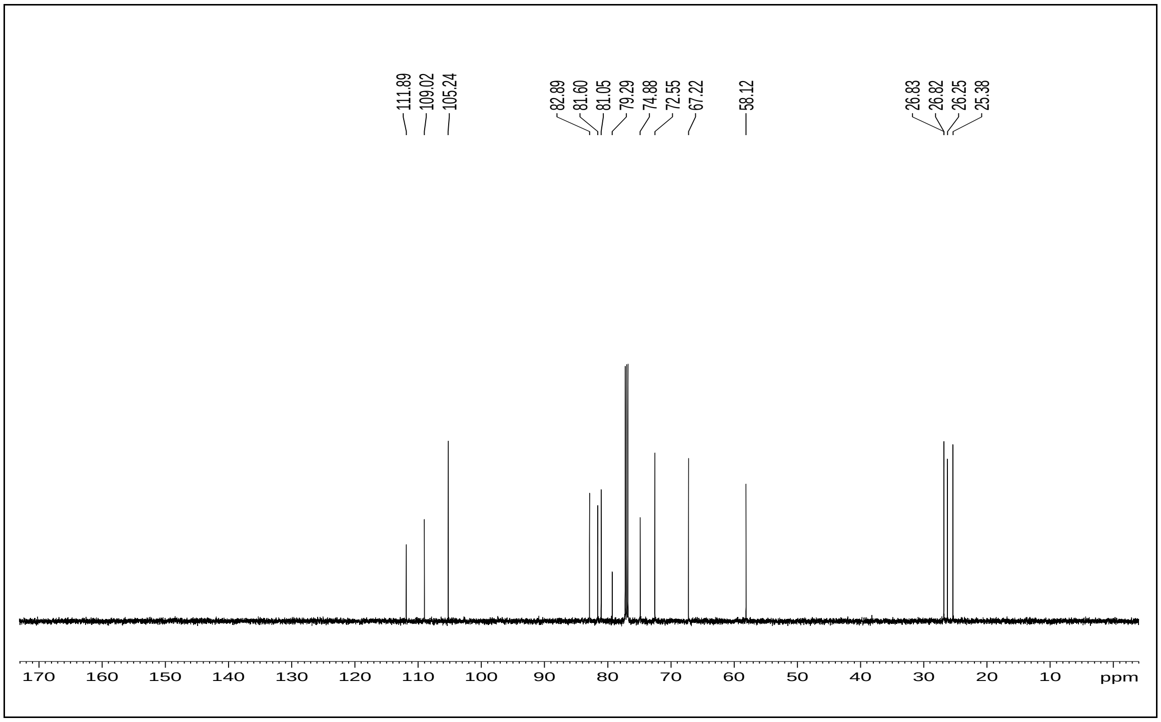

^{13}C spectrum of compound S16 in $CDCl_3$

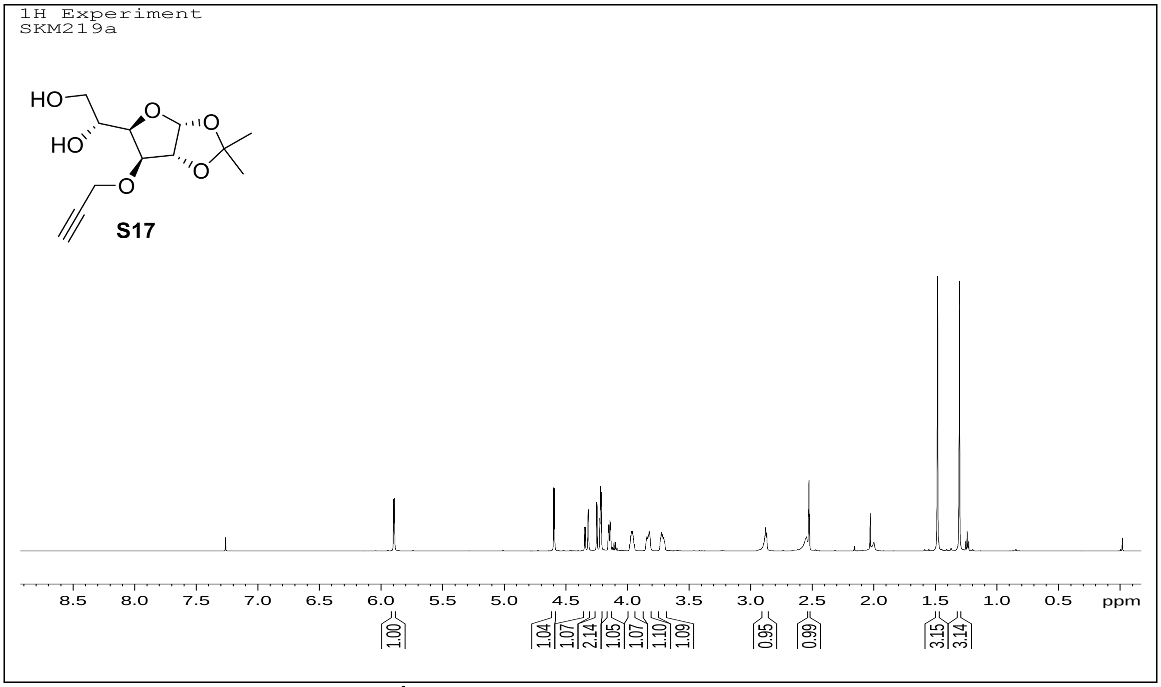

^{1}H NMR spectrum of compound S17 in $CDCl_3$

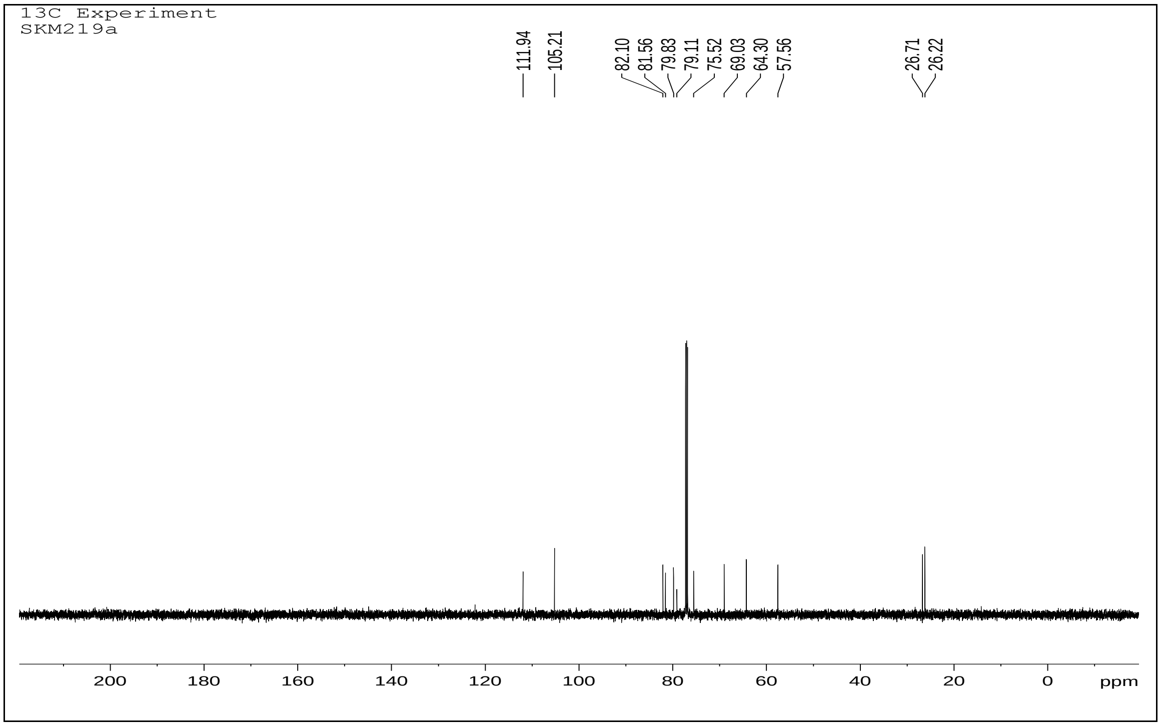

^{13}C spectrum of compound S17 in $CDCl_3$

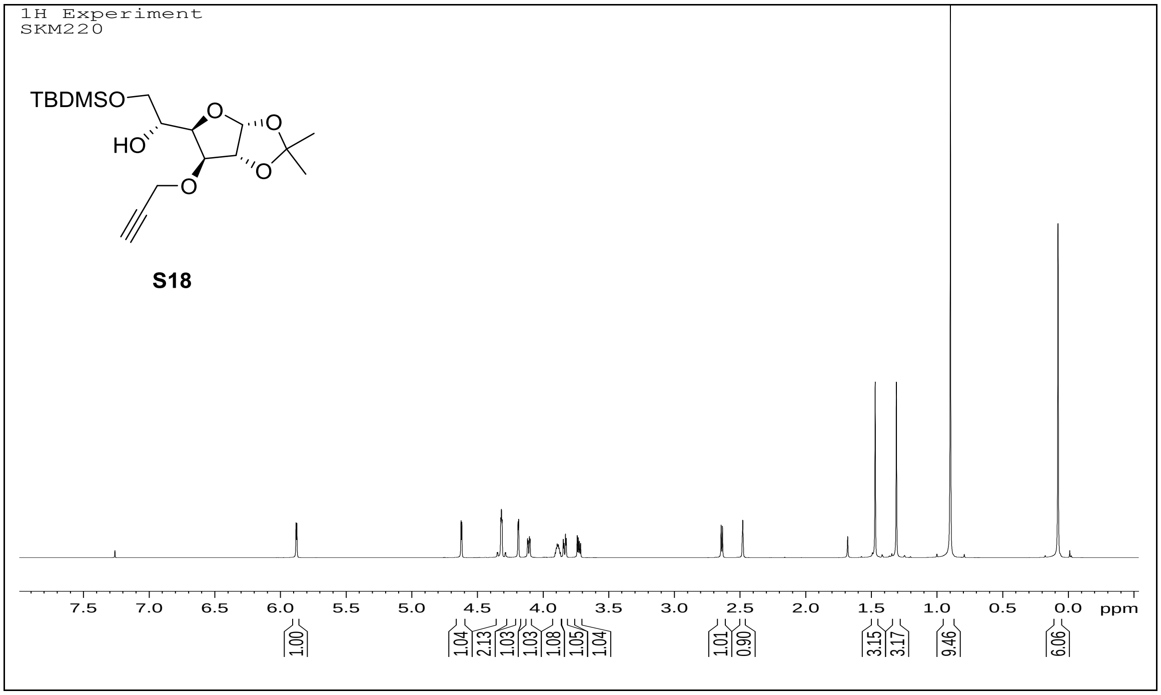

^{1}H NMR spectrum of compound S18 in $CDCl_3$

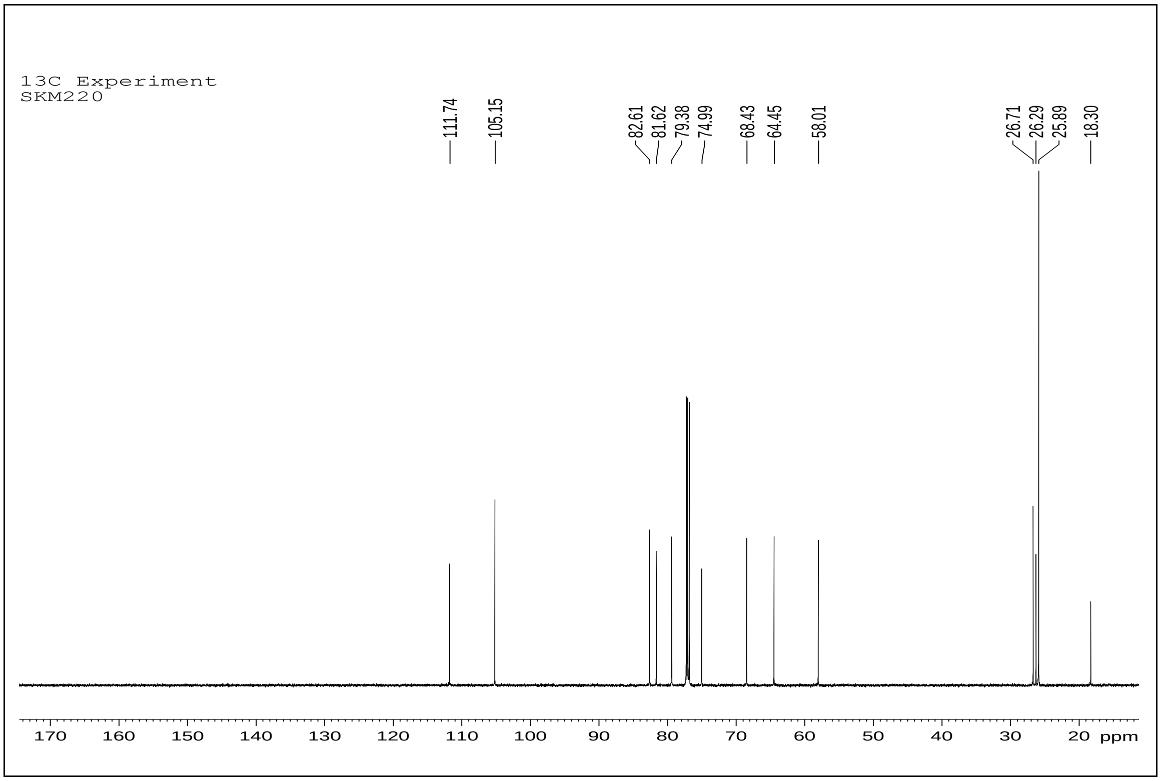

^{13}C spectrum of compound S18 in $CDCl_3$

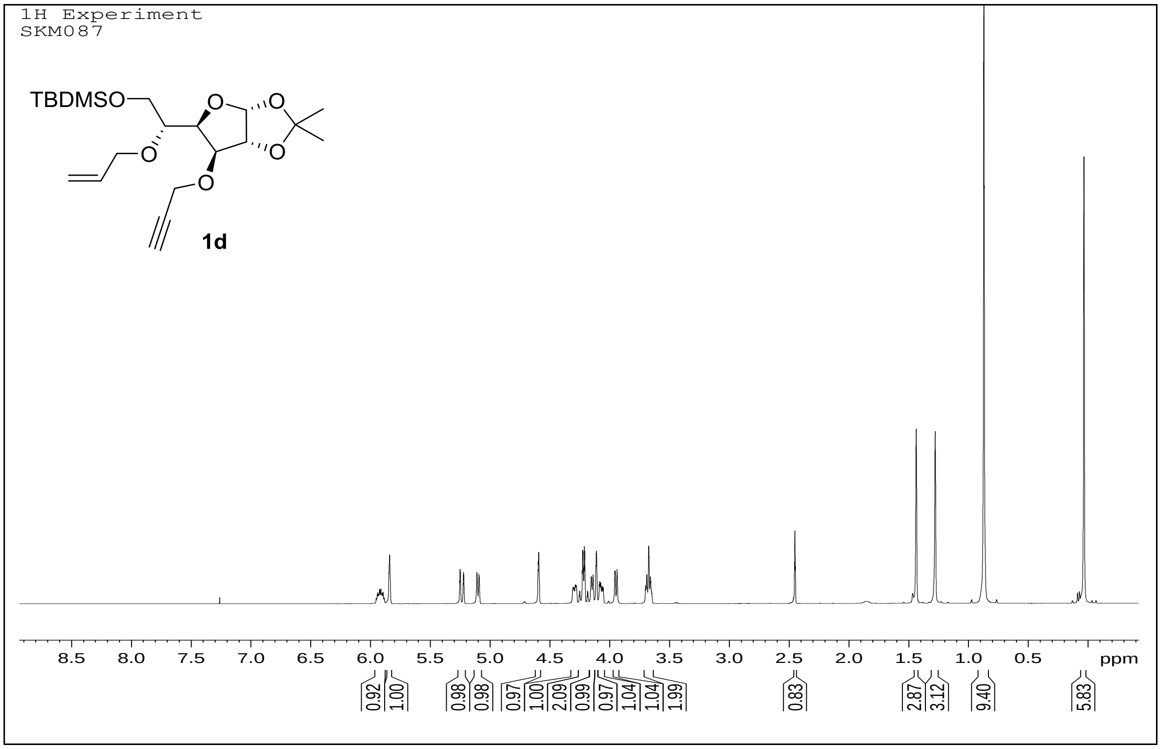

^{1}H NMR spectrum of compound 1d in $CDCl_3$

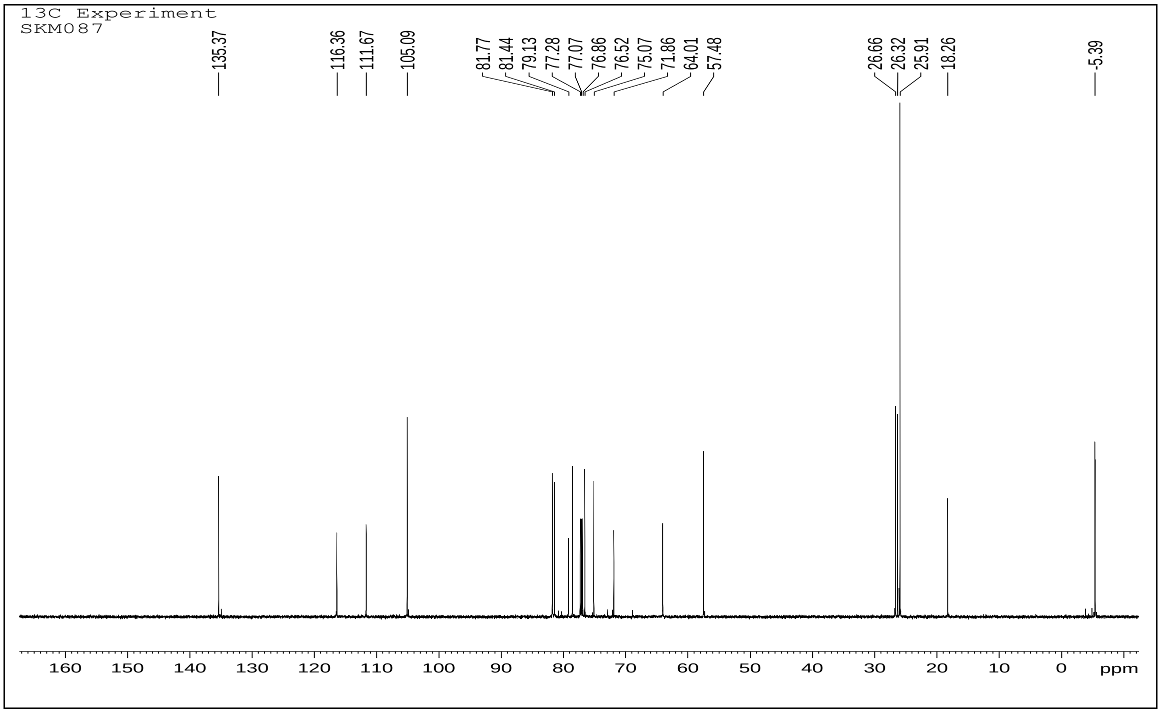

^{13}C spectrum of compound 1d in $CDCl_3$

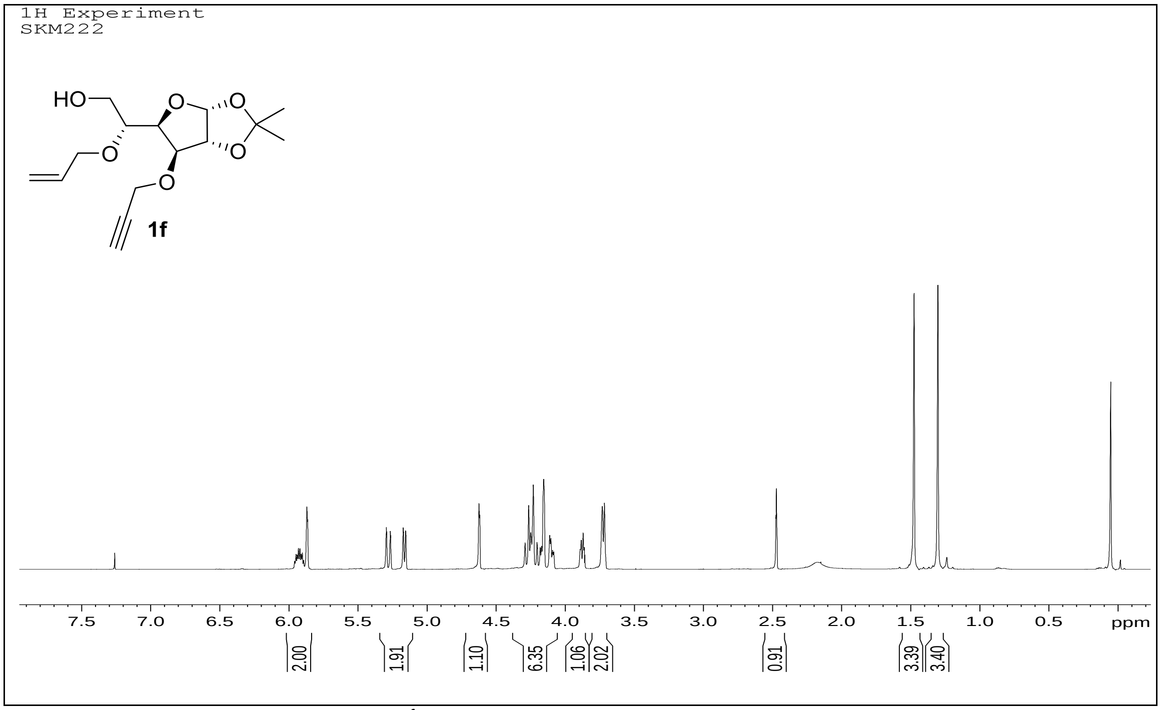

^{1}H NMR spectrum of compound 1f in $CDCl_3$

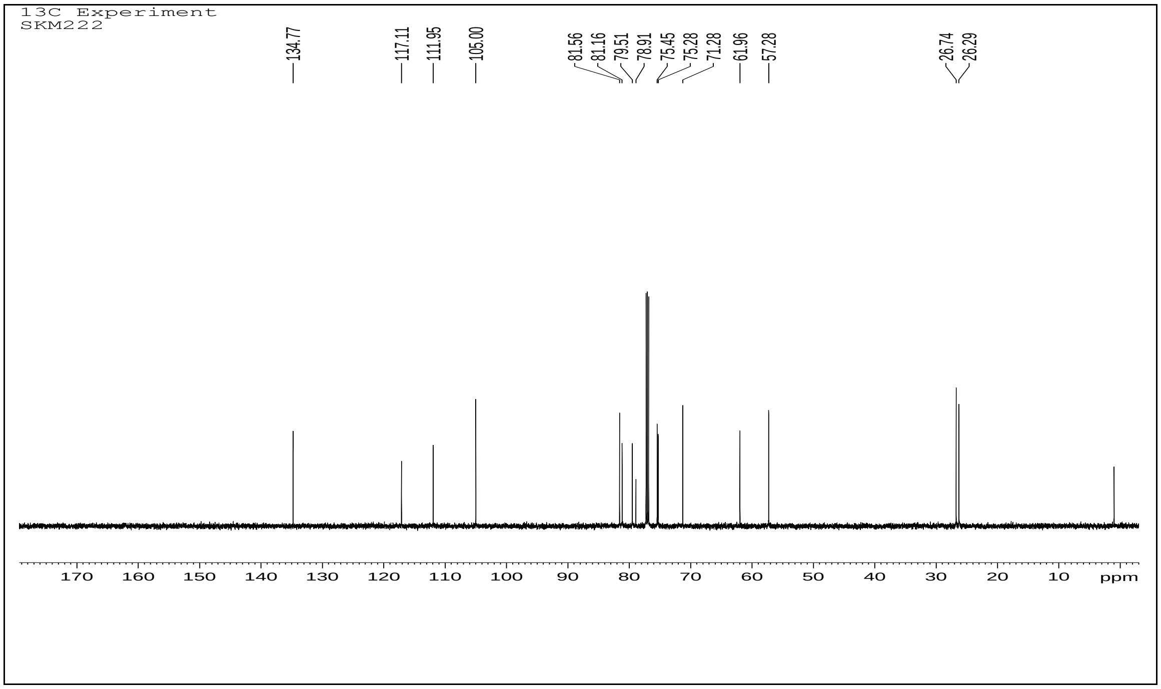

^{13}C spectrum of compound 1f in $CDCl_3$

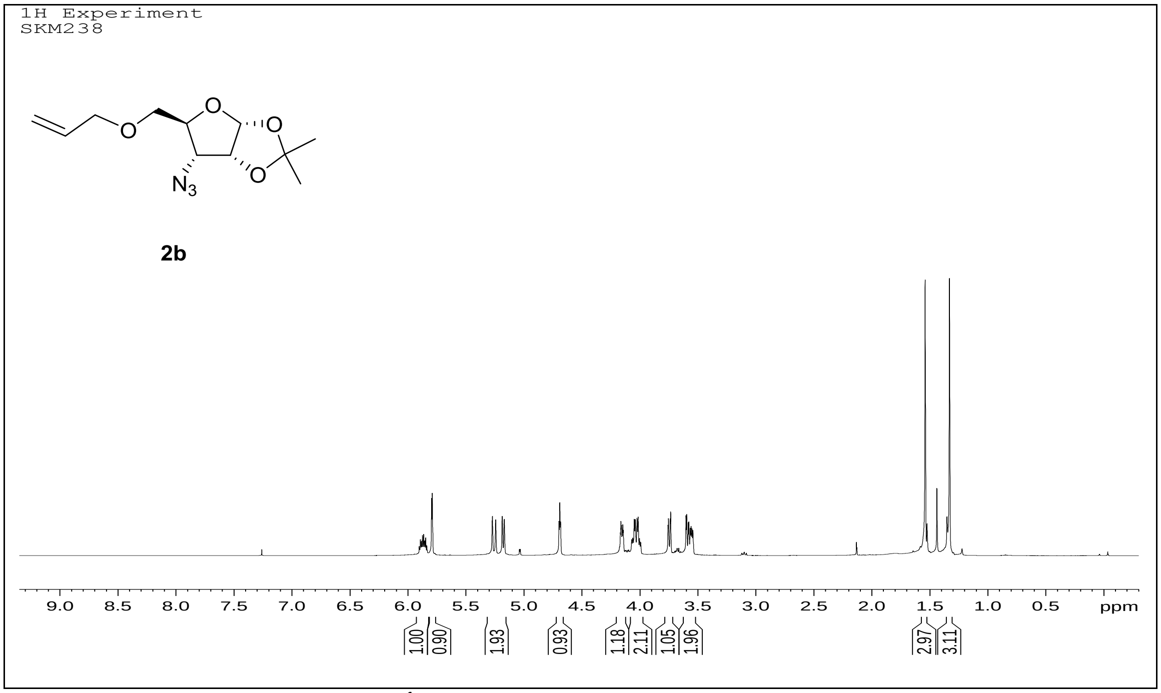

^{1}H NMR spectrum of compound 2b in $CDCl_3$

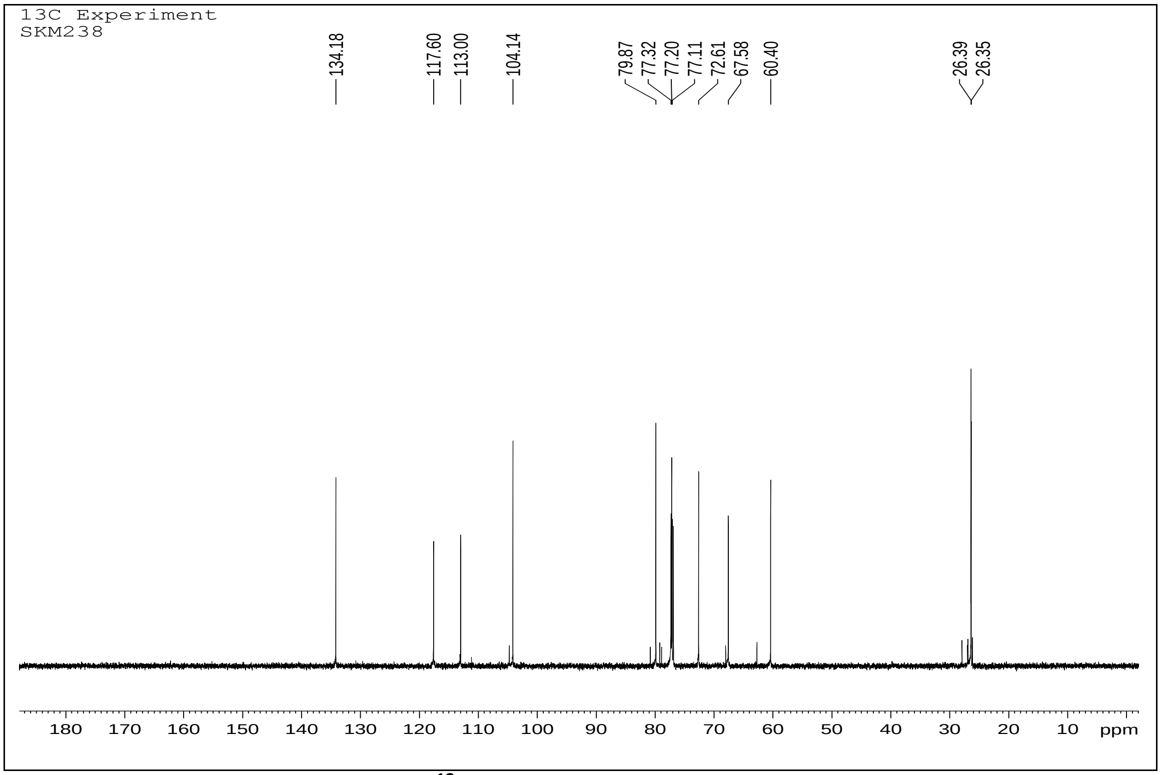

^{13}C spectrum of compound 2b in $CDCl_3$

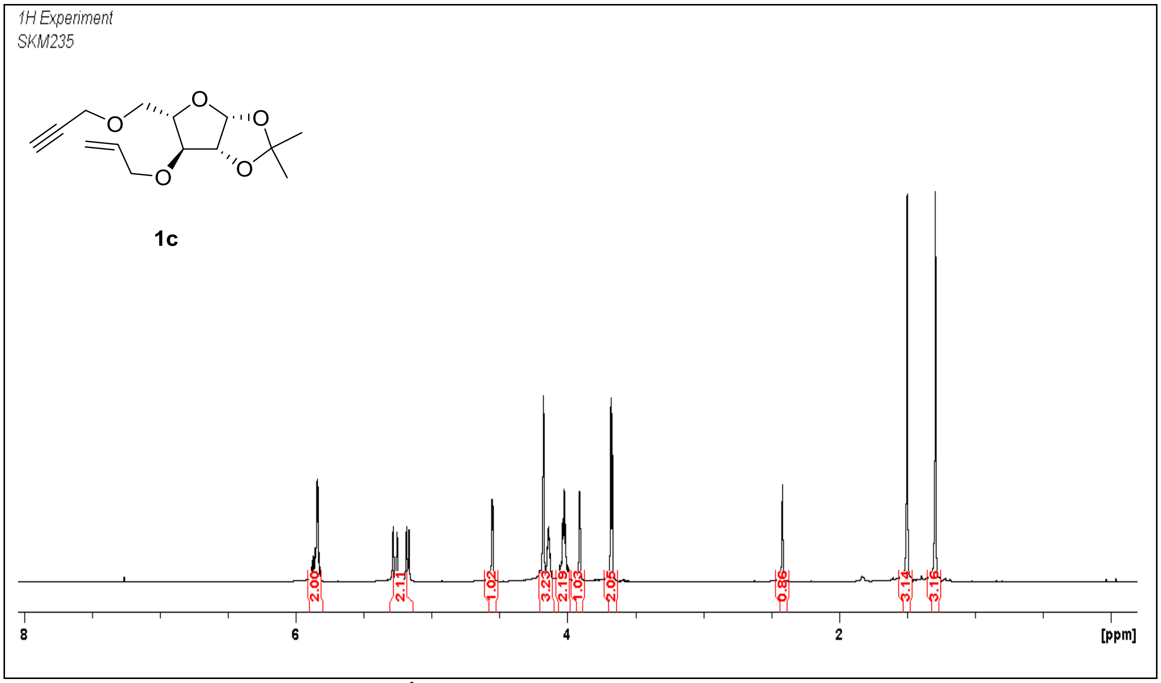

^{1}H NMR spectrum of compound 1c in $CDCl_3$

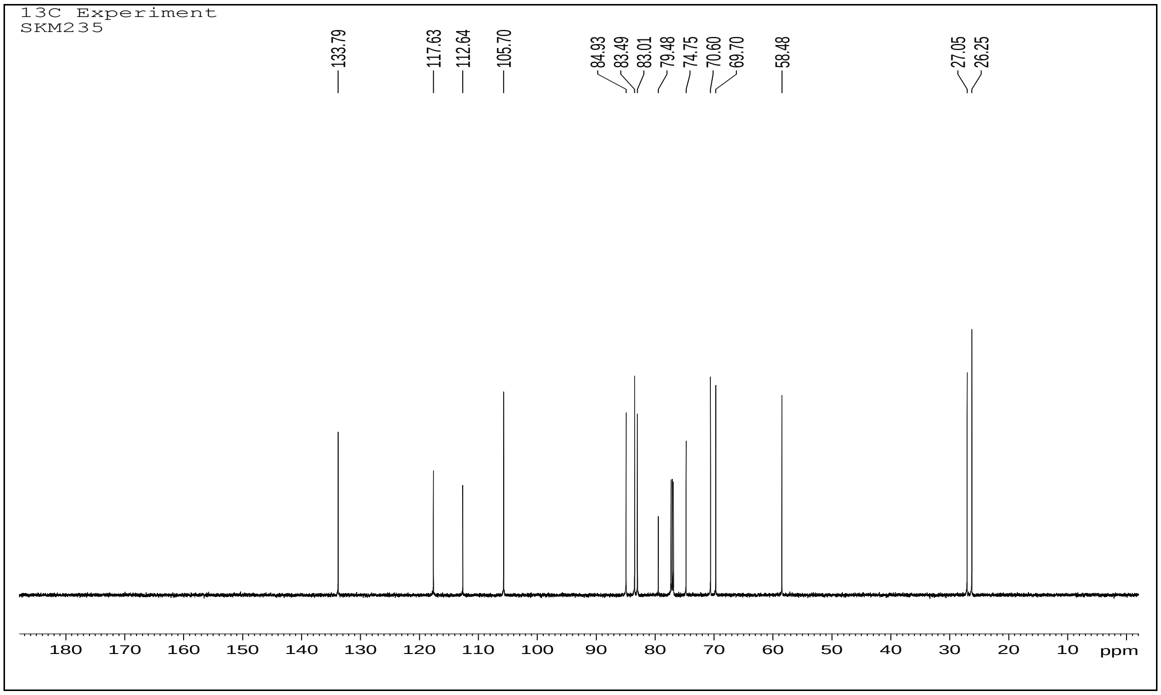

^{13}C spectrum of compound 1c in $CDCl_3$

NMR spectrum of Click Products (S69-S81)

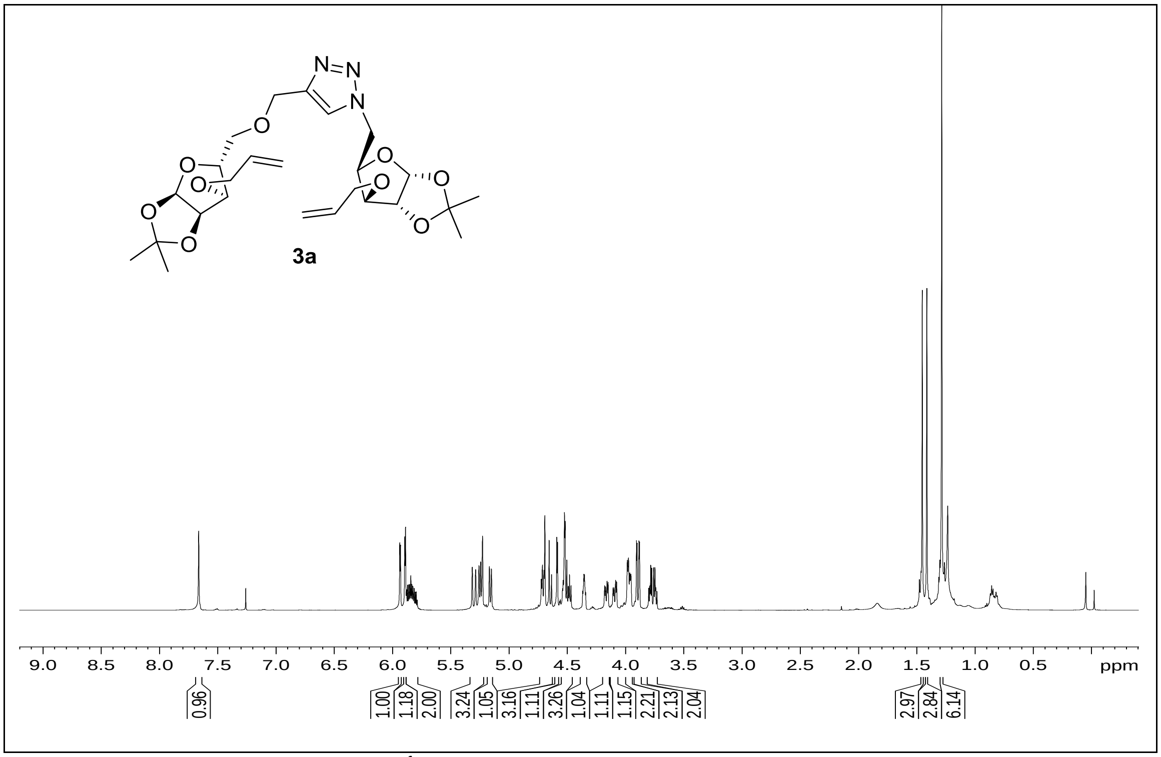

^{1}H NMR spectrum of compound 3a in $CDCl_3$

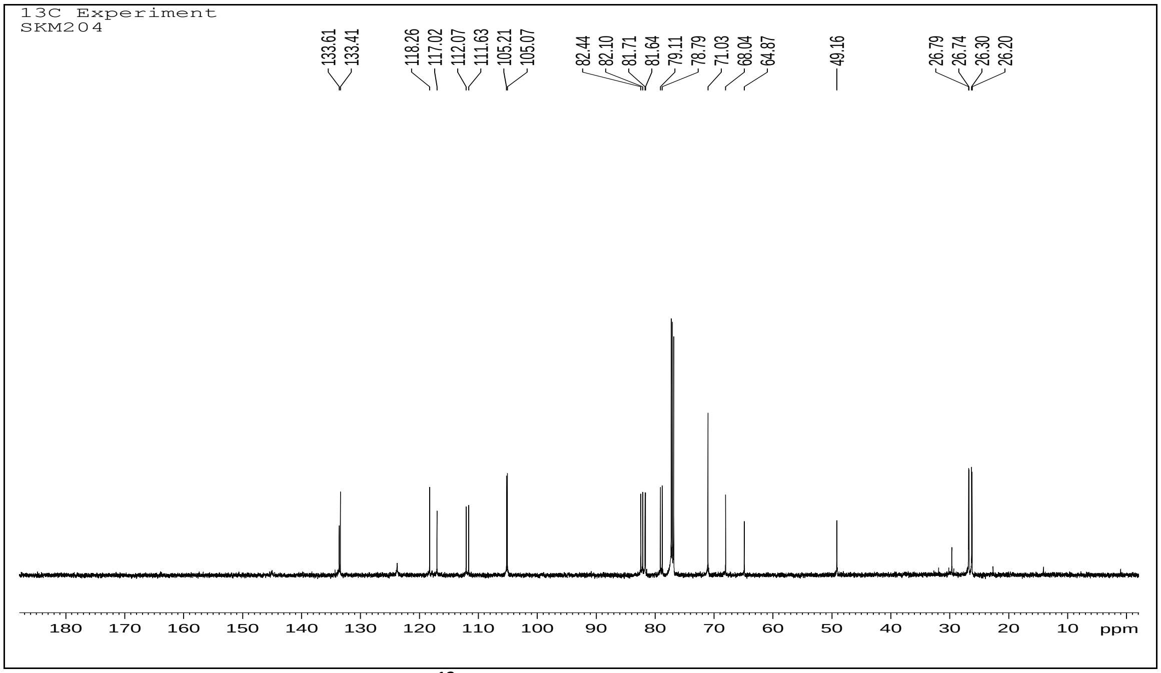

^{13}C spectrum of compound 3a in $CDCl_3$

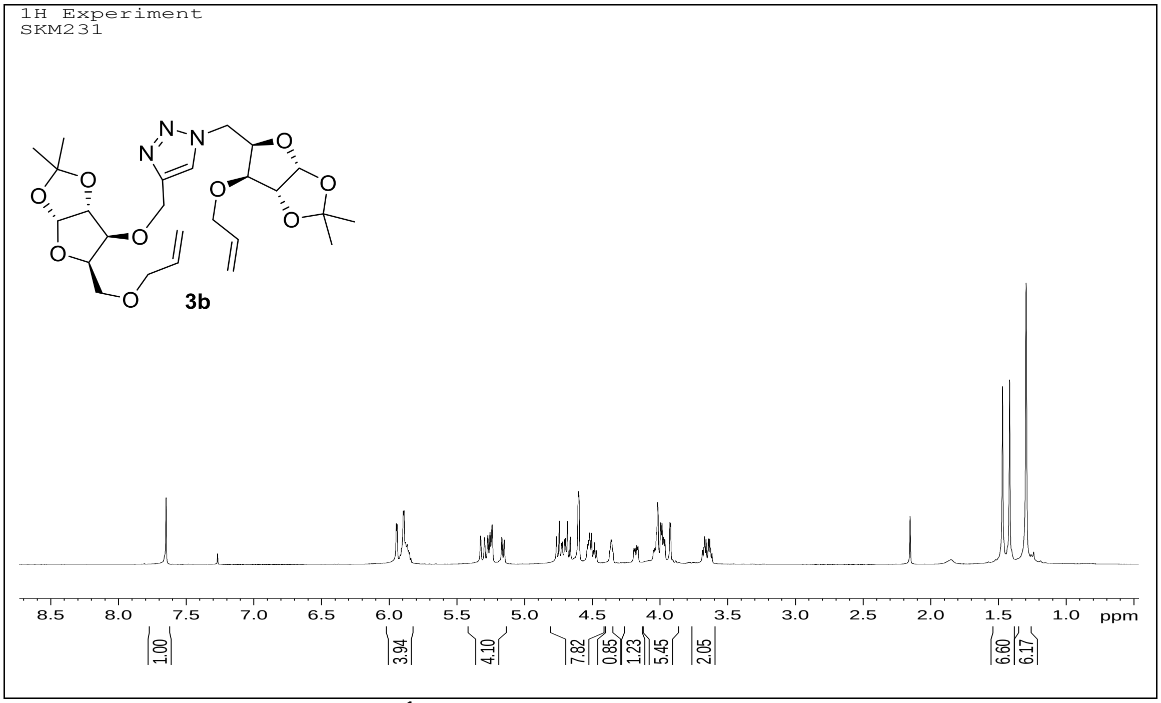

^{1}H NMR spectrum of compound 3b in $CDCl_3$

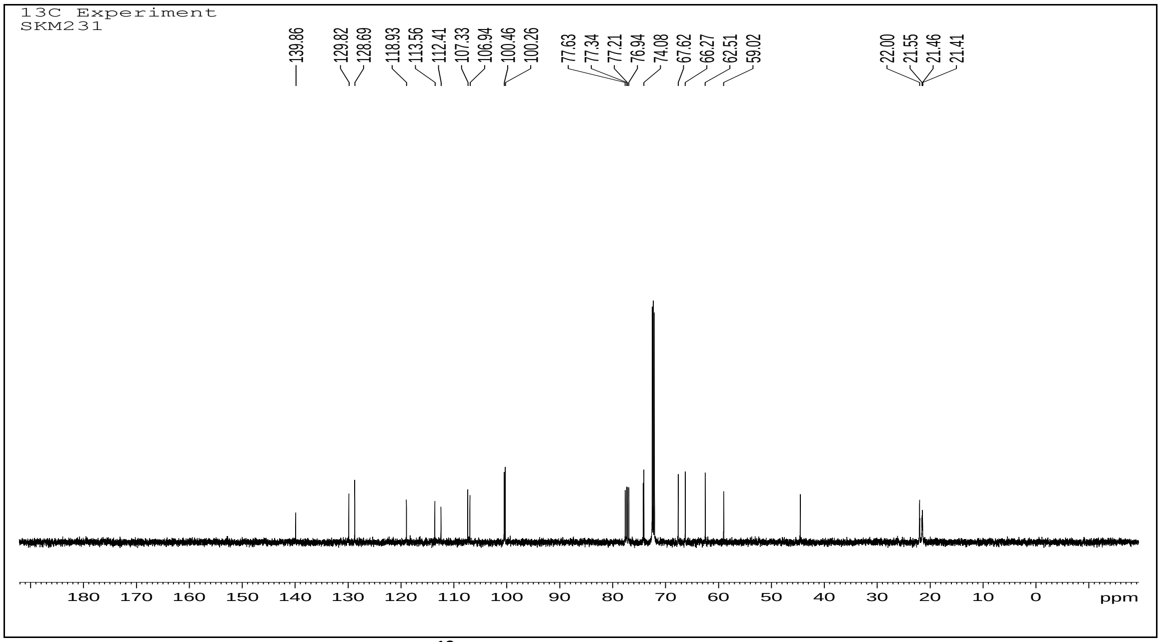

^{13}C spectrum of compound 3b in $CDCl_3$

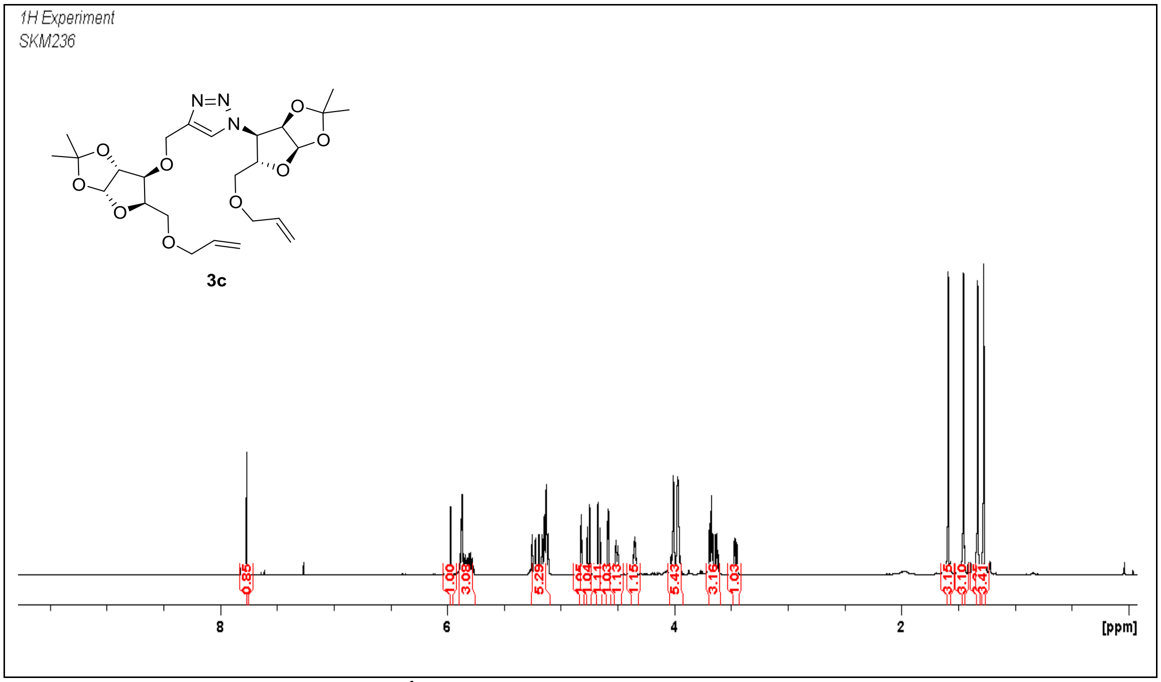

^{1}H NMR spectrum of compound 3c in $CDCl_3$

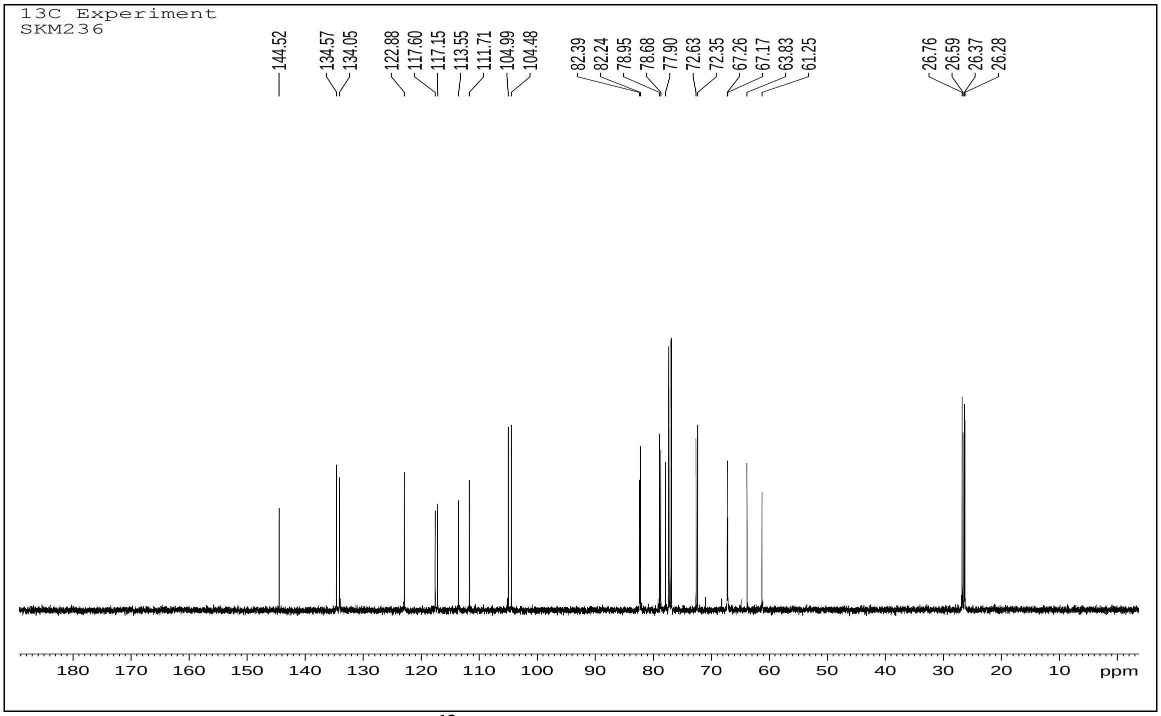

^{13}C spectrum of compound 3c in $CDCl_3$

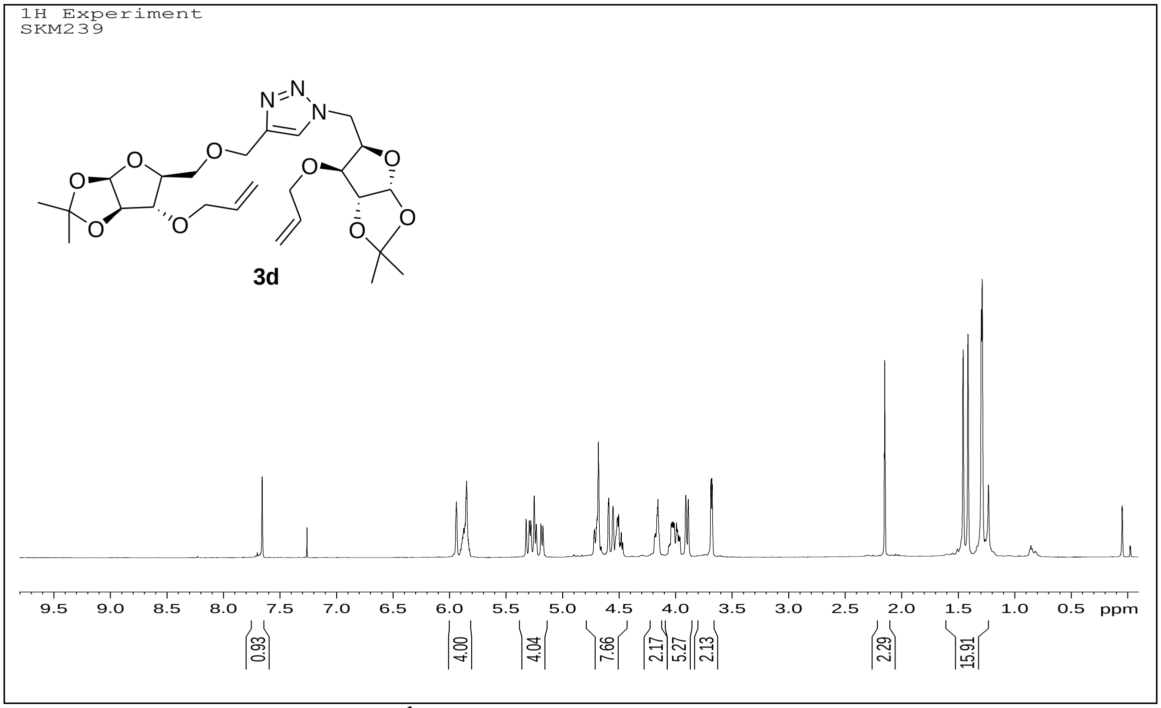

^{1}H NMR spectrum of compound 3d in $CDCl_3$

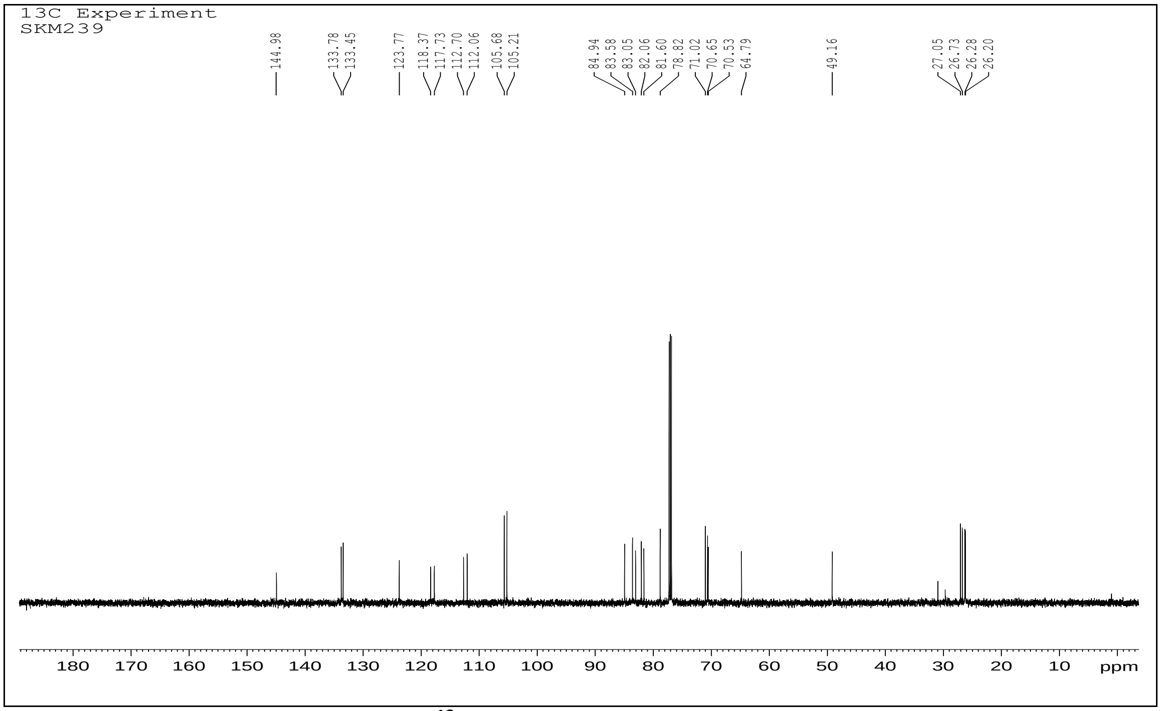

^{13}C spectrum of compound 3d in $CDCl_3$

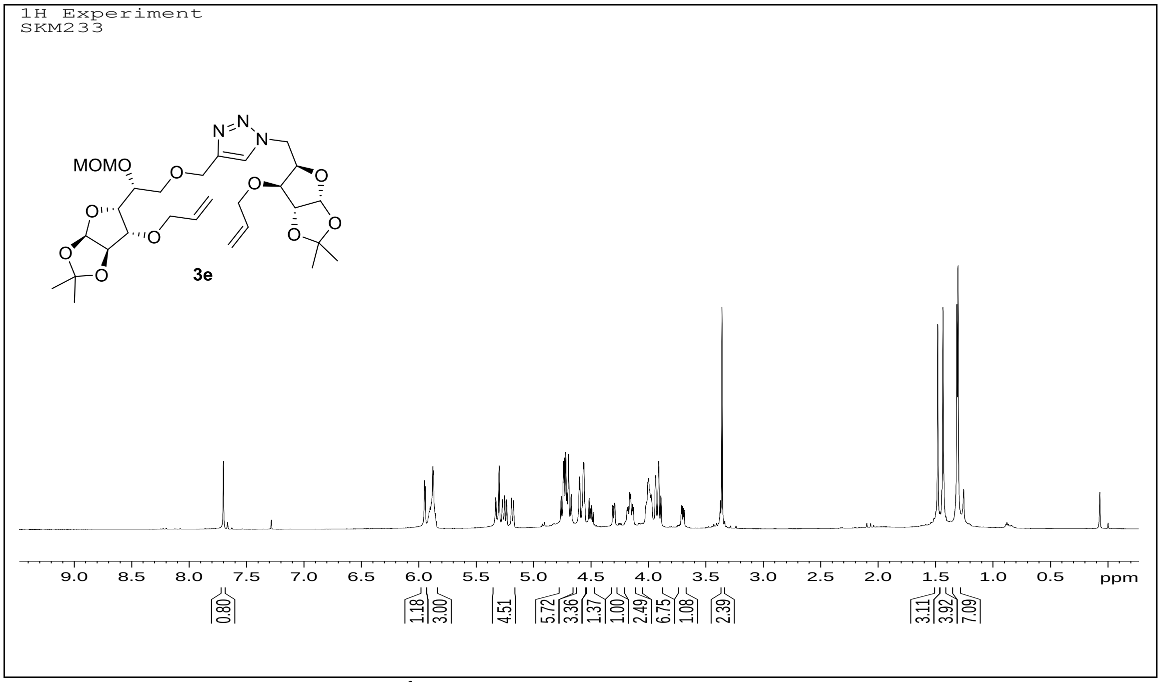

^{1}H NMR spectrum of compound 3e in $CDCl_3$

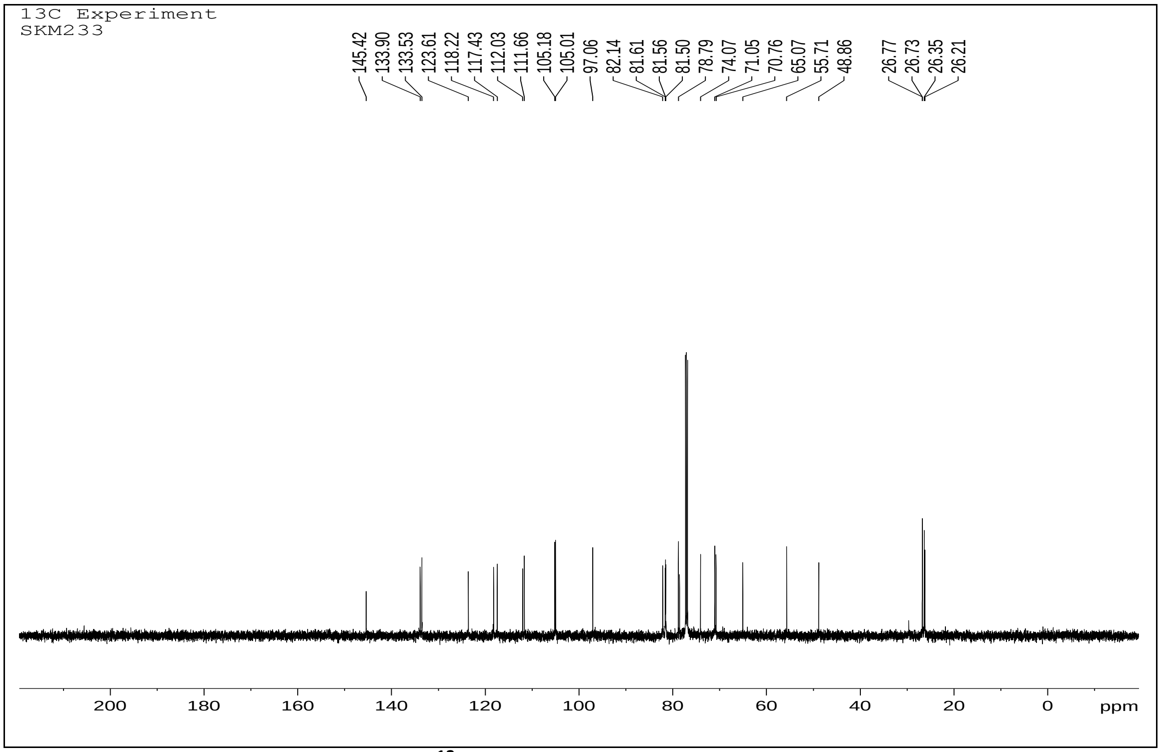

^{13}C spectrum of compound 3e in $CDCl_3$

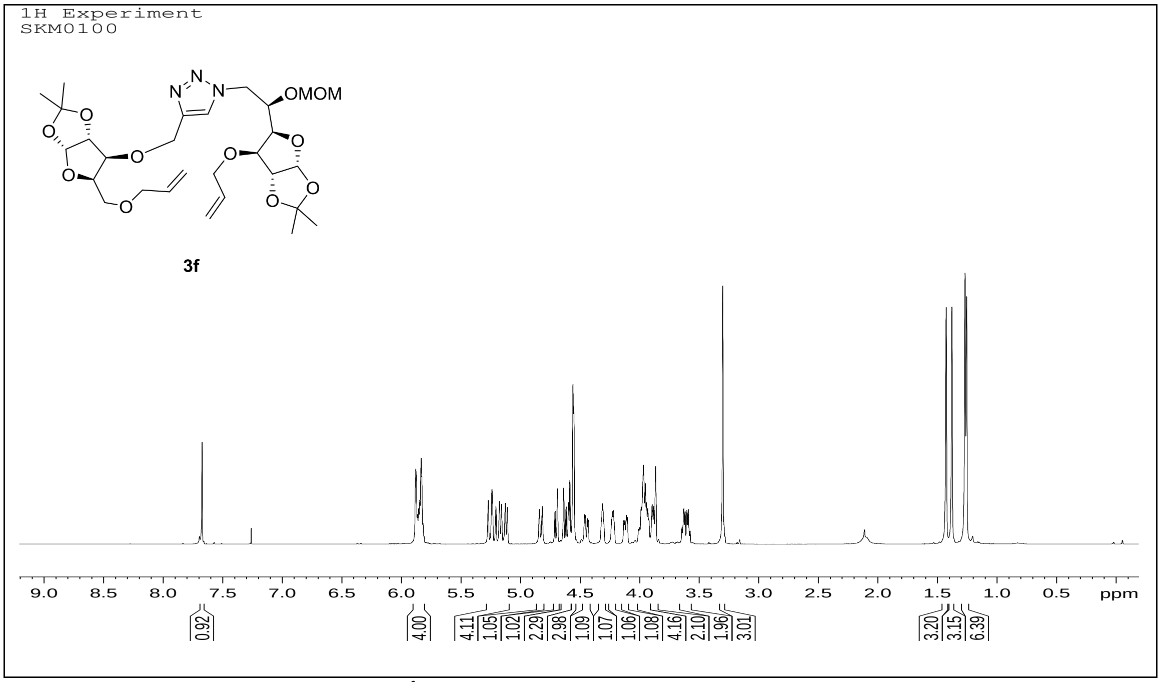

^{1}H NMR spectrum of compound 3f in $CDCl_3$

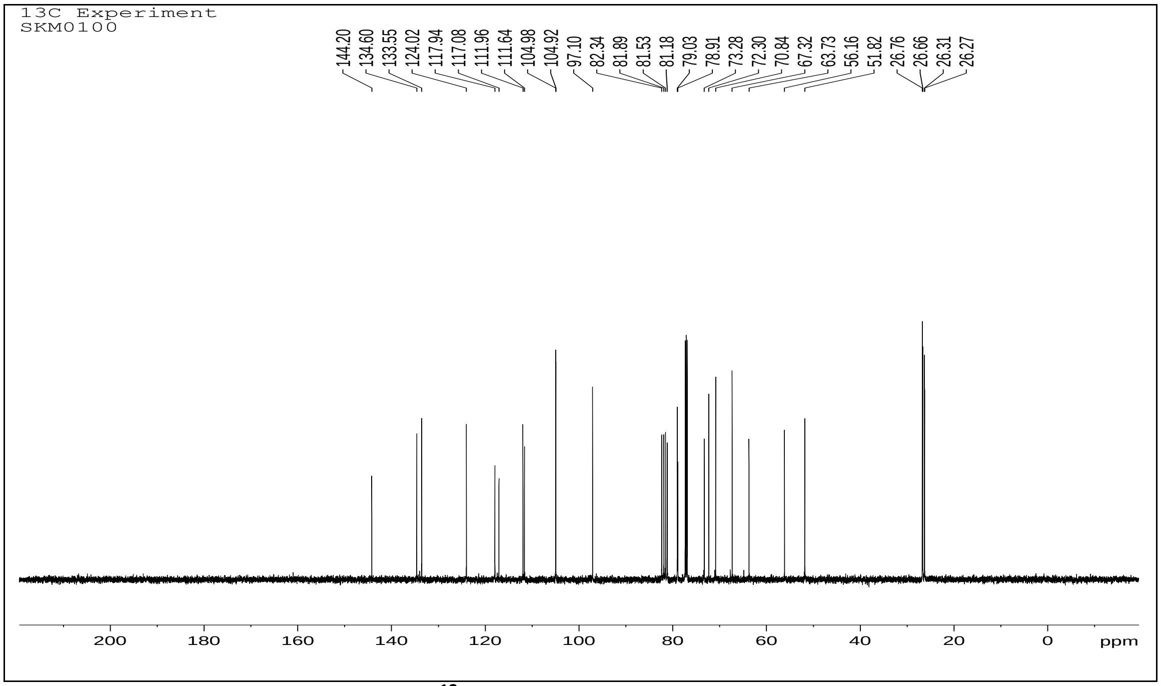

^{13}C spectrum of compound 3f in $CDCl_3$

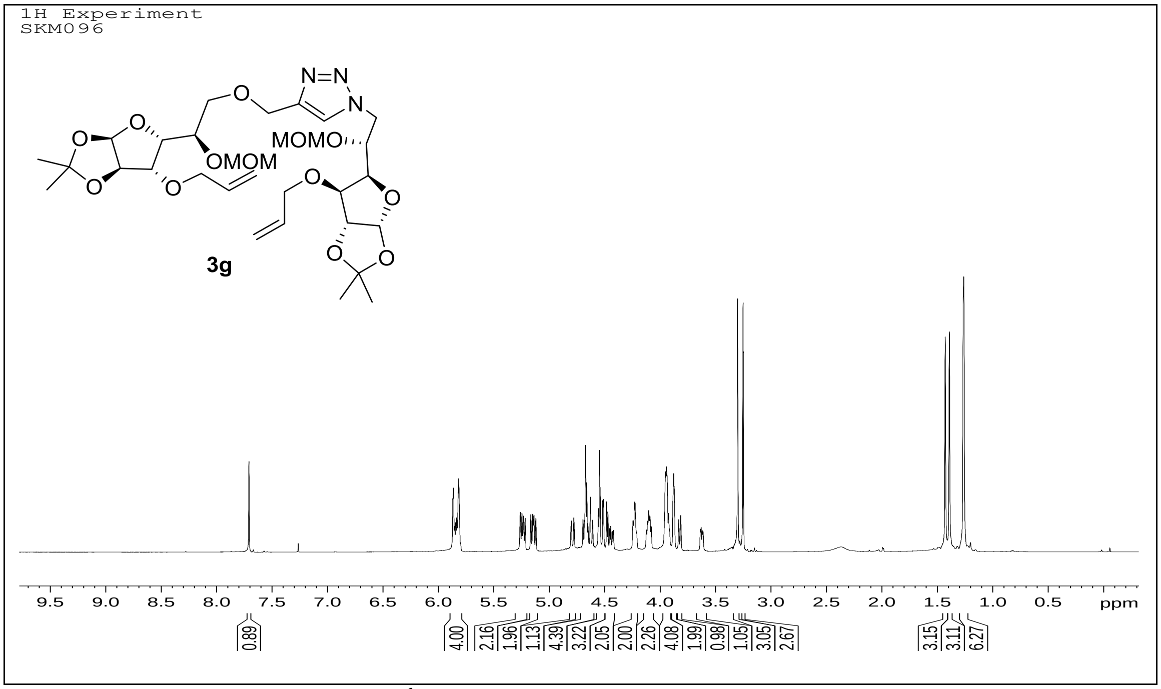

^{1}H NMR spectrum of compound 3g in $CDCl_3$

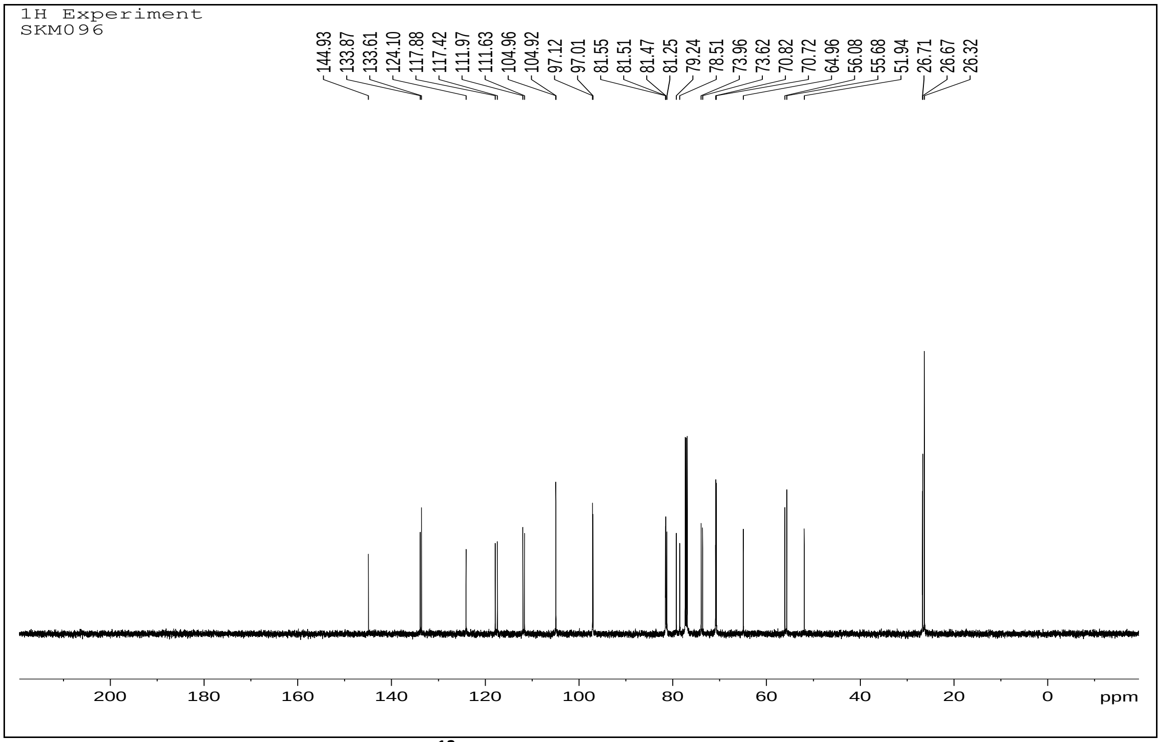

^{13}C spectrum of compound 3g in $CDCl_3$

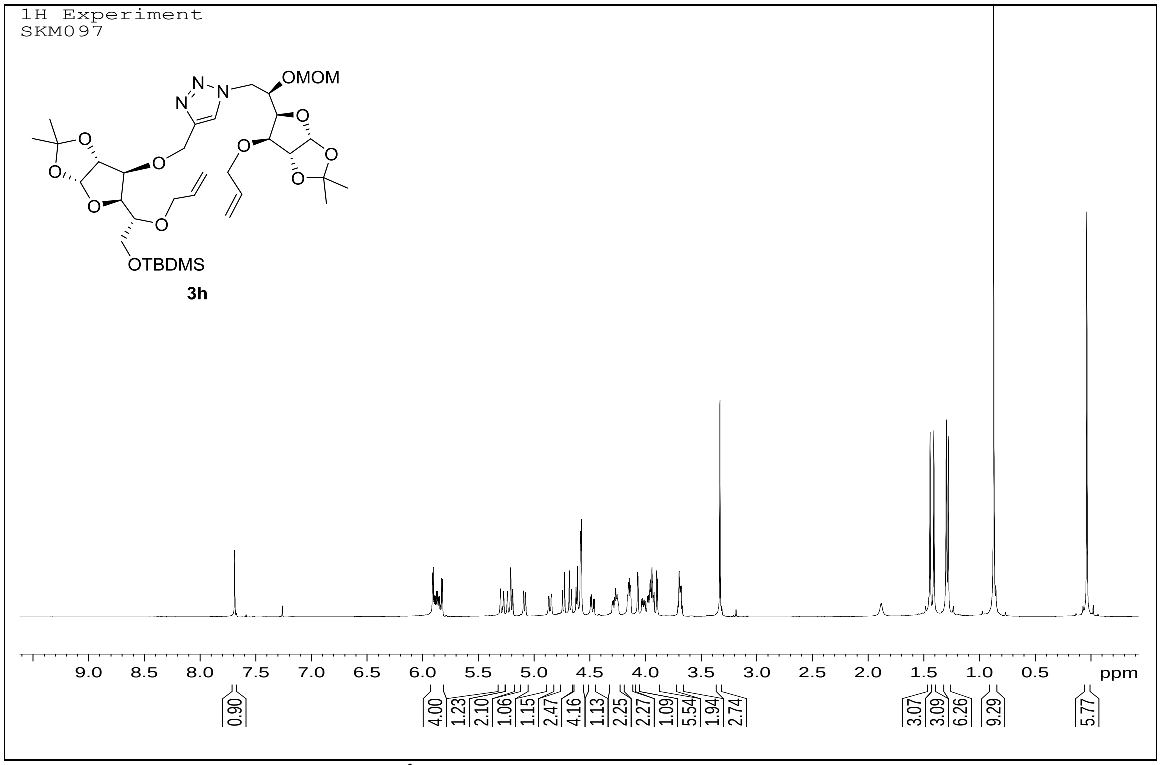

^{1}H NMR spectrum of compound 3h in $CDCl_3$

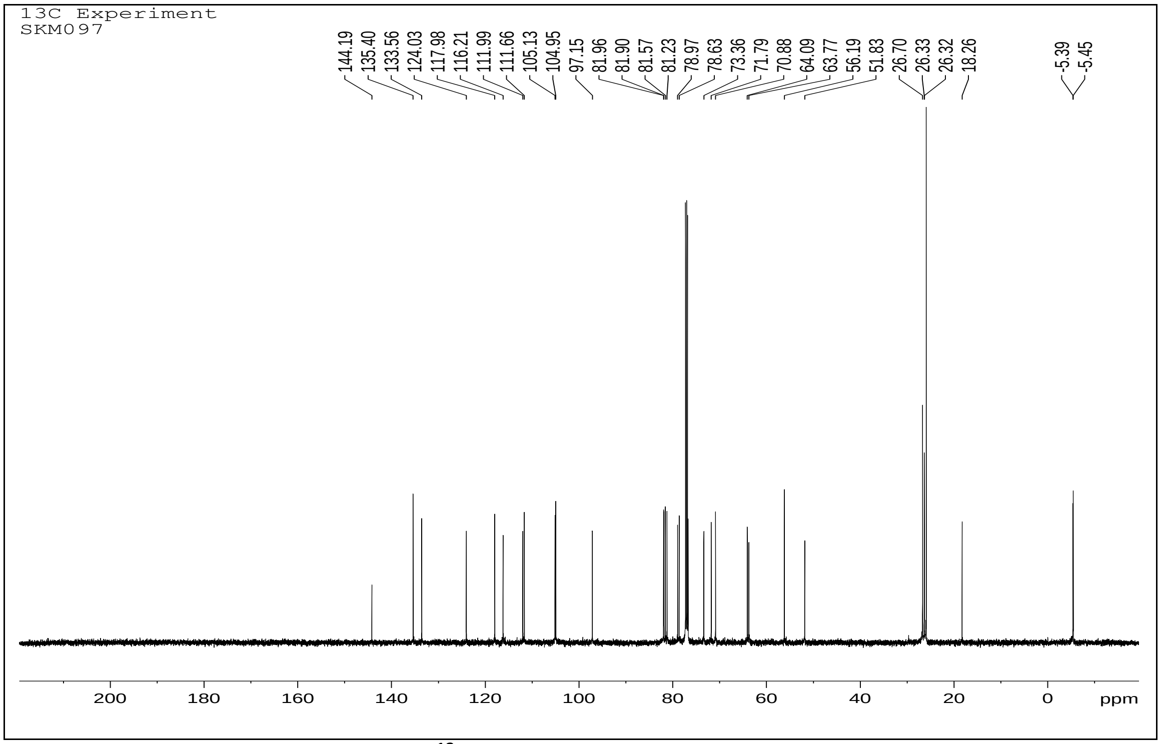

^{13}C spectrum of compound 3h in $CDCl_3$

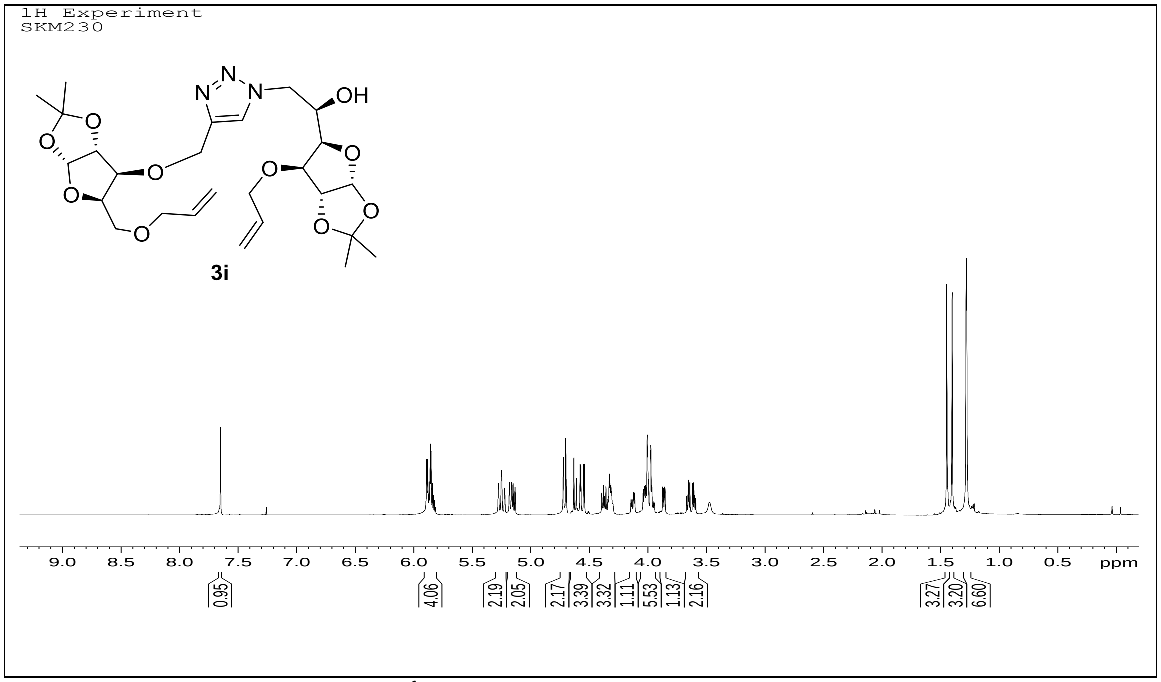

^{1}H NMR spectrum of compound 3i in $CDCl_3$

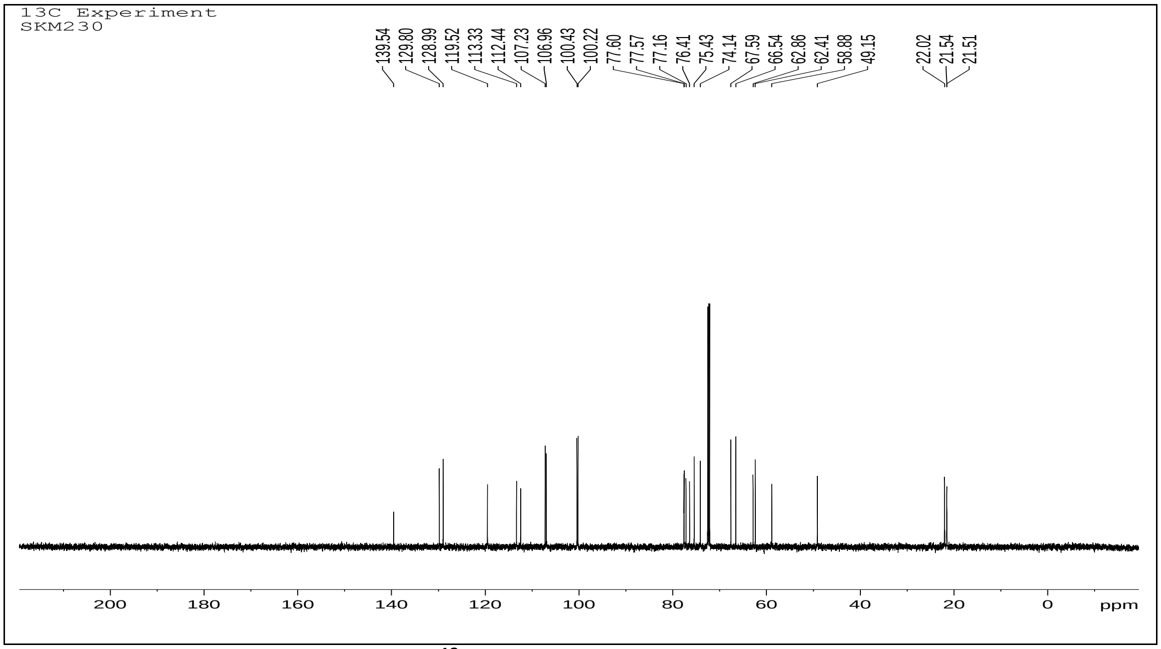

^{13}C spectrum of compound 3i in $CDCl_3$

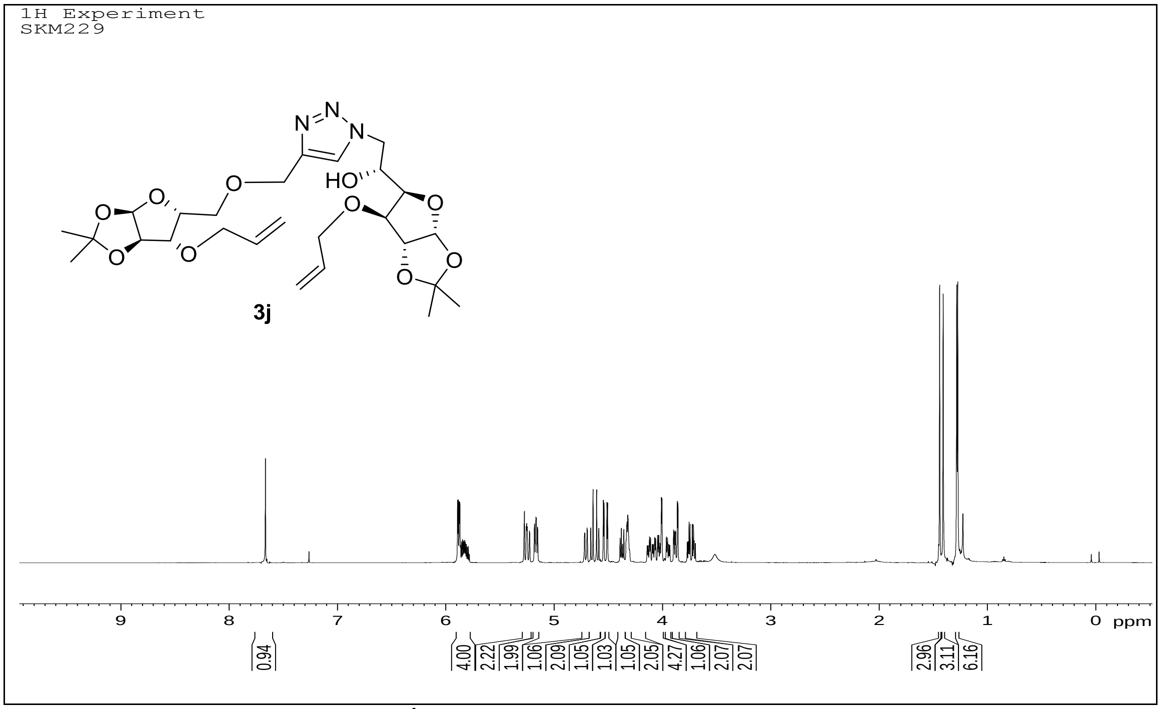

^{1}H NMR spectrum of compound 3j in $CDCl_3$

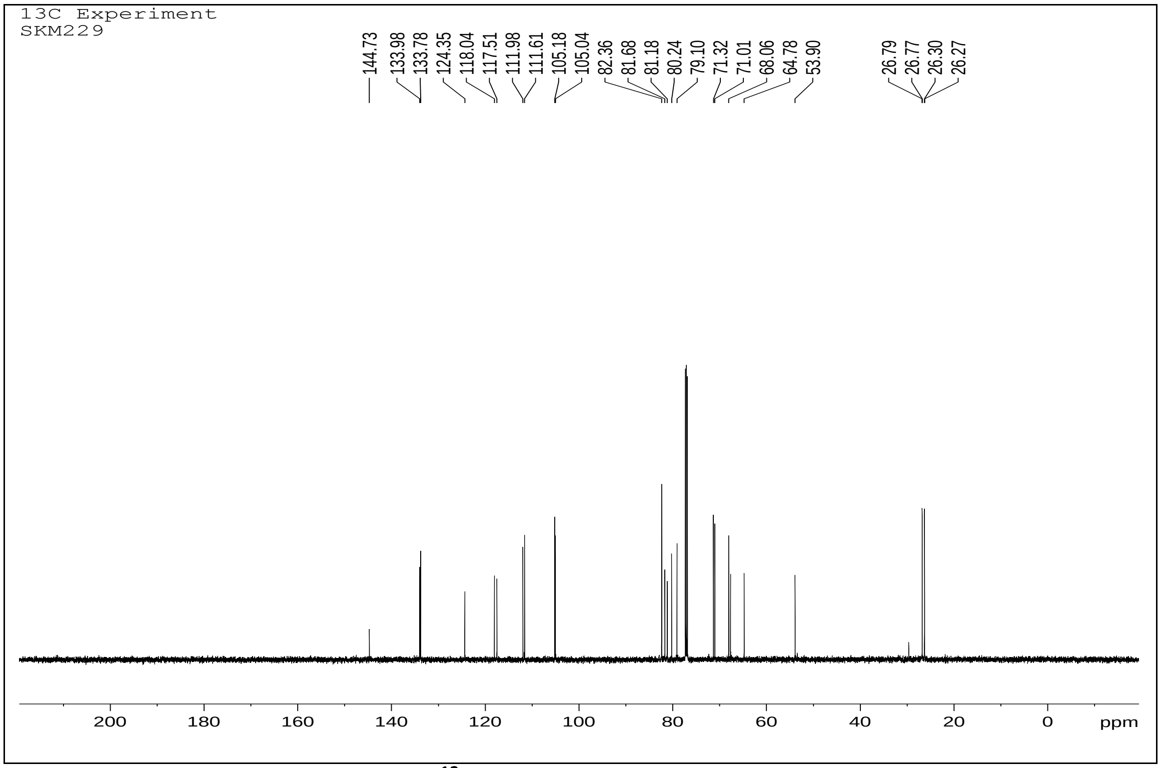

^{13}C spectrum of compound 3j in $CDCl_3$

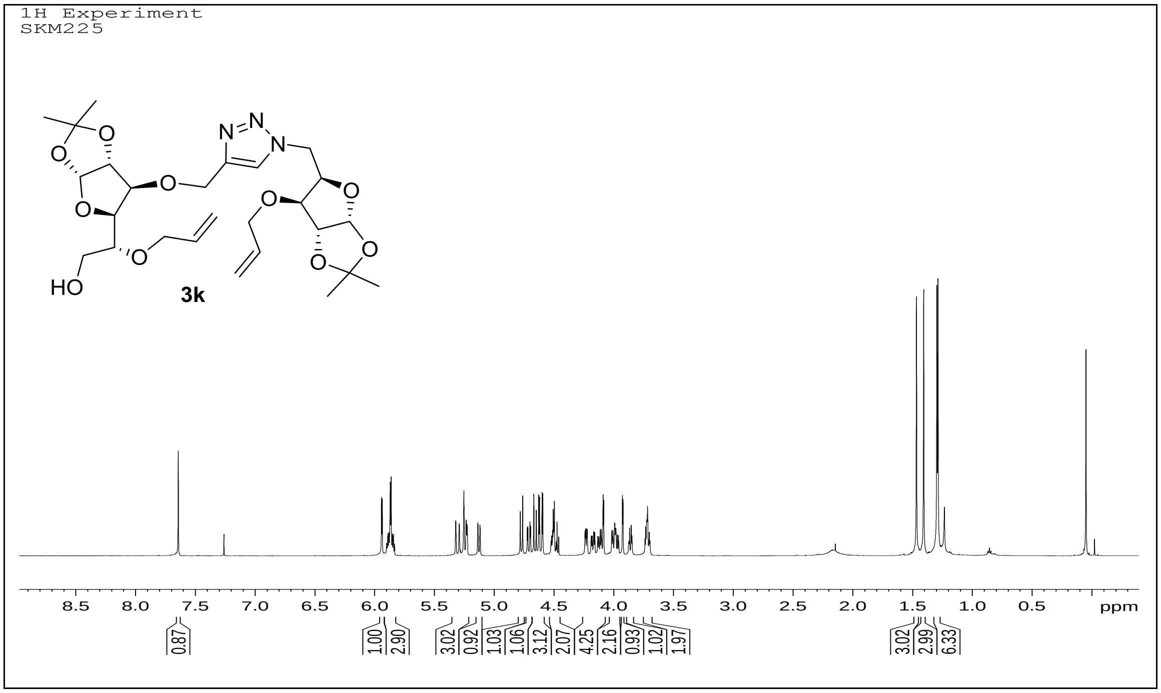

^{1}H NMR spectrum of compound 3k in $CDCl_3$

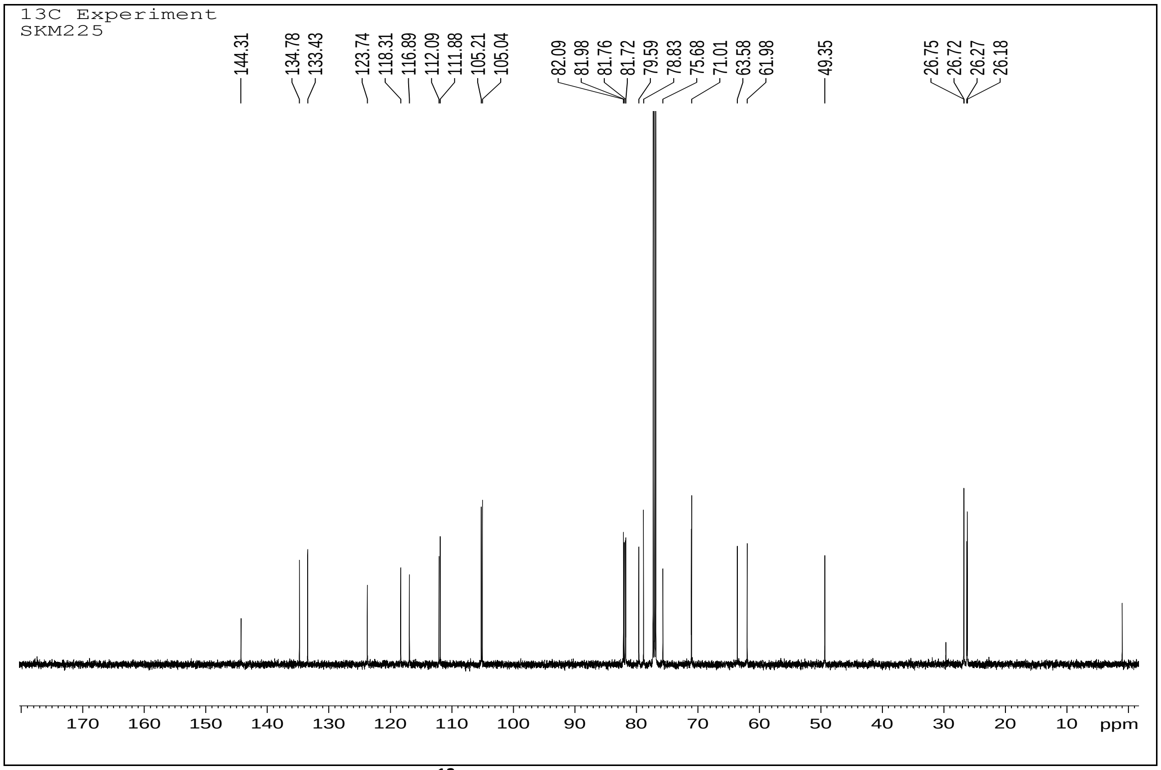

^{13}C spectrum of compound 3k in $CDCl_3$

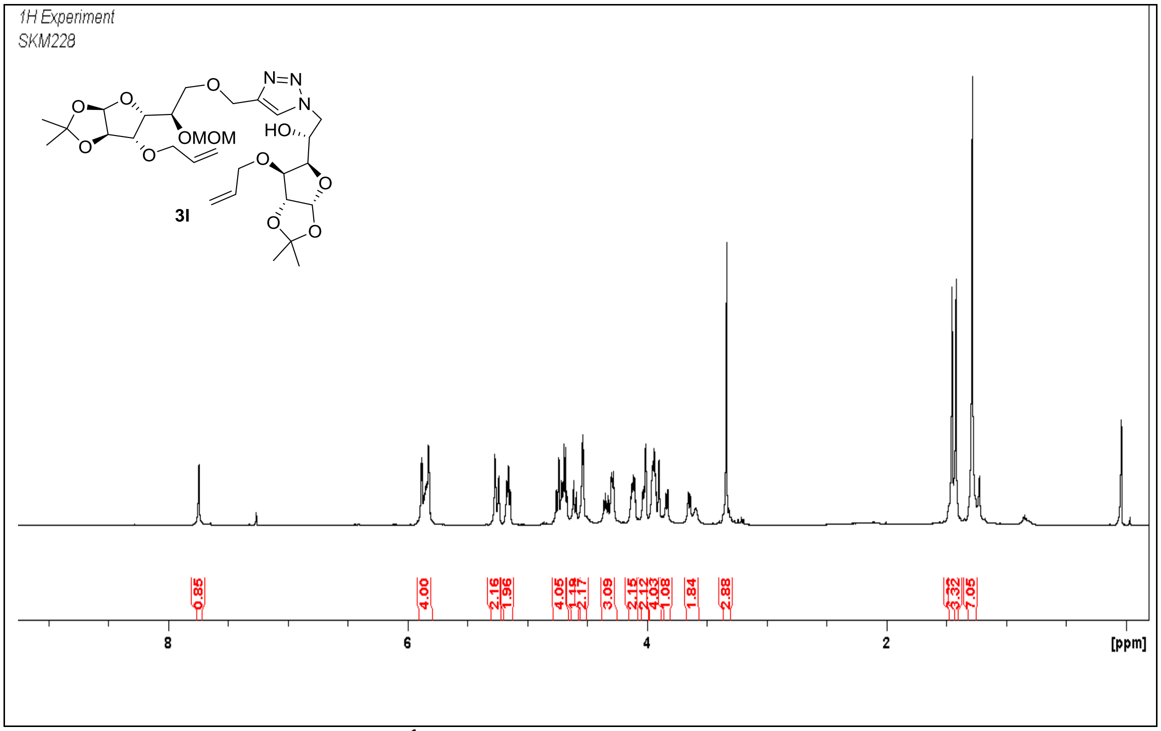

^{1}H NMR spectrum of compound 3l in $CDCl_3$

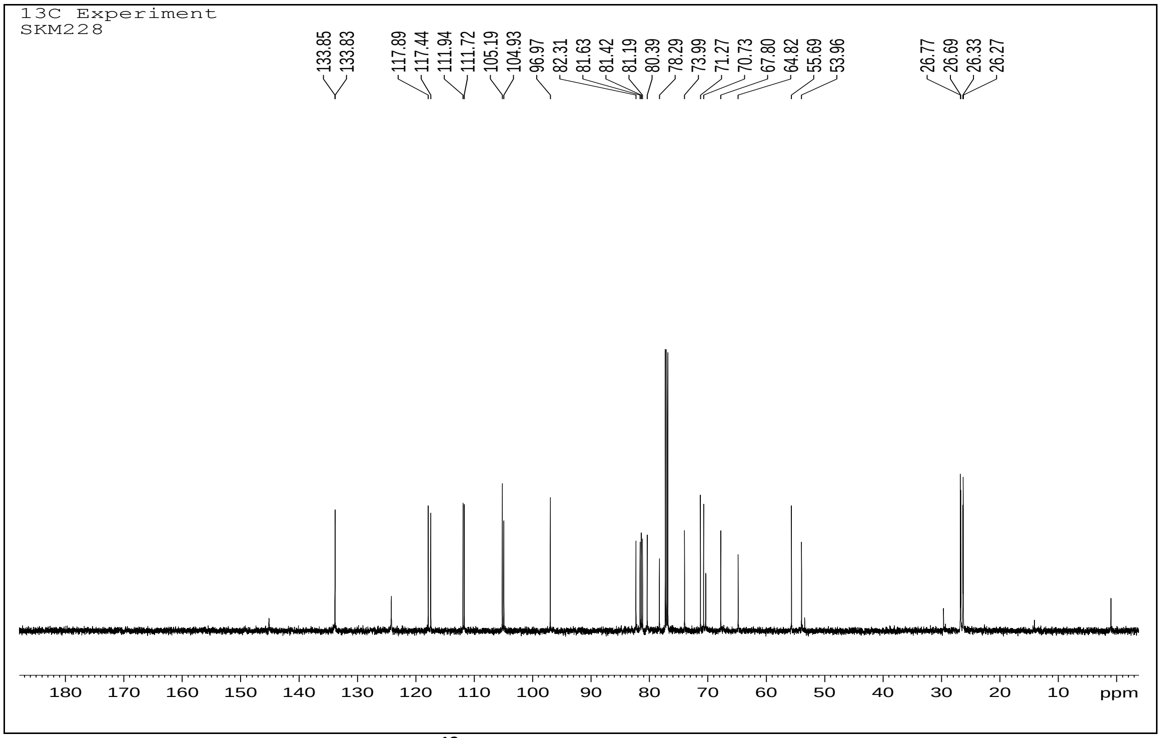

^{13}C spectrum of compound 3l in $CDCl_3$

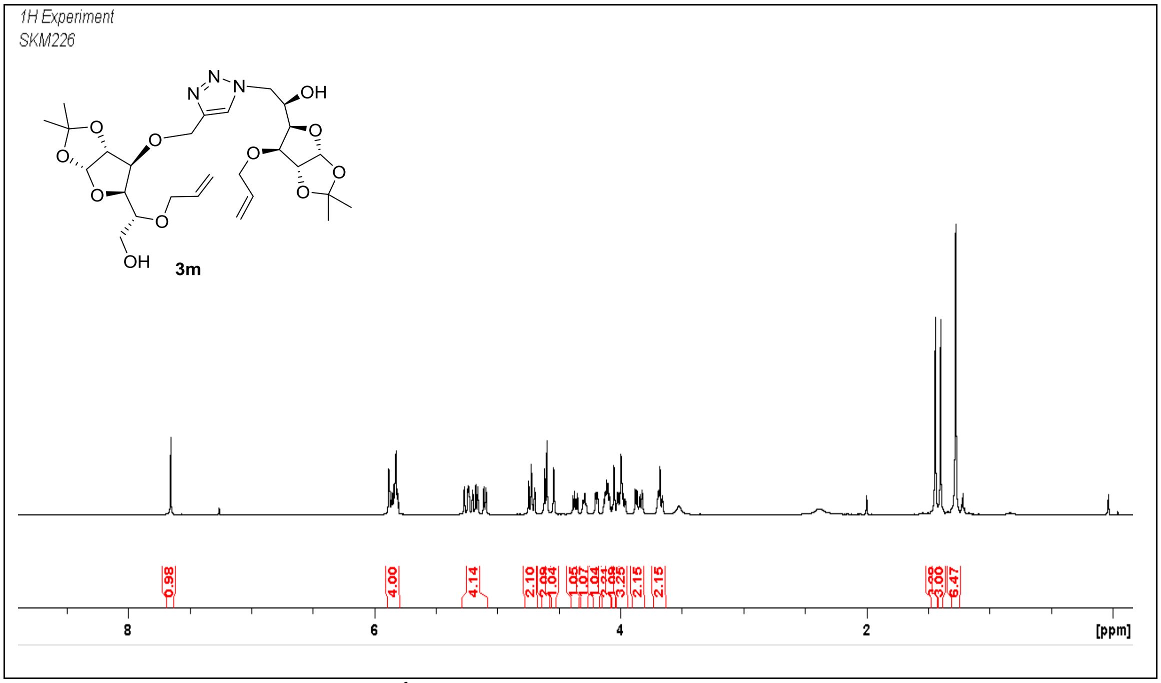

^{1}H NMR spectrum of compound 3m in $CDCl_3$

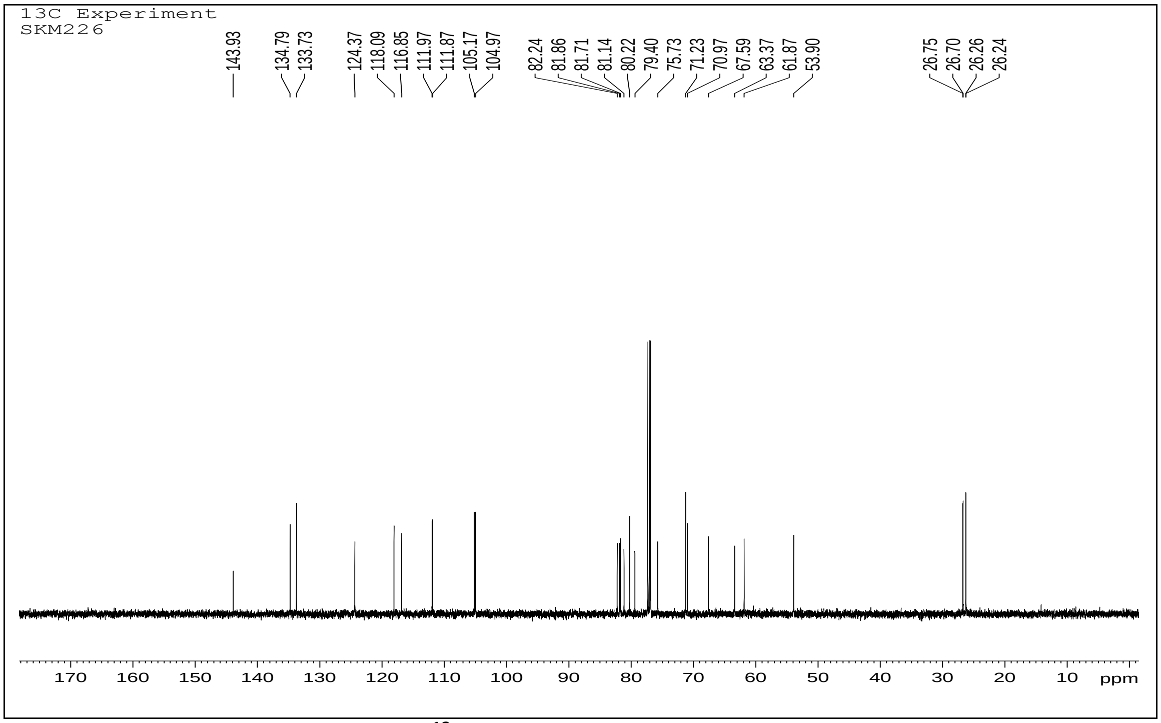

^{13}C spectrum of compound 3m in $CDCl_3$

NMR spectrum of RCM Products (S82-S95)

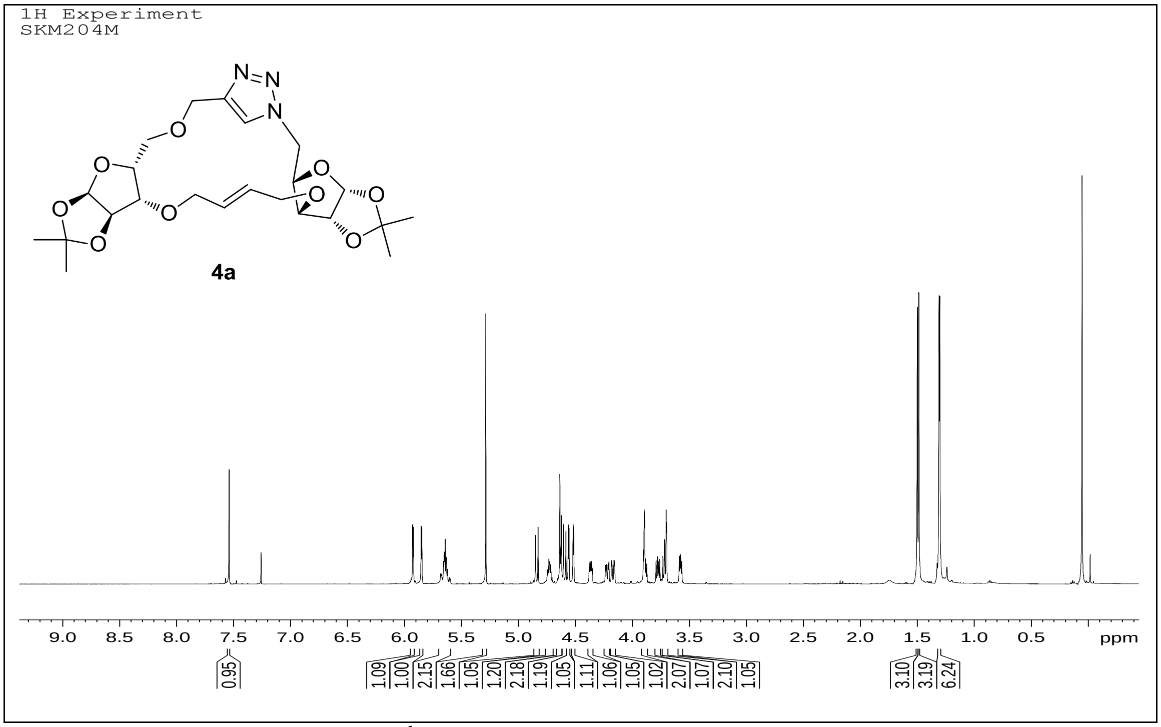

^{1}H NMR spectrum of compound 4a in $CDCl_3$

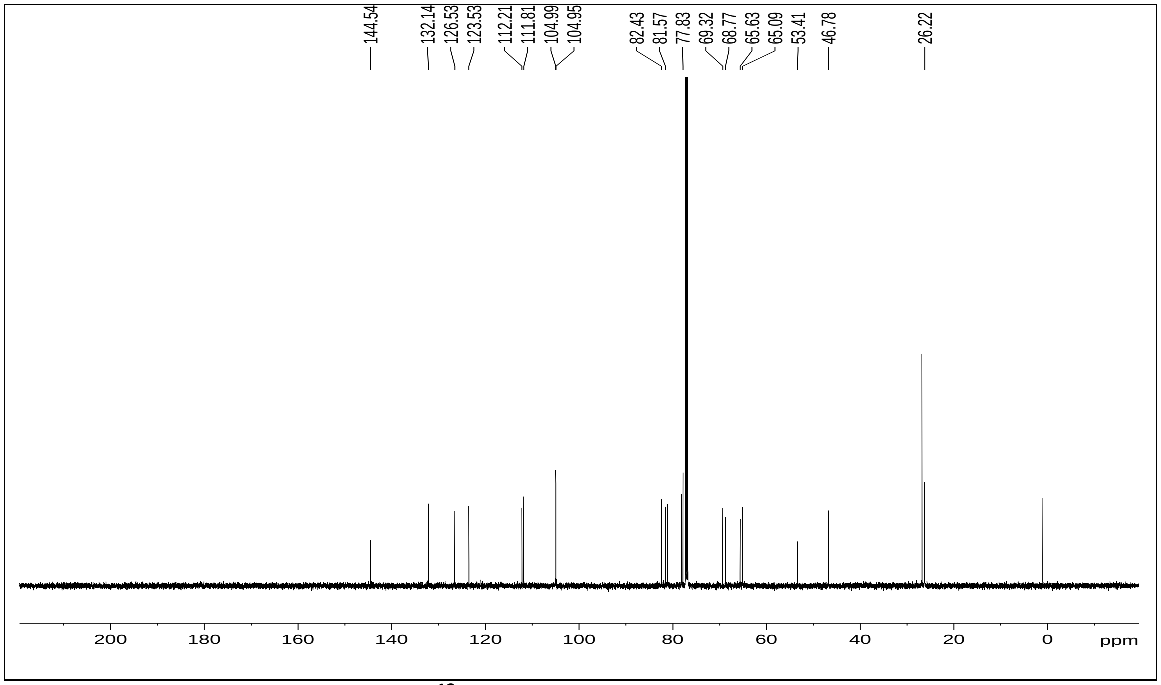

^{13}C spectrum of compound 4a in $CDCl_3$

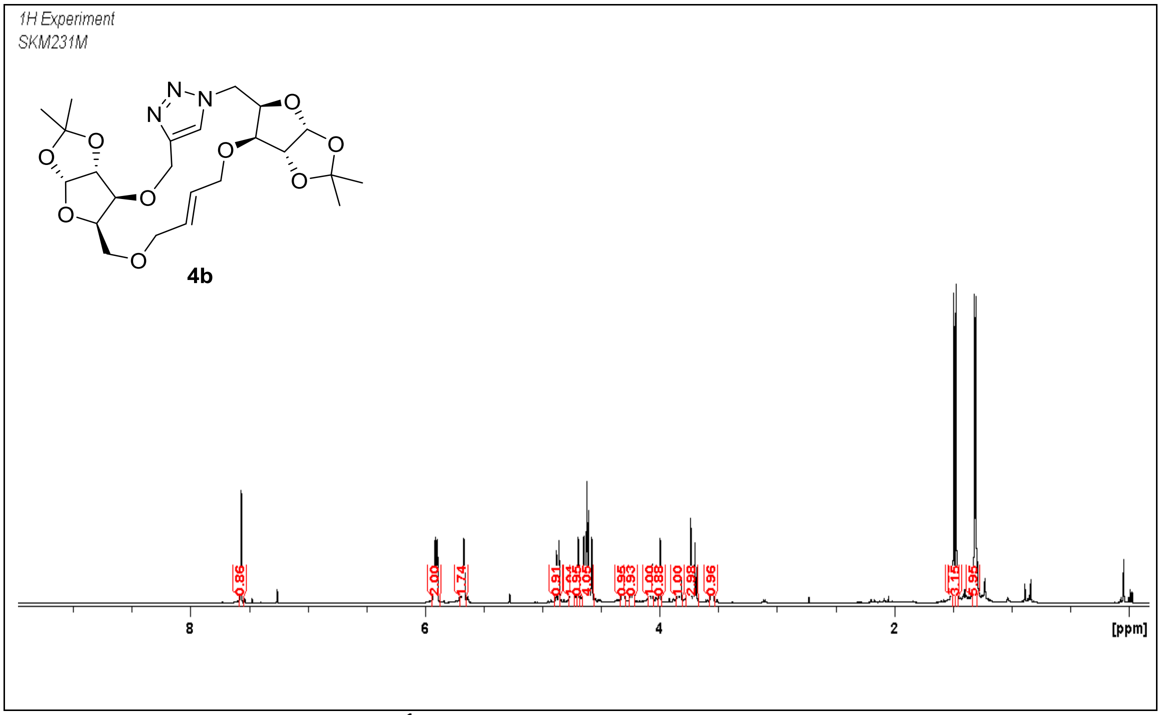

^{1}H NMR spectrum of compound 4b in $CDCl_3$

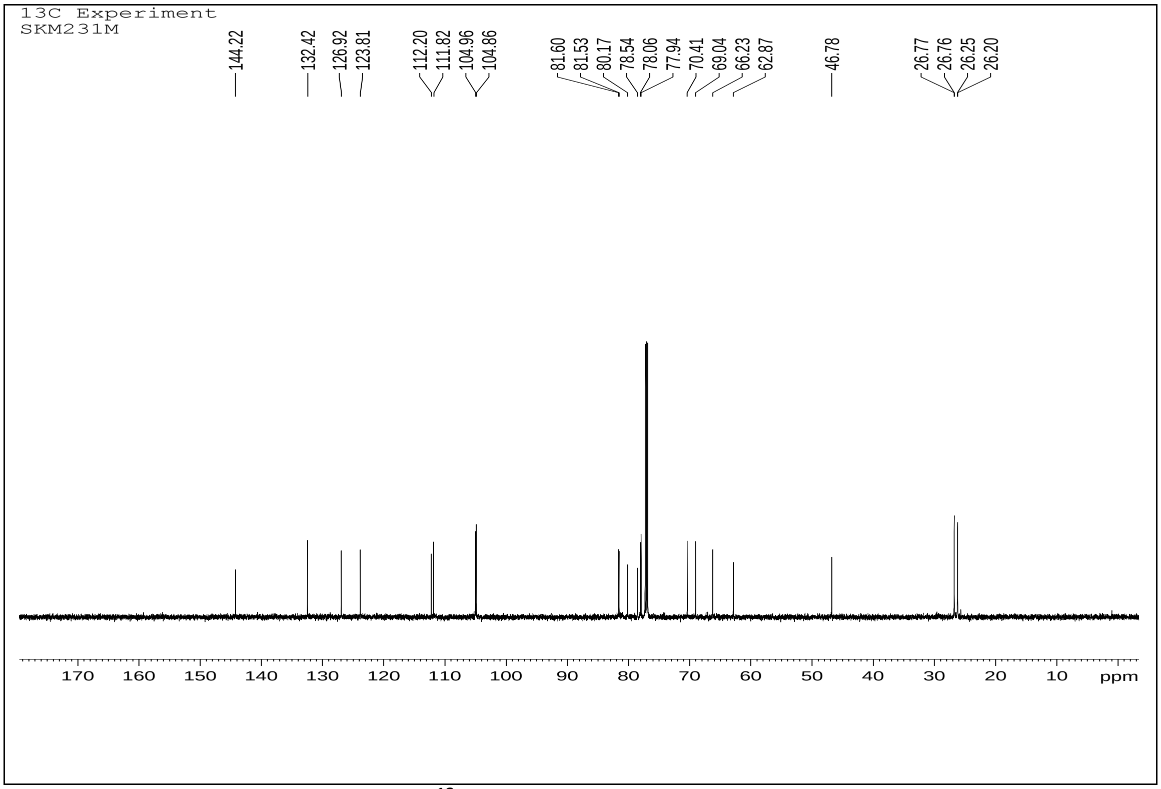

^{13}C spectrum of compound 4b in $CDCl_3$

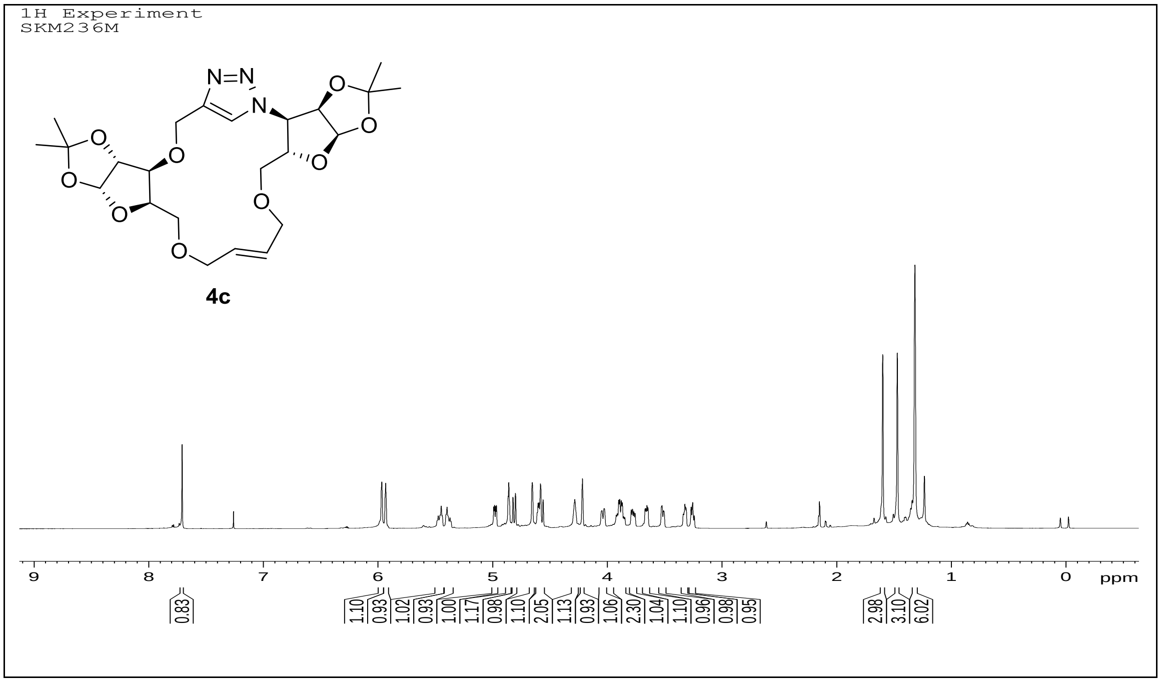

^{1}H NMR spectrum of compound 4c in $CDCl_3$

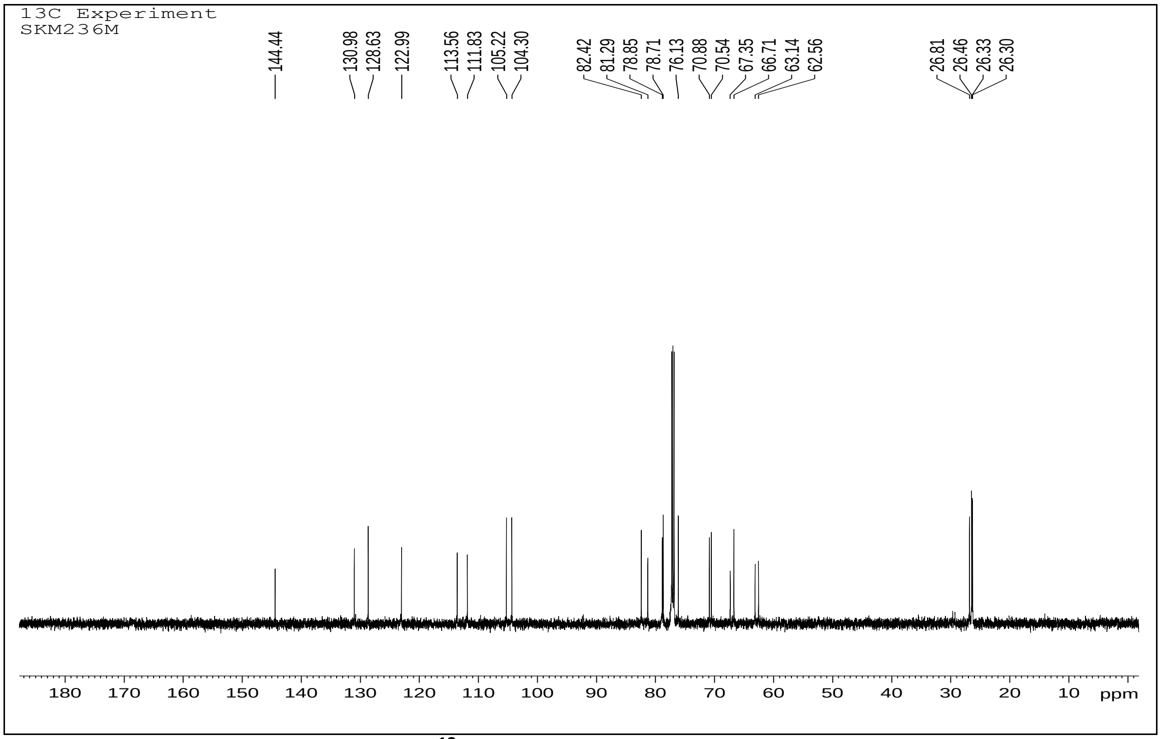

^{13}C spectrum of compound 4c in $CDCl_3$

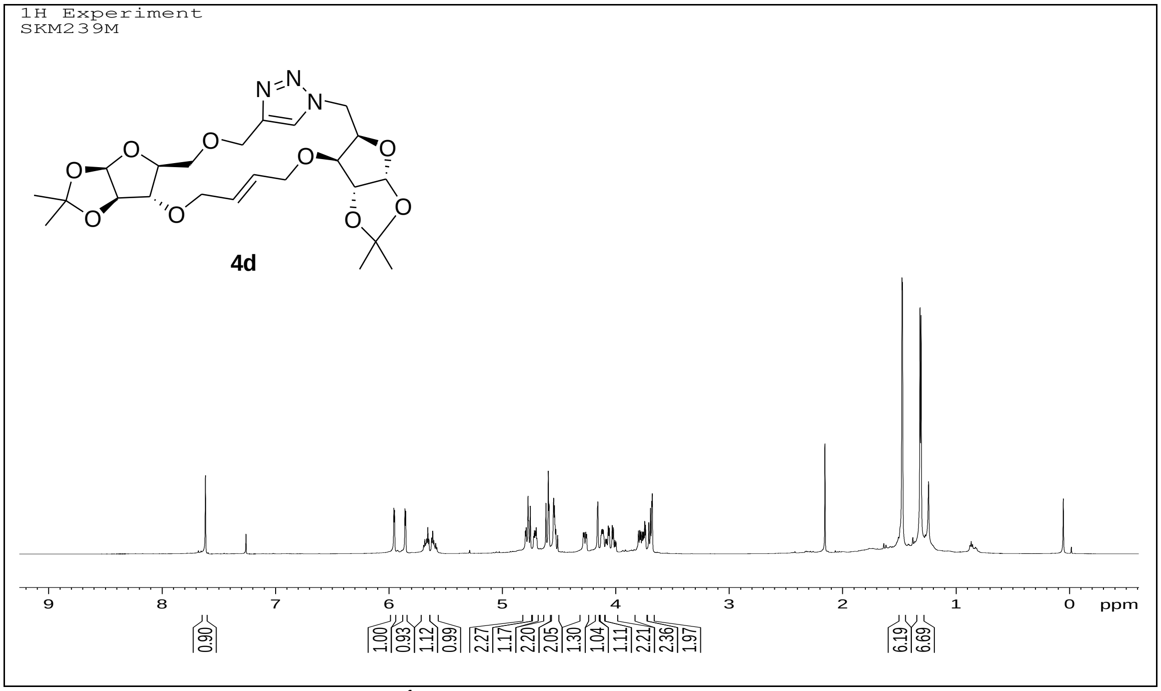

^{1}H NMR spectrum of compound 4d in $CDCl_3$

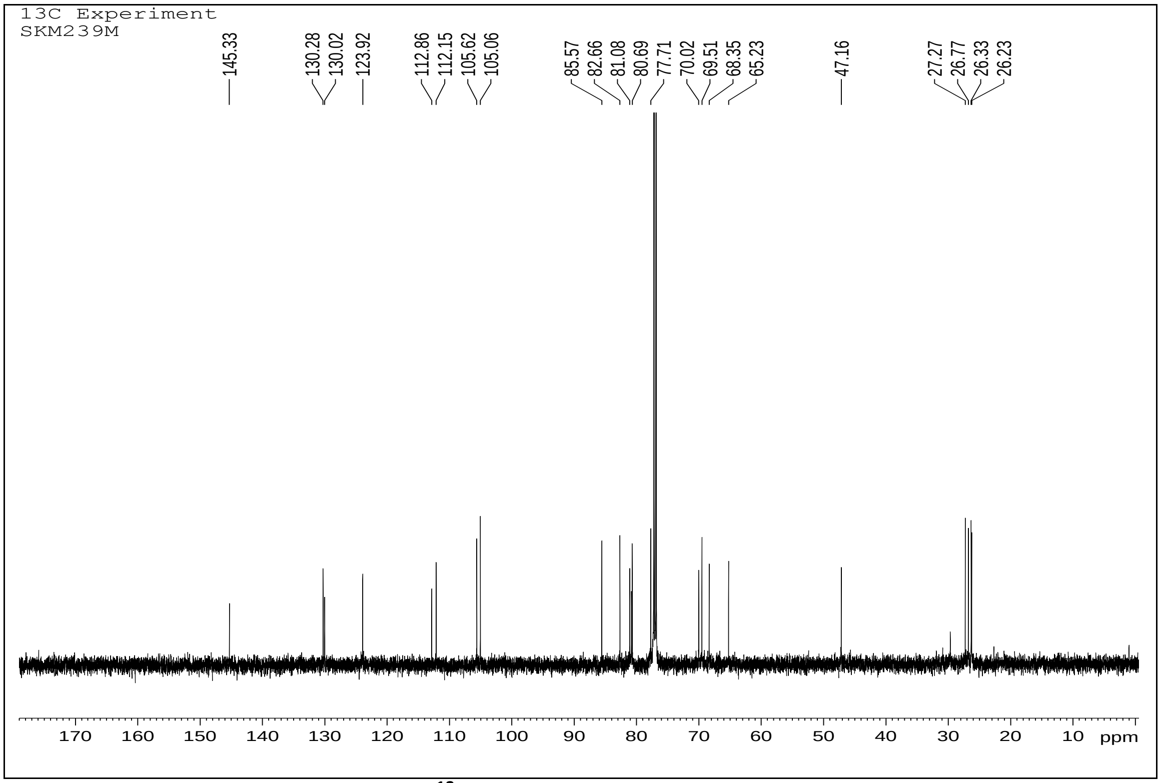

^{13}C spectrum of compound 4d in $CDCl_3$

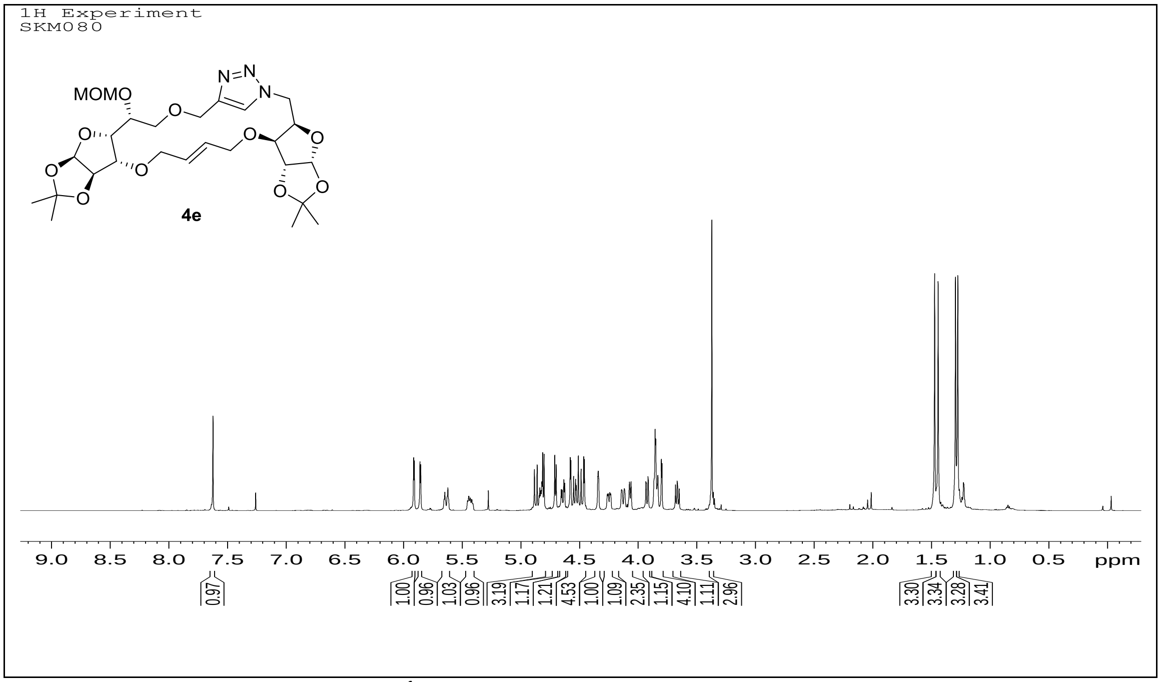

^{1}H NMR spectrum of compound 4e in $CDCl_3$

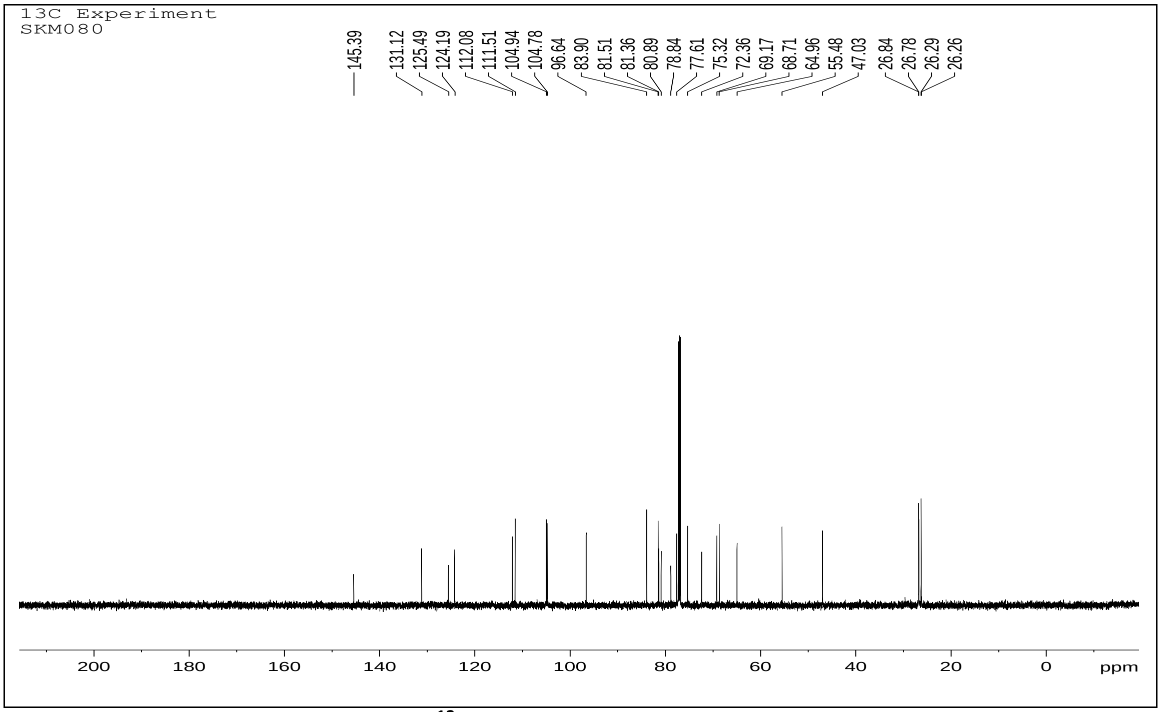

^{13}C spectrum of compound 4e in $CDCl_3$

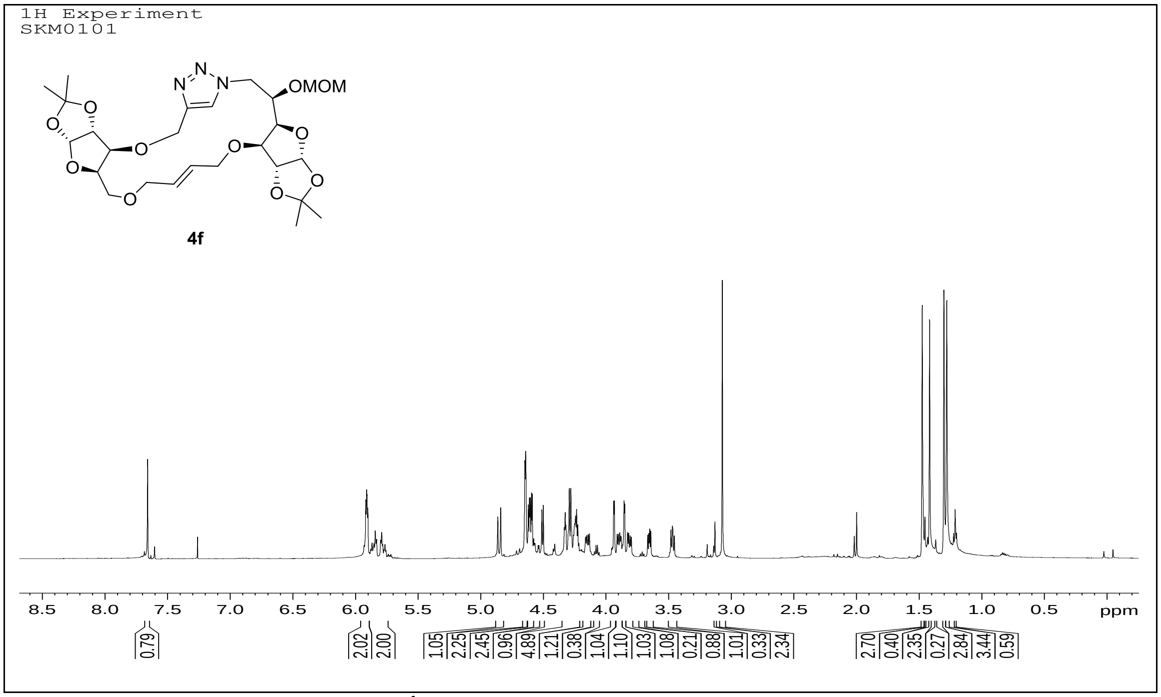

^{1}H NMR spectrum of compound 4f in $CDCl_3$

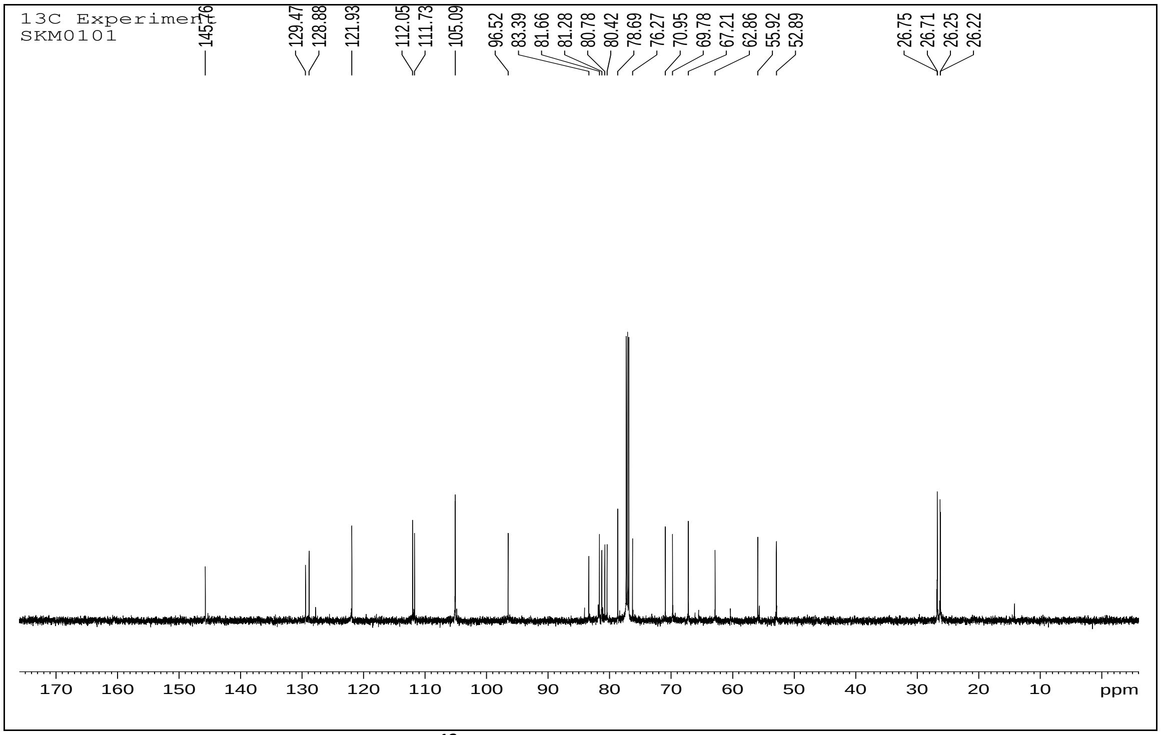

^{13}C spectrum of compound 4f in $CDCl_3$

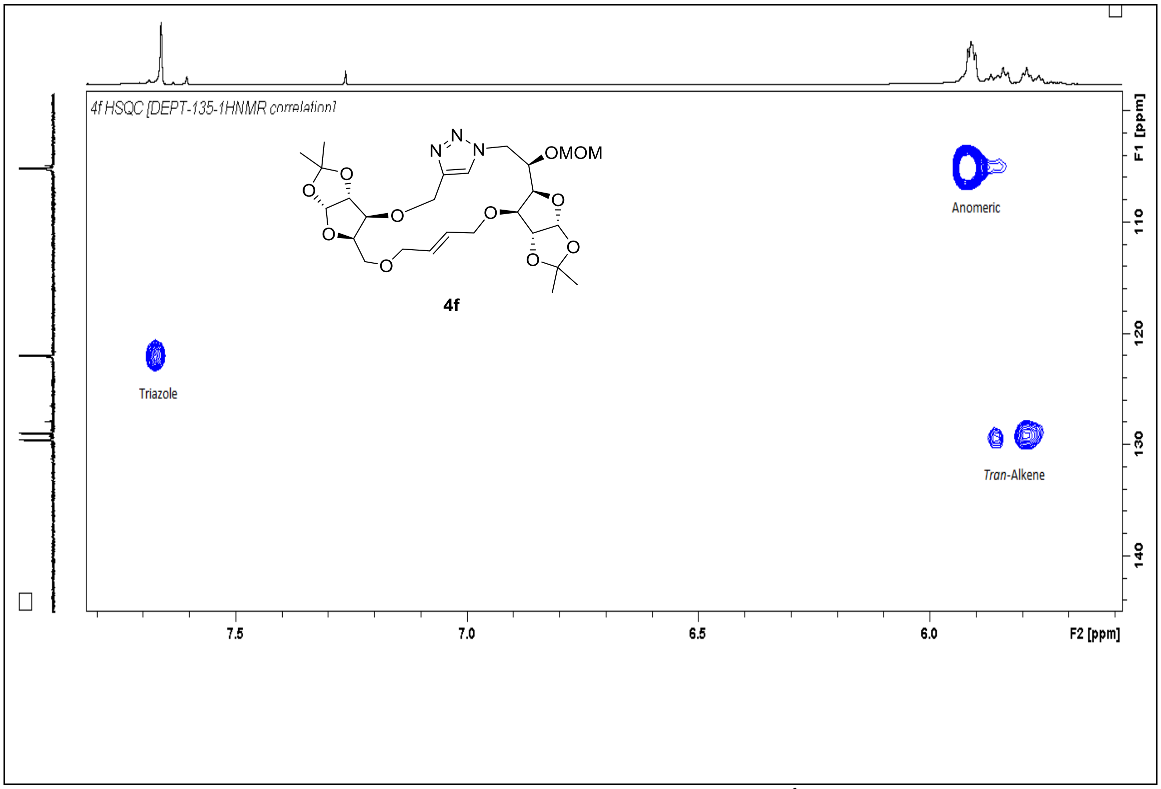

HSQC spectrum of compound 4f (DEPT-135-^{1}H NMR) in $CDCl_3$

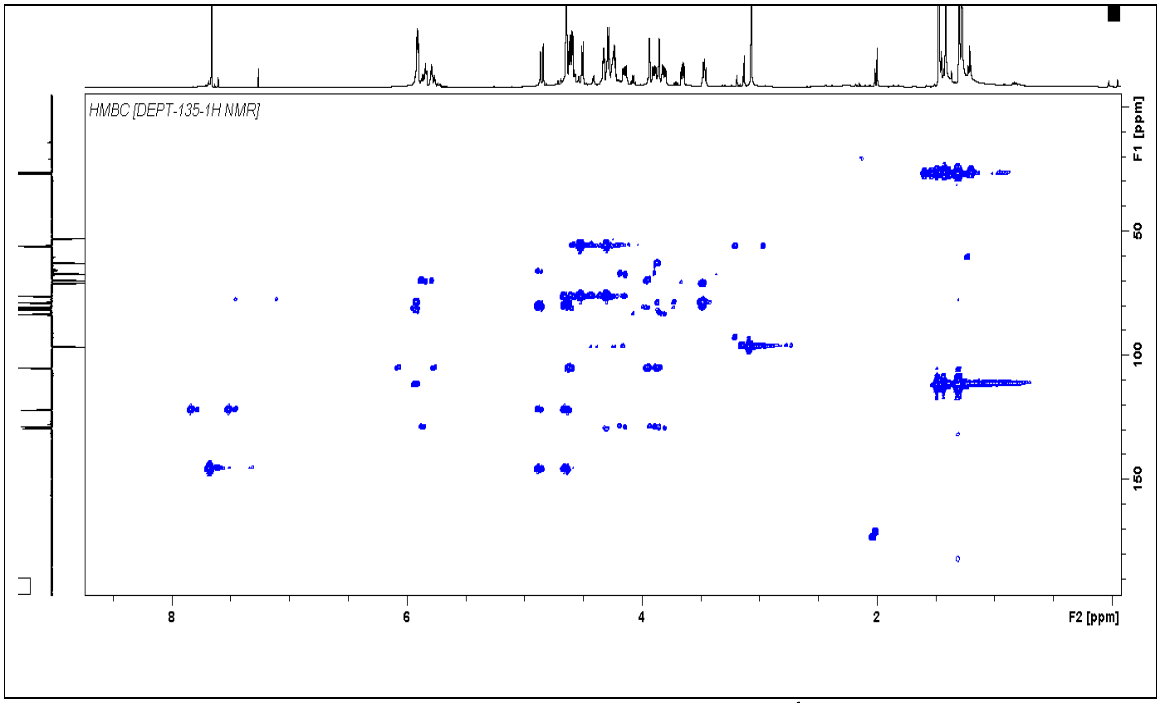

HMBC spectrum of compound 4f (DEPT-135-^{1}H NMR) in $CDCl_3$

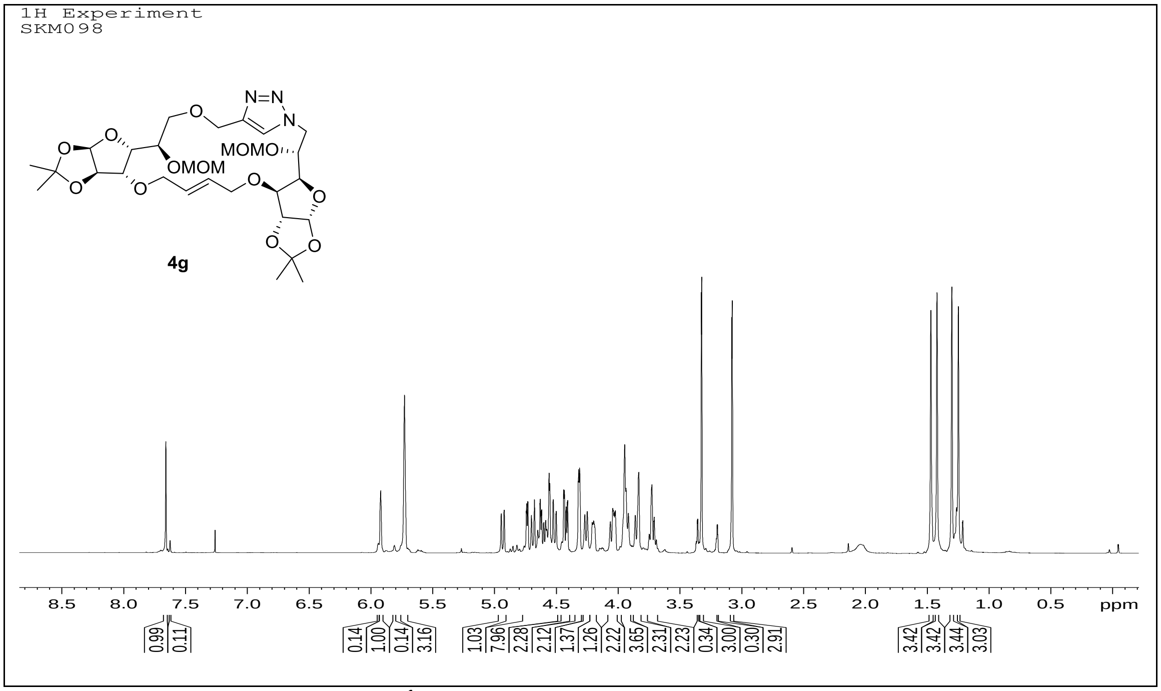

^{1}H NMR spectrum of compound 4g in $CDCl_3$

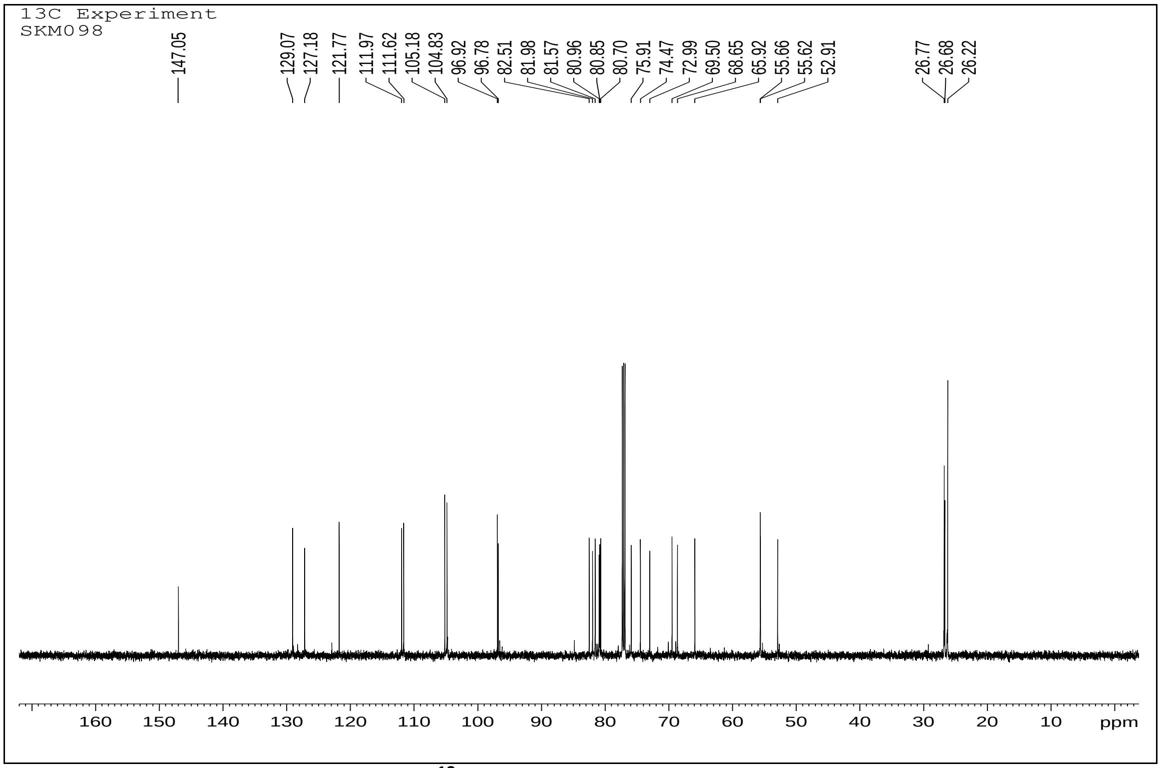

^{13}C spectrum of compound 4g in $CDCl_3$

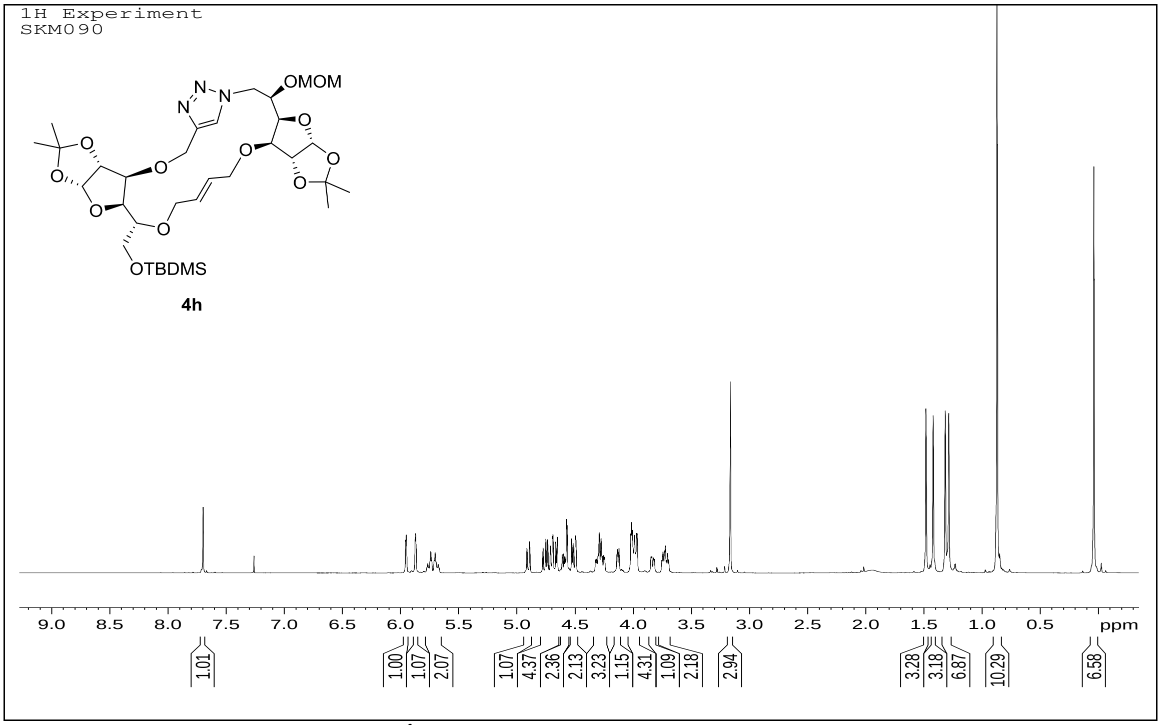

^{1}H NMR spectrum of compound 4h in $CDCl_3$

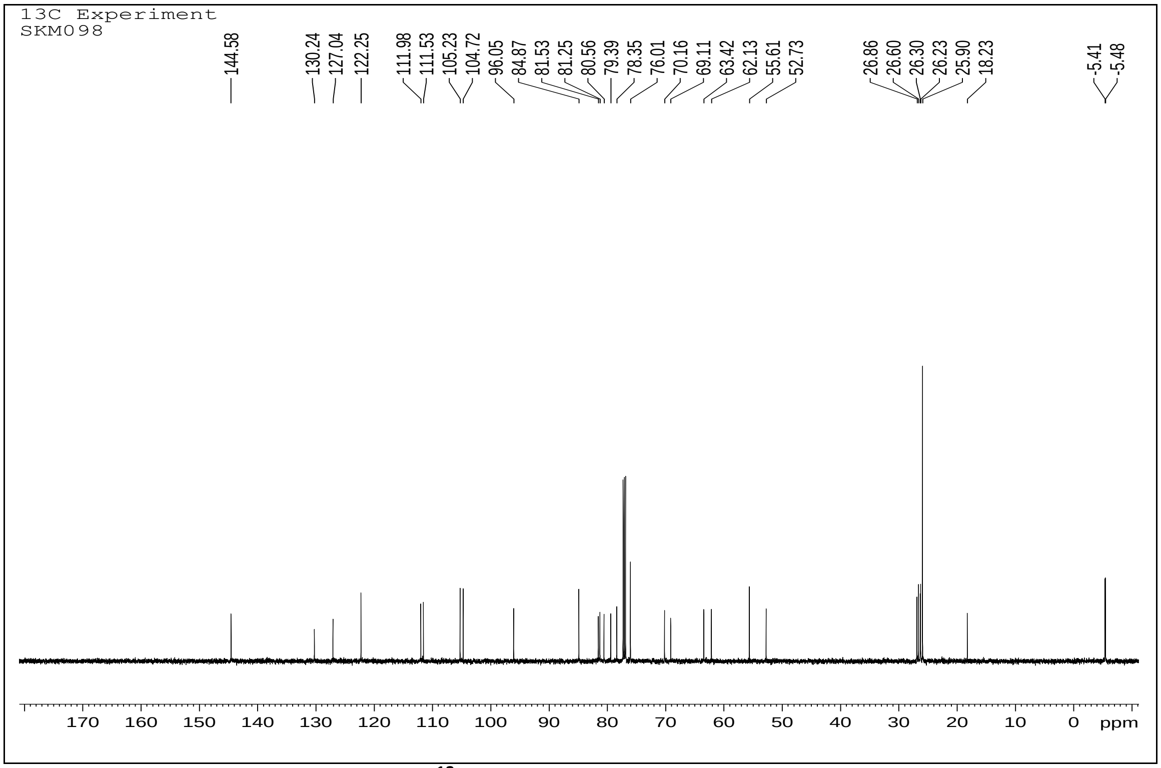

^{13}C spectrum of compound 4h in $CDCl_3$

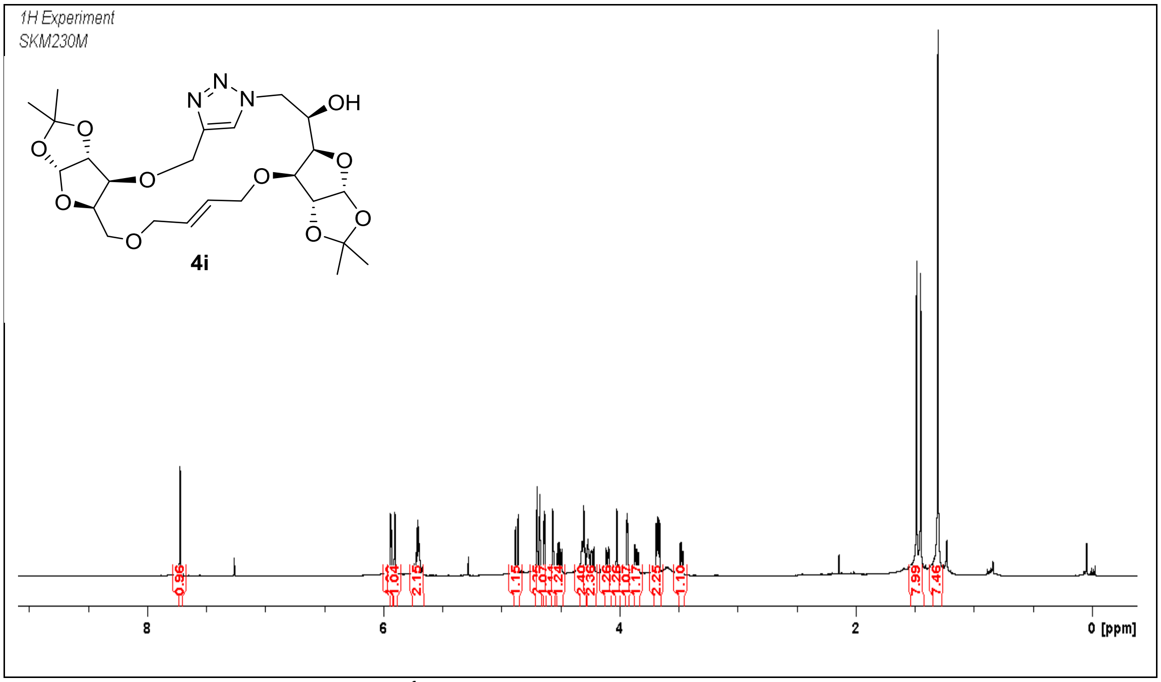

^{1}H NMR spectrum of compound 4i in $CDCl_3$

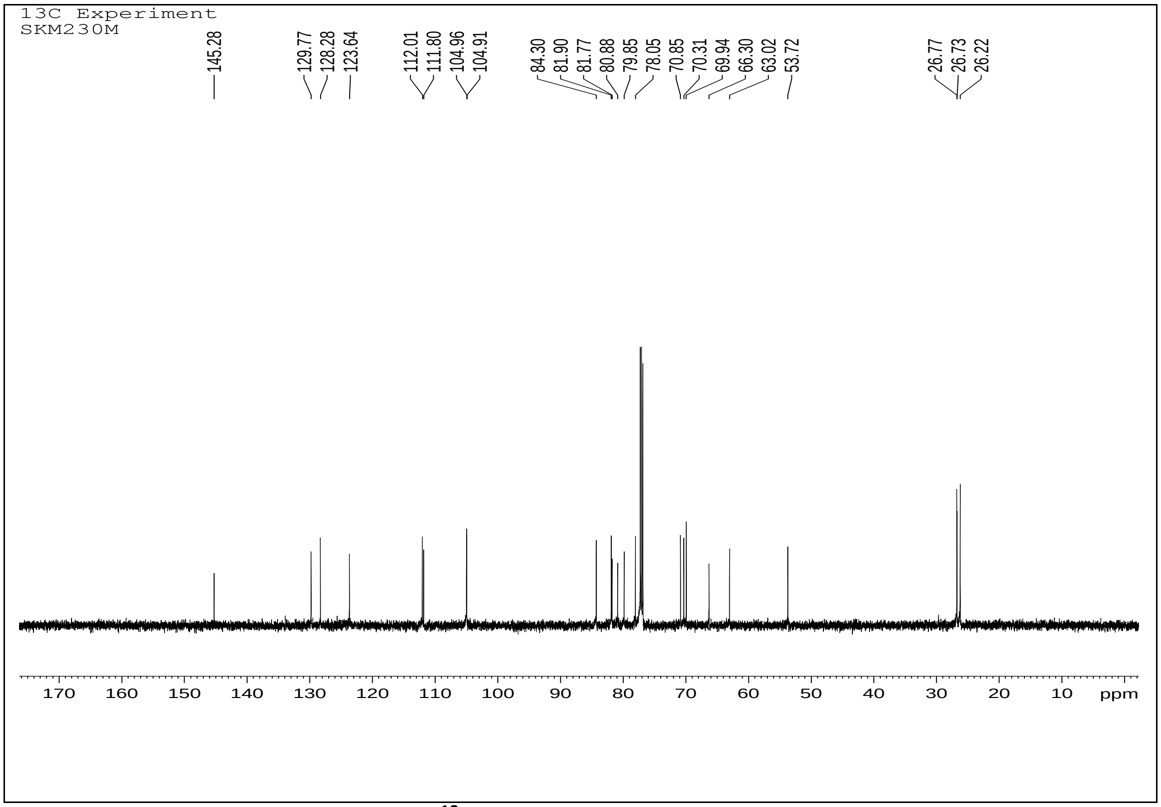

^{13}C spectrum of compound 4i in $CDCl_3$

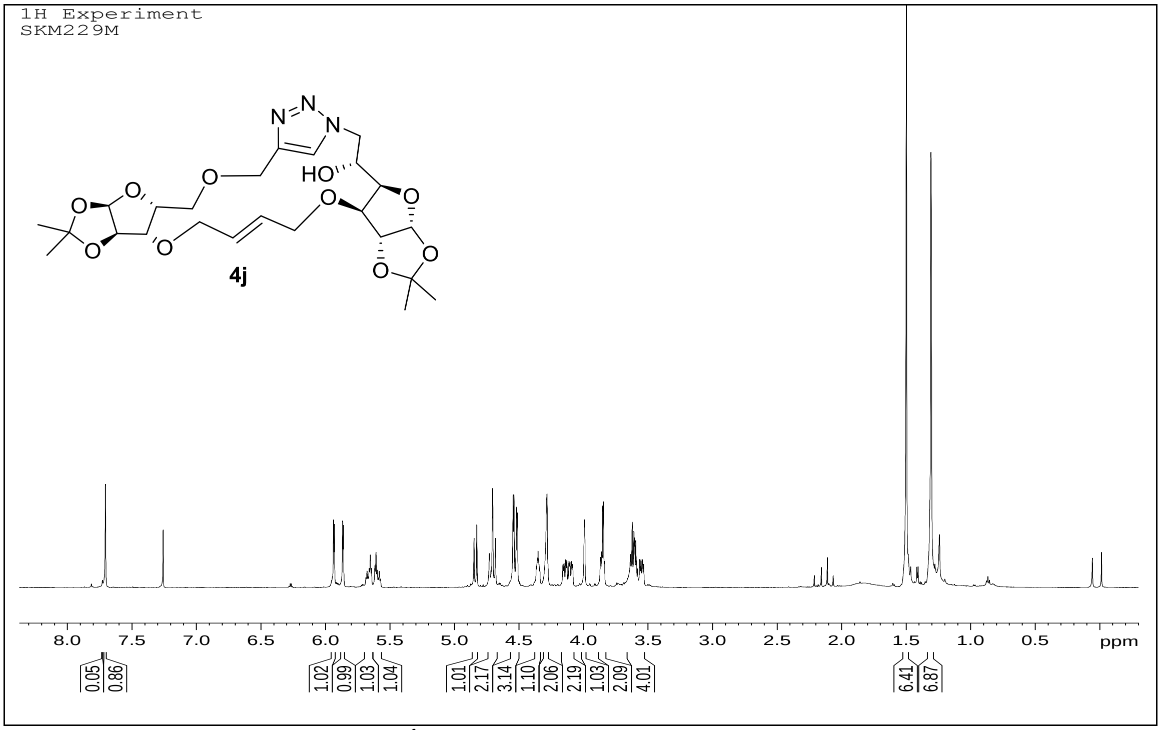

^{1}H NMR spectrum of compound 4j in CDCl$_3$

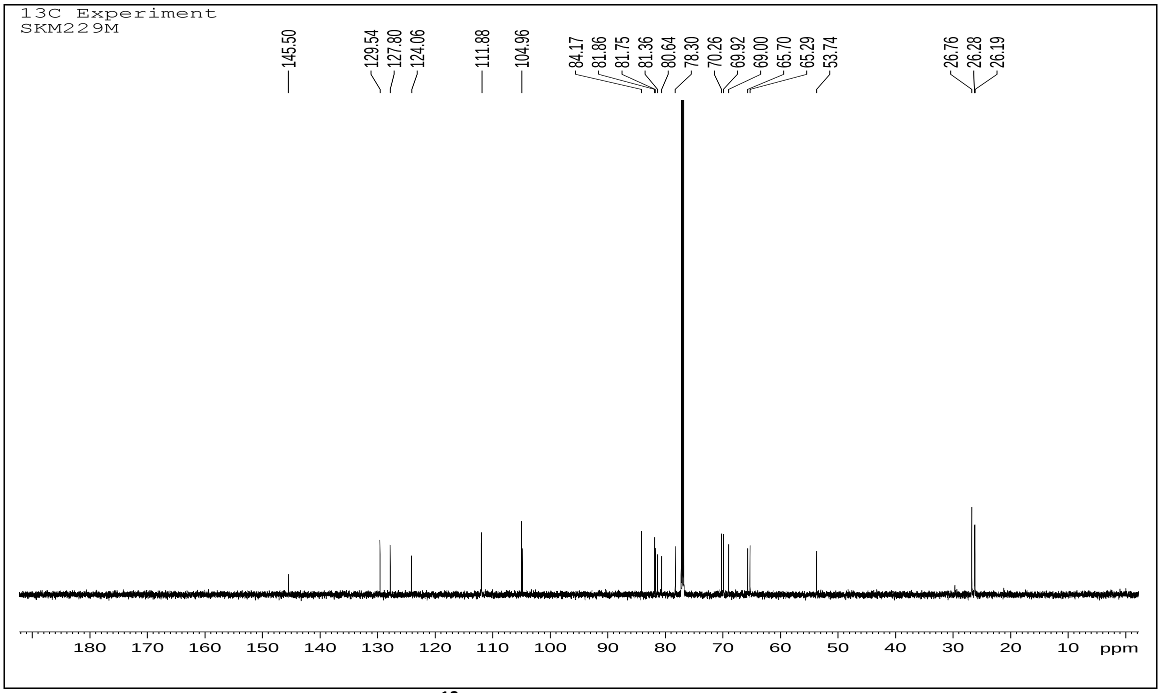

^{13}C spectrum of compound 4j in CDCl$_3$

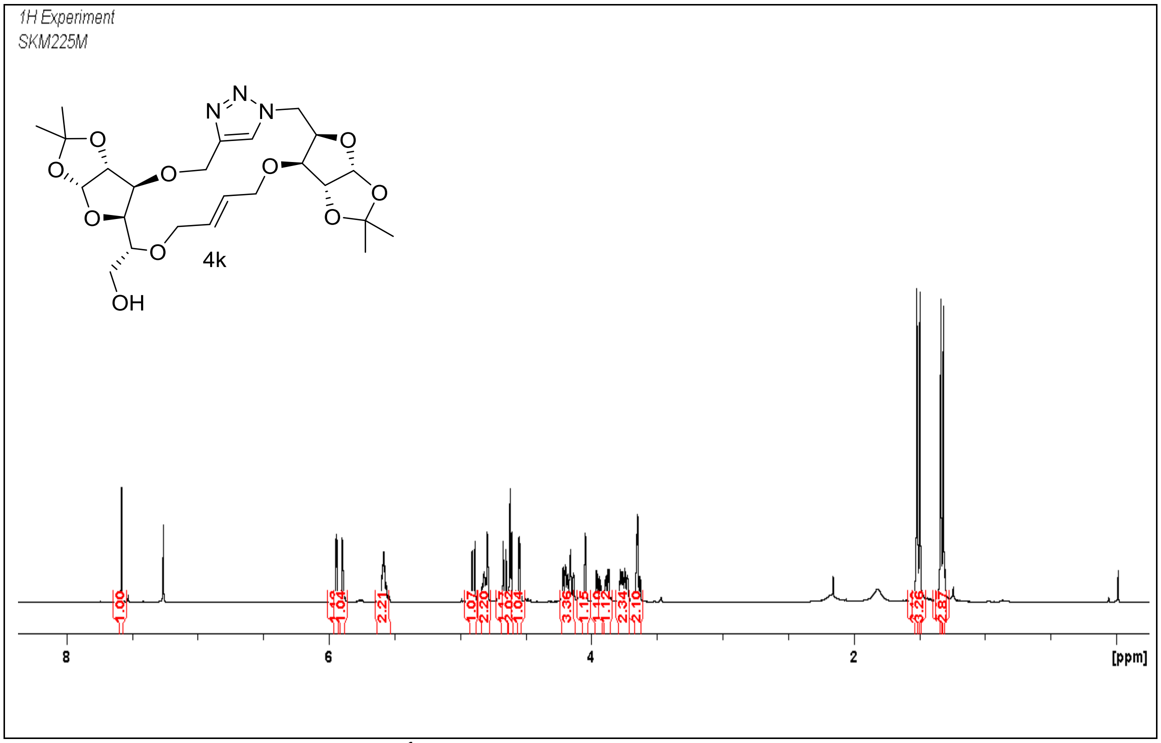

^{1}H NMR spectrum of compound 4k in $CDCl_3$

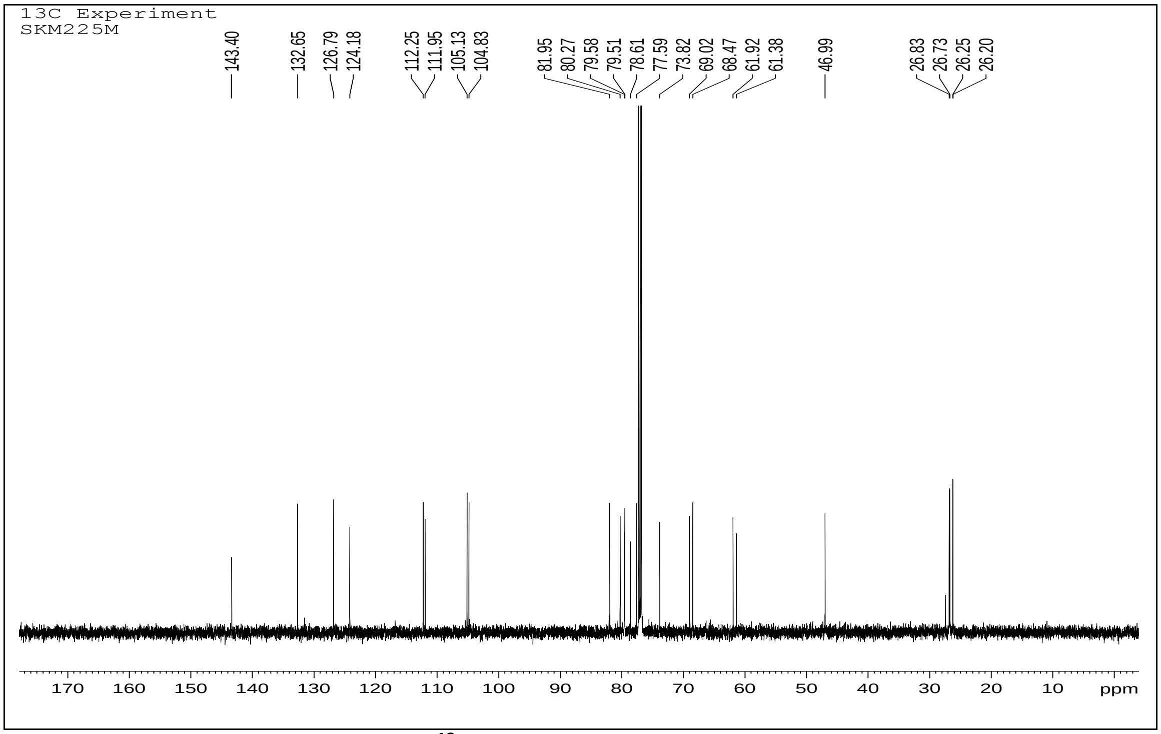

^{13}C spectrum of compound 4k in $CDCl_3$

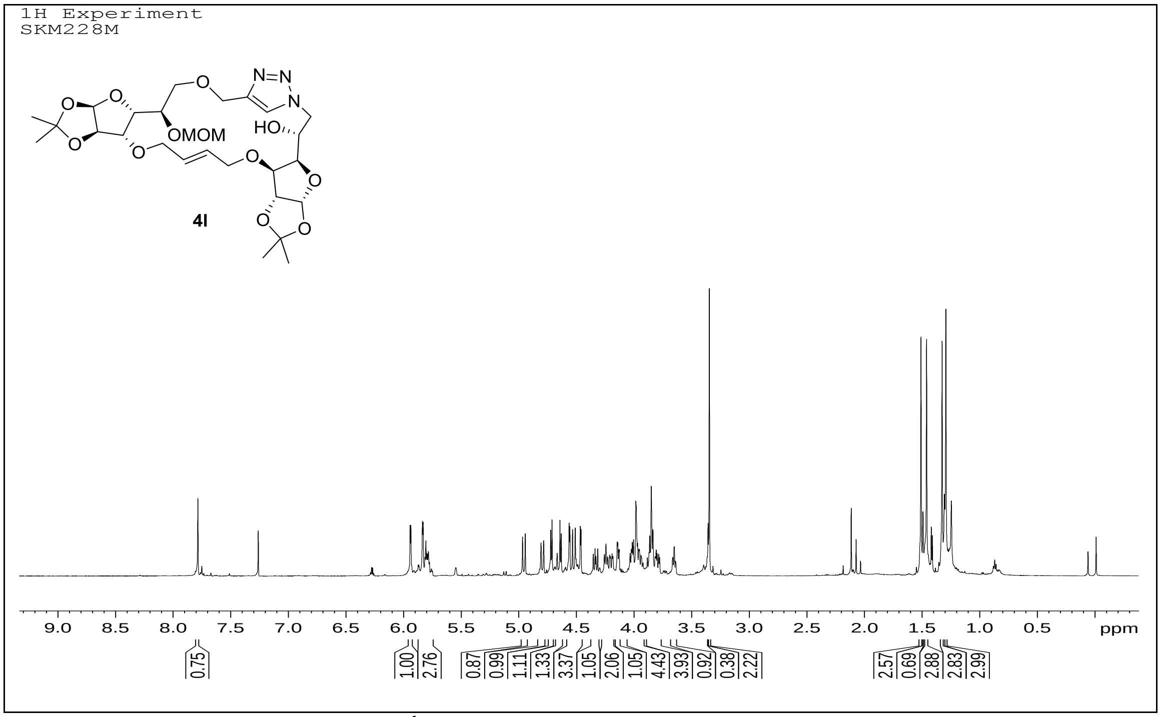

^{1}H NMR spectrum of compound 4l in $CDCl_3$

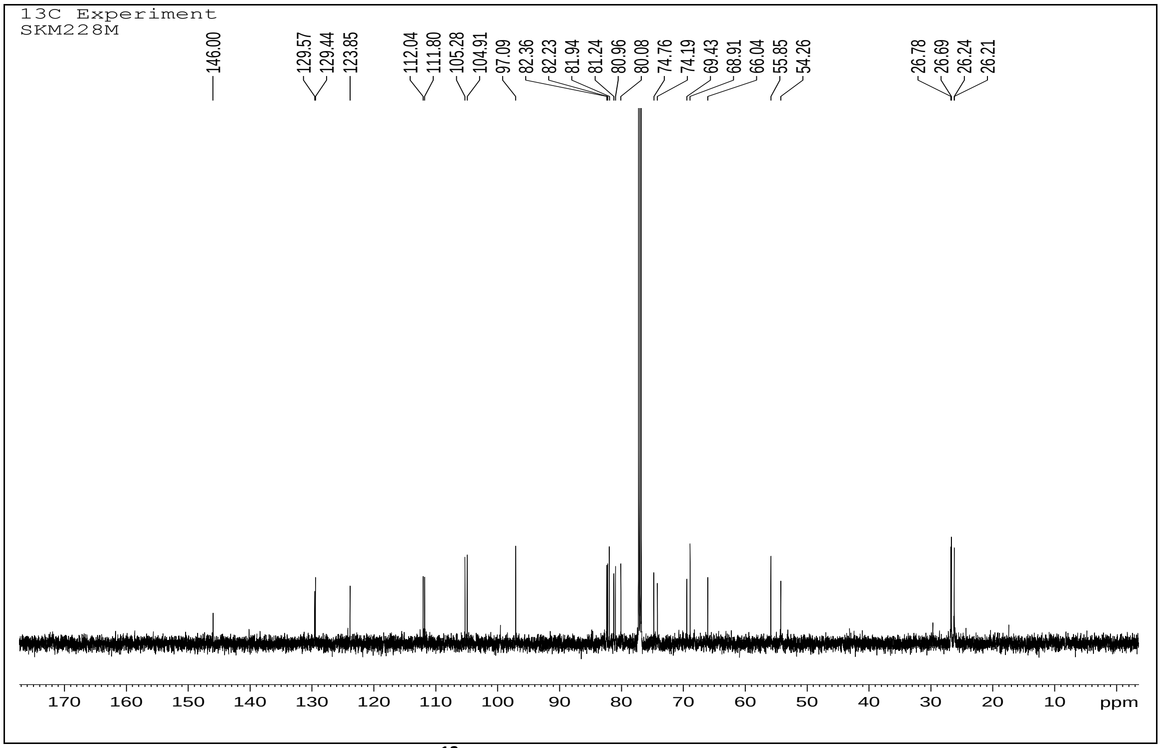

^{13}C spectrum of compound 4l in $CDCl_3$

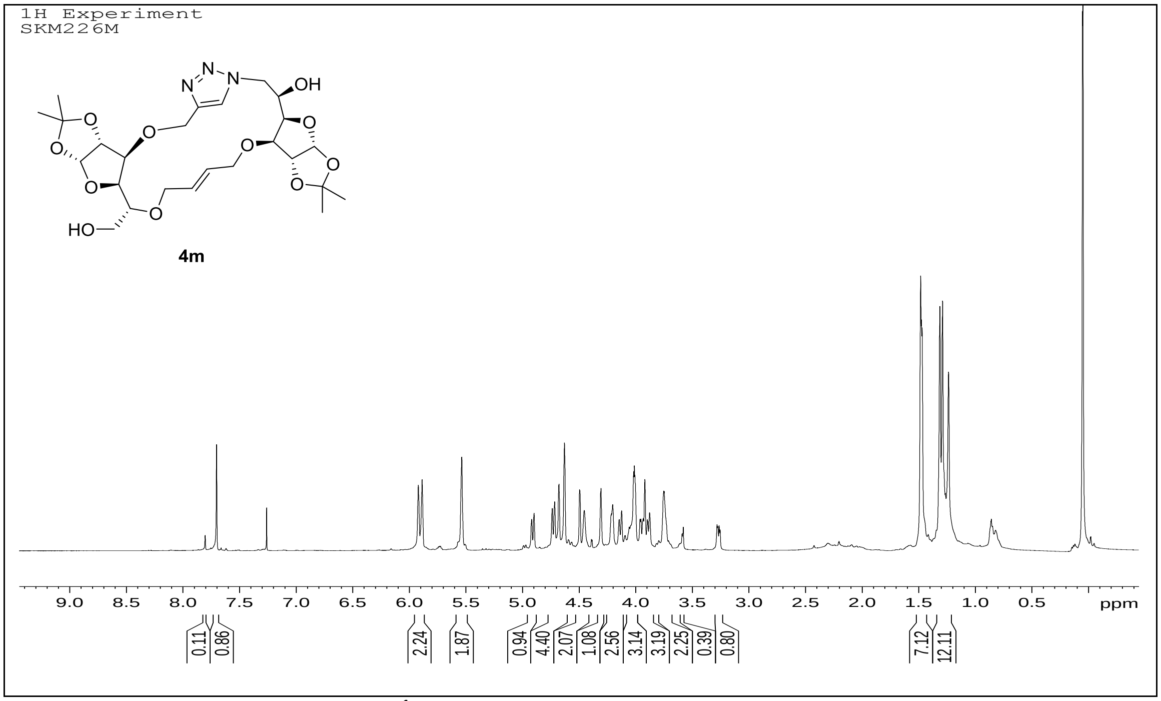

^{1}H NMR spectrum of compound 4m in $CDCl_3$

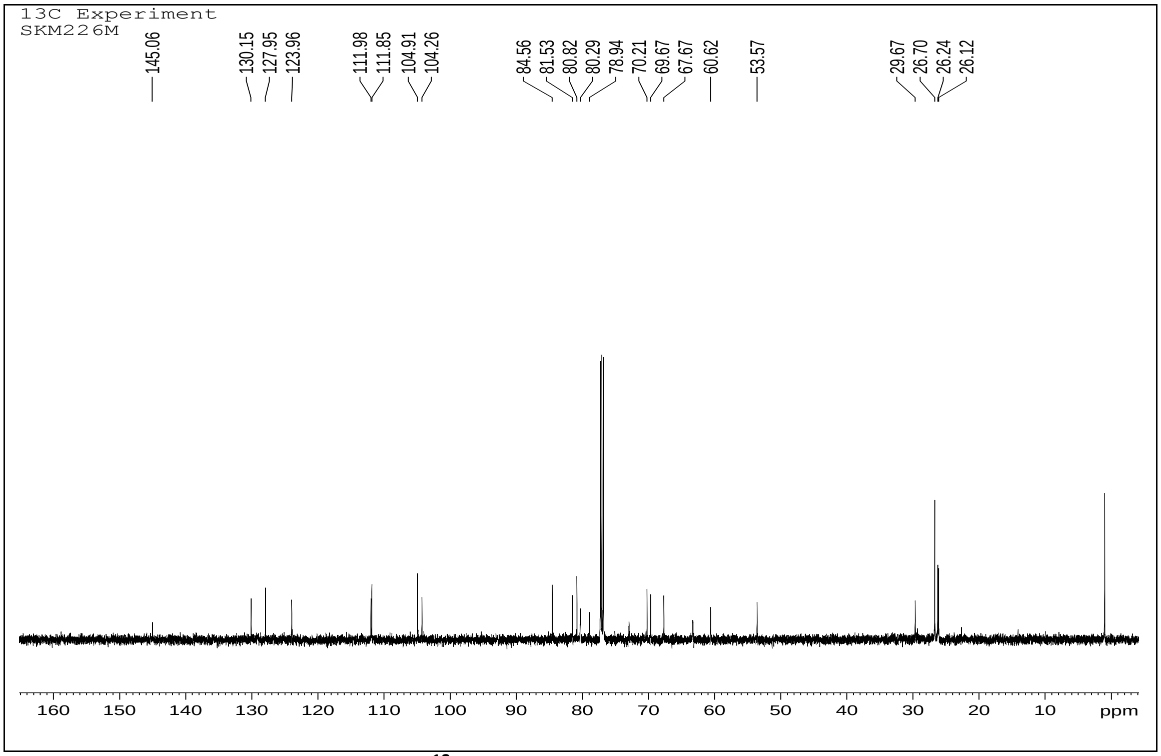

^{13}C spectrum of compound 4m in $CDCl_3$

NMR Spectrum of Diacetate 5 (S96)

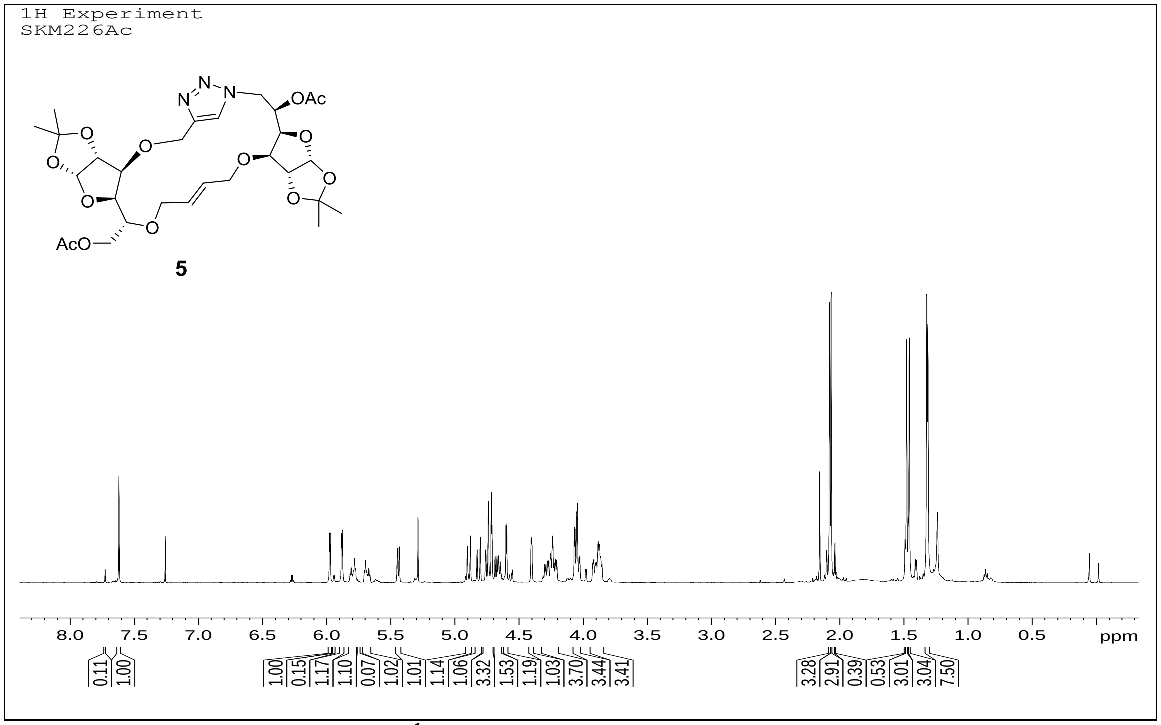

^{1}H NMR spectrum of compound 5 in $CDCl_3$

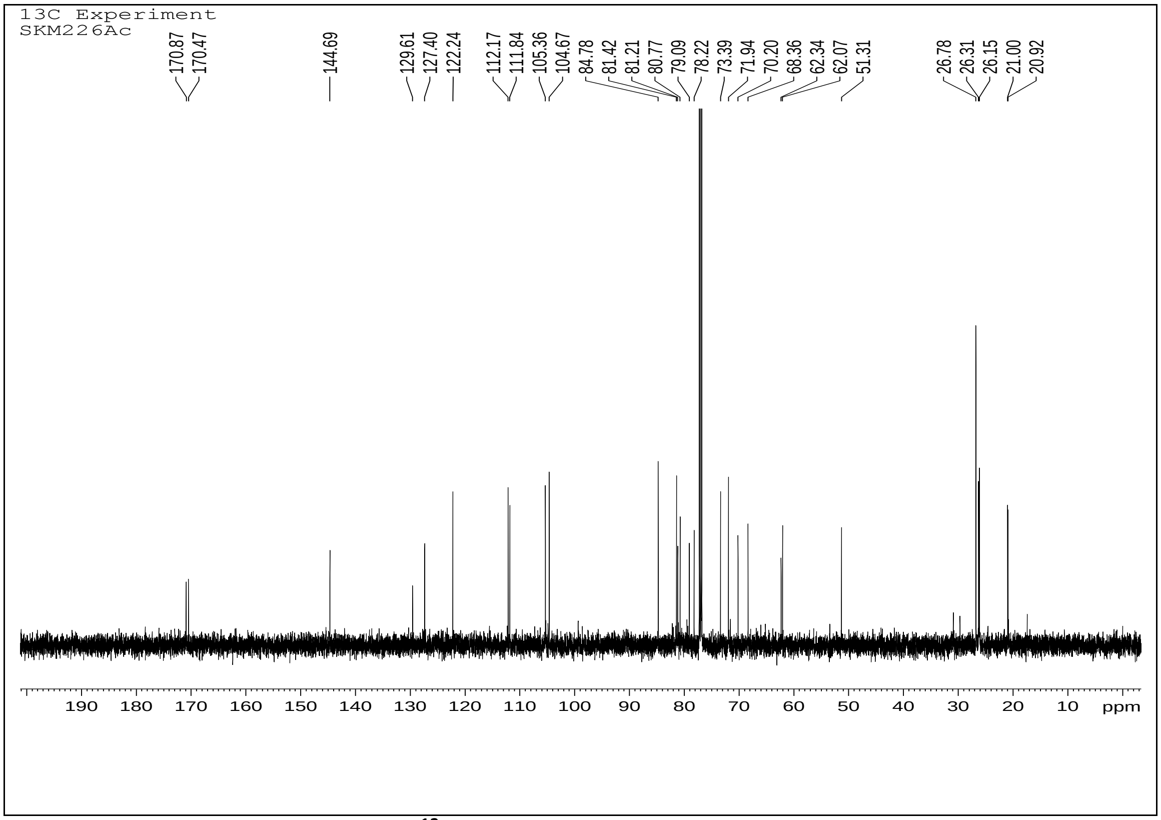

^{13}C spectrum of compound 5 in $CDCl_3$
